# The global, regional and national burden of three female pelvic cancers attributable to high BMI from 1990 to 2021: a systematic analysis for the Global Burden of Disease Study 2021 and projection to 2050

**DOI:** 10.1017/S0007114525105035

**Published:** 2025-09-14

**Authors:** Yi Jiang, Lijing Jiao, Ling Xu, Yabin Gong, Chenbing Sun

**Affiliations:** 1 Department of Gynecology and Obstetrics, Tongde Hospital of Zhejiang Province, Xihu District, Hangzhou 310012, Zhejiang Province, People’s Republic of China; 2 Department of Oncology, Yueyang Hospital of Integrated Traditional Chinese and Western Medicine, Shanghai University of Traditional Chinese Medicine, Hongkou District, Shanghai 200437, People’s Republic of China

**Keywords:** BMI, Pelvic neoplasms, Cancer burden, Obesity

## Abstract

High BMI is an important risk factor for female colon and rectal, ovarian and uterine cancers. Current comprehensive studies on its effects on these cancers are limited. This paper aims to explore regional and age differences in the impact of high BMI on these cancers and the commonalities among the three by using the Global Burden of Disease 2021. Deaths, disability-adjusted life years and their age-standardised rates for these cancers were retrieved from 1990 to 2021, and burden trends were assessed using the estimated annual percentage change and percentage changes. The study also analysed the correlation between age-standardised rate and socio-demographic index across twenty-one regions and projected future disease burden trends using the Bayesian Age-Period-Cohort model. Results showed that the global burden of female colon and rectal cancer declined since 1990 but remained at the highest level among the three cancers in 2021. At the same time, these three cancers had high burdens in high-income areas. Since 1990, ovarian and uterine cancer burdens attributable to high BMI increased, and all three burdens grew fastest in low-middle-income regions and among younger people. The burden of all three is projected to continue increasing through 2050. This study confirms that high BMI’s impact on these cancers is regional and age-specific, with long-term effects. Therefore, subsequent public health interventions should adopt more targeted obesity prevention and control strategies based on national and regional situations to effectively mitigate the adverse effects of high BMI on these cancers.

In recent years, colon and rectal, ovarian and uterine cancers have been rising in incidence and mortality rates^(1–5)^, significantly impacting human health globally. Given the many similarities in anatomical location and pathogenesis among ovarian cancer, uterine cancer and female colon and rectal cancer, we collectively refer to these cancers as ‘female pelvic cancers’. This naming can promote the in-depth development of interdisciplinary research, thereby conducting epidemiological studies more systematically and providing more precise and efficient strategic support for disease prevention work.

BMI is a widely used measure to assess obesity and overall health, offering insights into the risks for high blood pressure, diabetes, cancer and other chronic diseases^(6)^. With the global rise in obesity, high BMI has emerged as a major metabolic health concern, significantly increasing the risk of cancers^(7,8)^. Several studies have established that high BMI is a key risk factor for colon and rectal, ovarian and uterine cancers, and a clear linear correlation exists between BMI and the incidence of these cancers^(9–13)^. The Global Burden of Disease (GBD) database also showed that high BMI was a common risk factor for all three cancers. However, most present studies have focused on individual cancer types and have used outdated epidemiological data, lacking a comprehensive assessment of the combined effects of high BMI on these cancers^(13,14)^.

To address these questions, we hypothesised that there are regional and age differences in the effects of high BMI on these three cancers, with some commonalities. To test this hypothesis, we used the latest data from the GBD 2021 to conduct a comprehensive analysis of the burden of three cancers attributable to high BMI between 1990 and 2021 at global, regional, country and age-specific levels and projected health burden trends through 2050. The aim is to establish a scientific basis for developing more effective nutrition strategies to control weight and prevent and treat cancer.

## Methods and materials

### Data source

This study utilised information from the GBD 2021 database, which covers 371 diseases and injuries and 88 risk factors across 204 nations and 811 subnational locations from 1990 to 2021^(15,16)^. The GBD database employs a standardised methodology to evaluate the global and regional impacts of diseases, injuries and risk factors, stratified by age, sex and time. Further calculation details can be found at http://ghdx.healthdata.org/gbd-results-tool ^(17)^.

The GBD 2021 classifies risk factors into four levels, with high BMI identified as one of the metabolic risk factors. It applied Bradford Hill’s and the World Cancer Research Fund’s evidence standards to confirm a causal link between high BMI and ovarian, uterine, colon and rectum cancers^(18)^. A comparative risk assessment was used to estimate the cancer burden attributable to high BMI globally from 1990 to 2021, stratified by region and age.

### Definitions

High BMI in adults is defined as over 25 kg/m^2^. For children aged 1–19 years, it is defined by the International Obesity Task Force thresholds^(19)^.

### Statistical analyses

We assessed the burden of these three types of female pelvic cancer attributable to high BMI by calculating the number of deaths and disability-adjusted life years (DALY), age-standardised mortality rate (ASMR) per 100 000 population and age-standardised DALY rate (ASDR) per 100 000 population, along with their 95 % uncertainty intervals.

DALY is a measure of health loss and is the sum of years of life lost and years lived with disability. Years of life lost are the years of life lost due to premature death and are the difference between the life expectancy that could have been lived and the actual age at death. Years lived with disability refer to years of healthy life lost due to disability. The age-standardised rate (ASR) is a method to eliminate the differences in population composition indicators between different groups and adopt a unified standard population to standardise the rates of each group.

We analysed trends in ASR using the estimated annual percentage change (EAPC) and corresponding 95 % CI. The EAPC is calculated using a linear regression model on the natural logarithm of ASR against time. The formula is ln (ASR) = α +βx + ε, where x denotes the time variable and ε is the error term. EAPC = 100 × (exp(β) – 1) was used to estimate the EAPC and its 95 % CI^(20)^. EAPC value and lower bound of the 95 % CI are positive, indicating an upward trend. EAPC value and upper bound of the 95 % CI are negative, indicating a downward trend. Trends are statistically insignificant if the 95 % CI includes zero.

Based on the latest GBD 2021 data, the burden of these three cancers attributable to high BMI was analysed by age, year, five global socio-demographic index (SDI), 21 geographic regions and 204 countries. The SDI is based on income per capita, average years of schooling for women aged 15 and fertility rate for women under 25^(16,21)^. From the minimum to the maximum development level, the value of SDI ranges from 0 to 1. The five SDI regions are low SDI (< 0·47), low-middle SDI (0·47–0·62), middle SDI (0·62–0·71), high-middle SDI (0·71–0·81) and high SDI (> 0·81).

We also calculated percentage changes in death and DALY rates by age group from 1990 to 2021 and the associations between ASR and SDI for the three cancers attributable to high BMI across twenty-one regions. In this study, R version 4.4.1 was used for data analysis and calculation (https://www.R-project.org/), and the ggplot2 software package was used for data visualisation.

### Bayesian Age-Period-Cohort model

The Bayesian Age-Period-Cohort model is based on a Bayesian approach that incorporates complex interactions between age, period and cohort to more accurately predict future changes in disease burden. The significant advantage of this model is that it relies on the Integrated Nested Laplace Approximation to estimate the marginal posterior distribution, avoiding the mixing and convergence problems common in traditional Markov chain Monte Carlo methods. Therefore, the Bayesian Age-Period-Cohort model has been widely used in epidemiology^(22)^. In this study, the Bayesian Age-Period-Cohort package and Integrated Nested Laplace Approximation package in R Statistical software (version 4.4.1) were used for Bayesian Age-Period-Cohort analysis.

## Results

### Global burden of the three high BMI-attributed female pelvic cancers

**Fig. 1.**
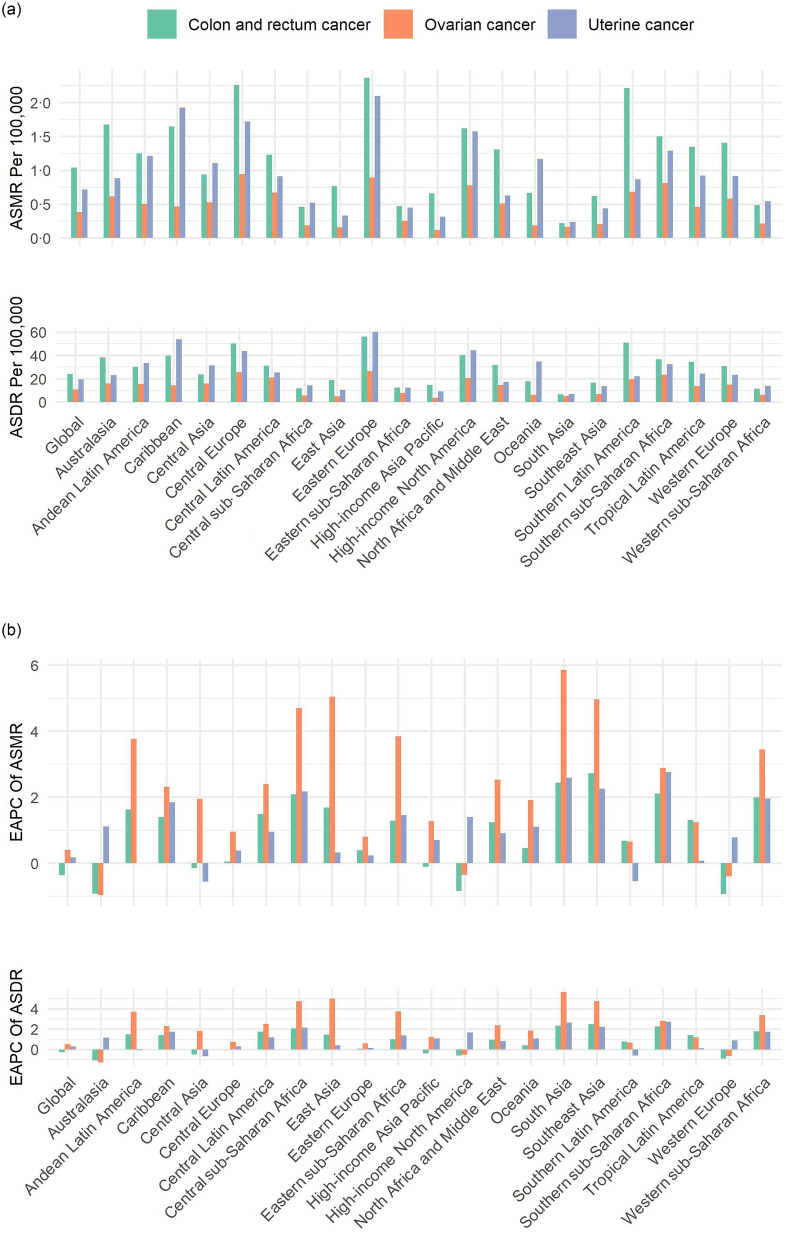
The ASMR (a) and ASDR (a) for the three female pelvic cancers attributable to high BMI in 2021, with corresponding EAPC (B) from 1990 to 2021, in global and twenty-one regions. ASMR, age-standardised mortality rate; ASDR, age-standardised DALY rate; DALY, disability-adjusted life years; EAPC, estimated annual percentage change.

**Fig. 2.**
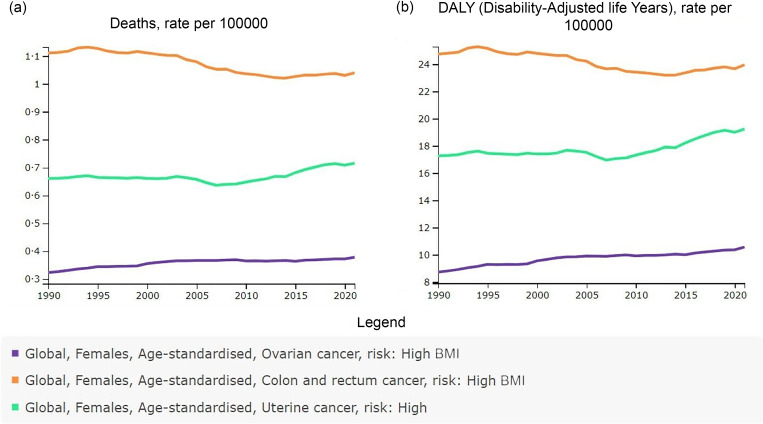
Temporal trends in ASMR (a) and ASDR (b) of the three female pelvic cancers attributable to high BMI from 1990 to 2021, in global. Graphic source: GBD study 2021, VizHub – GBD Results (Internet) (accessed 29 July 2024). (Available from: http://ghdx.healthdata.org/gbd-results-tool). ASMR, age-standardised mortality rate; ASDR, age-standardised DALY rate; DALY, disability-adjusted life years.

In 2021, approximately 98 770 deaths were attributable to high BMI for the three types of female pelvic cancers globally, representing about 30·99 % of all female pelvic cancer deaths (online Supplementary Table S1). Of these, high BMI-attributed deaths for female colon and rectum, ovarian and uterine cancers were 48 292 cases, 17 344 cases and 33 134 cases, respectively, all representing an increase compared to 1990 (Table 1). In 2021, the corresponding ASMR per 100 000 population were 1·04 for female colon and rectum cancer, 0·38 for ovarian cancer and 0·72 for uterine cancer. The DALY and corresponding ASDR per 100 000 for the three high BMI-attributed female pelvic cancers were 1 095 773 (23·96) for female colon and rectum cancer, 477 248 (10·56) for ovarian cancer and 880 147 (19·23) for uterine cancer (Table 1). Globally, high BMI-attributed female colon and rectum cancer had the highest mortality and DALY in both 1990 and 2021, followed by uterine and ovarian cancers (Table 1 and Fig. 1(a)). However, from 1990 to 2021, the ASMR and ASDR for high BMI-attributed female colon and rectum cancer declined, with the EAPC values of –0·37 (95 % CI –0·42, –0·31) for ASMR and –0·26 (95 % CI –0·31, –0·20) for ASDR (Table 1; Figs. 1(b) and 2). In contrast, ovarian and uterine cancers attributable to high BMI showed increases in ASMR and ASDR. The EAPC for ASMR were 0·40 (95 % CI 0·33, 0·47) for ovarian cancer and 0·18 (95 % CI 0·08, 0·28) for uterine cancer, while the EAPC for ASDR were 0·51 (95 % CI 0·46, 0·56) for ovarian cancer and 0·27 (95 % CI 0·18, 0·37) for uterine cancer (Table 1; Figs. 1(b) and 2).

**Table 1.** Deaths and DALY for the three types of female pelvic cancers attributable to high BMI in 1990 and 2021, with corresponding EAPC from 1990 to 2021, in global

	Deaths										DALY									
Types	Number of cases, 1990	95 % UI	ASMR per 100 000, 1990	95 % UI	Number of cases, 2021	95 % UI	ASMR per 100 000, 2021	95 % UI	EAPC of ASMR, 1990–2021	95 % CI	Number of cases, 1990	95 % UI	ASDR per 100 000, 1990	95 % UI	Number of cases, 2021	95 % UI	ASDR per 100 000, 2021	95 % UI	EAPC of ASDR, 1990–2021	95 % CI
Colon and rectum cancer	22 586	9703, 36765	1·11	0·48, 1·81	48 292	20914, 76782	1·04	0·45, 1·65	–0·37	–0·42, –0·31	525 776	225045, 849419	24·73	10·59, 39·99	1 095 773	474290, 1727956	23·96	10·36, 37·75	–0·26	–0·31, –0·20
Ovarian cancer	6850	1423, 12865	0·32	0·07, 0·61	17 344	4141, 30810	0·38	0·09, 0·67	0·40	0·33, 0·47	188 874	38401, 355691	8·72	1·78, 16·41	477 248	113449, 840002	10·56	2·50, 18·57	0·51	0·46, 0·56
Uterine cancer	13 893	9874, 18653	0·66	0·47, 0·89	33 134	23878, 43299	0·72	0·52, 0·94	0·18	0·08, 0·28	372 641	264224, 500197	17·26	12·25, 23·16	880 147	631165, 1160930	19·23	13·80, 25·38	0·27	0·18, 0·37

DALY, disability-adjusted life years; ASMR, age-standardised mortality rate; ASDR, age-standardised DALY rate; EAPC, estimated annual percentage change; UI, uncertainty interval.

### The burden of the three high BMI-attributed female pelvic cancers by socio-demographic index and geographic regions

In 2021, the highest number of deaths from high BMI-attributed female colon and rectum, ovarian and uterine cancers occurred in the high SDI category. However, the highest ASMR per 100 000 population was reported in the high-middle SDI for colon and rectum cancer (1·44), in the high SDI for ovarian cancer (0·57) and in the high SDI for uterine cancer due to high BMI (1·02). Additionally, the highest DALY and ASDR per 100 000 population were recorded in the high-middle SDI for high BMI-attributed female colon and rectum cancer (DALY 354 770, ASDR 33·25), in the high SDI for ovarian cancer (DALY 144 449, ASDR 15·13) and uterine cancer (DALY 284 156, ASDR 27·91) (online Supplementary Table S2–S4). From 1990 to 2021, the increase in ASMR and ASDR for these cancers attributable to high BMI was most significant in low-middle SDI regions, showing the highest EAPC values. Conversely, between 1990 and 2021, the ASMR for female colon and rectum cancer attributable to high BMI declined significantly in high SDI regions (EAPC −0·88, 95 % CI −0·94, −0·82). Similarly, its ASDR decreased significantly in both high SDI regions (EAPC −0·70, 95 % CI −0·75, −0·65) and high-middle SDI regions (EAPC −0·24, 95 % CI −0·32, −0·16). For ovarian cancer attributable to high BMI, both ASMR and ASDR decreased in high SDI regions, with the EAPC of −0·36 (95 % CI −0·49, −0·23) for ASMR and −0·43 (95 % CI −0·56, −0·3) for ASDR. Uterine cancer attributable to high BMI saw decreases in ASMR and ASDR in high-middle SDI regions, with the EAPC of −0·37 (95 % CI −0·51, −0·23) for ASMR and −0·44 (95 % CI −0·59, −0·29) for ASDR (online Supplementary Table S2–S4 and Figs. S1–S3).

In 2021, the number of deaths and DALY for these three cancers attributable to high BMI were higher in East Asia, Western Europe, Eastern Europe and High-income North America among twenty-one regions (Fig. 1(a) and online Supplementary Table S2–S4). The highest ASMR for female colon and rectum cancer and uterine cancer was recorded in Eastern Europe, while Central Europe had the highest ASMR for ovarian cancer. Eastern Europe also recorded the highest ASDR for all three cancers. From 1990 to 2021, the largest increases in ASMR and ASDR for female colon and rectum and uterine cancers attributable to high BMI were observed in Southeast Asia, South Asia and Southern sub-Saharan Africa, with the highest EAPC values. The EAPC values of ovarian cancer were the highest in Southeast Asia, South Asia and Central Asia. During the same period, notable declines in ASMR and ASDR for female colon and rectum cancer associated with high BMI were observed in Western Europe, Australasia, High-income North America, Central Asia and High-income Asia Pacific, while ASDR also decreased in Central Europe. For ovarian cancer cases attributed to high BMI, both the ASMR and the ASDR declined in High-income North America, Western Europe and Australasia. For uterine cancer, ASMR and ASDR declined in Central Asia and Southern Latin America, as did ASDR in Andean Latin America (Fig. 1(b) and online Supplementary Table S2–S4).

### The burden of the three high BMI-attributed female pelvic cancers at the national level

**Fig. 3.**
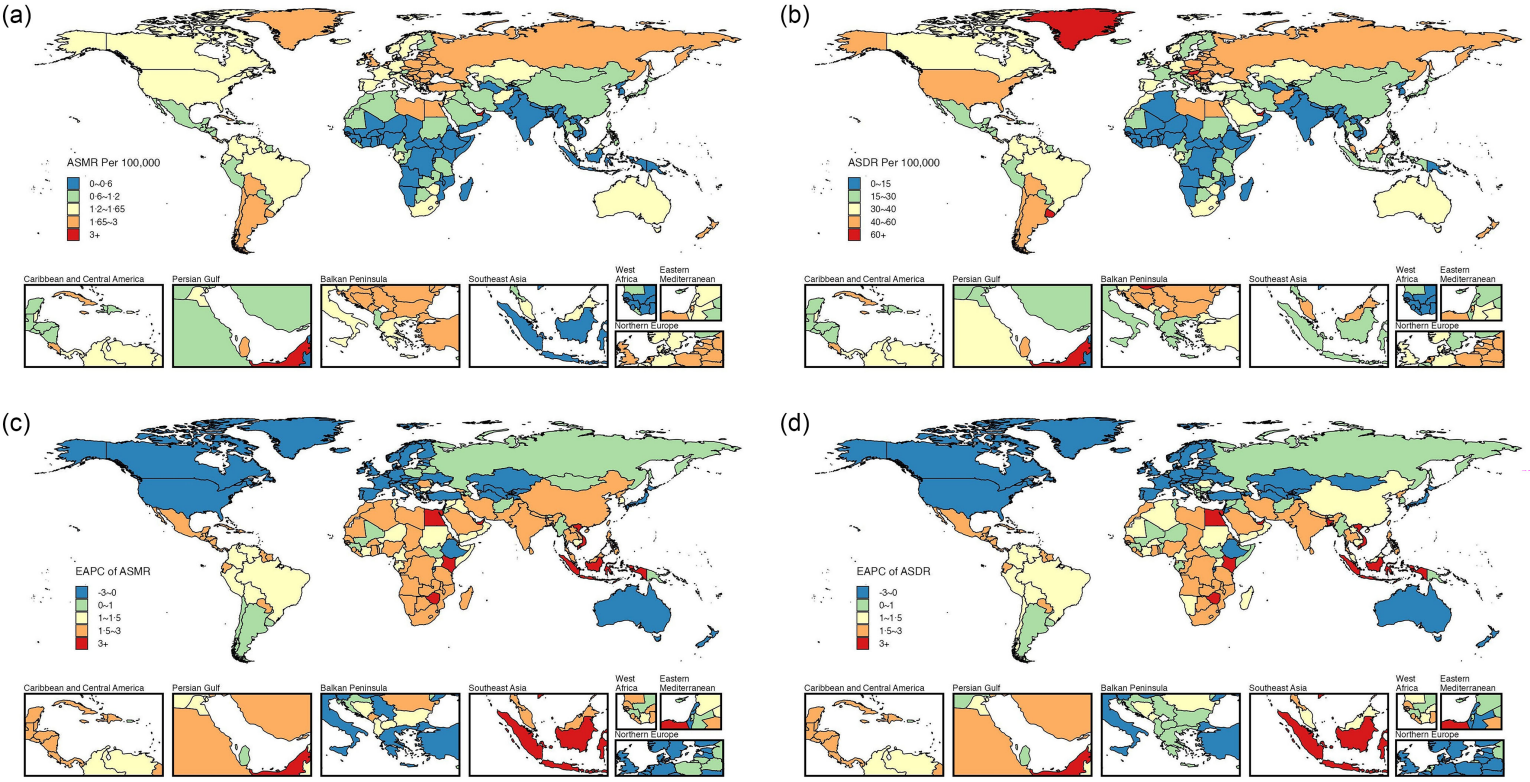
The ASMR (a) and ASDR (b) for female colon and rectum cancer attributable to high BMI in 2021, with corresponding EAPC (c) and (d) from 1990 to 2021, in 204 countries. ASMR, age-standardised mortality rate; ASDR, age-standardised DALY rate; DALY, disability-adjusted life years; EAPC, estimated annual percentage change.

**Fig. 4.**
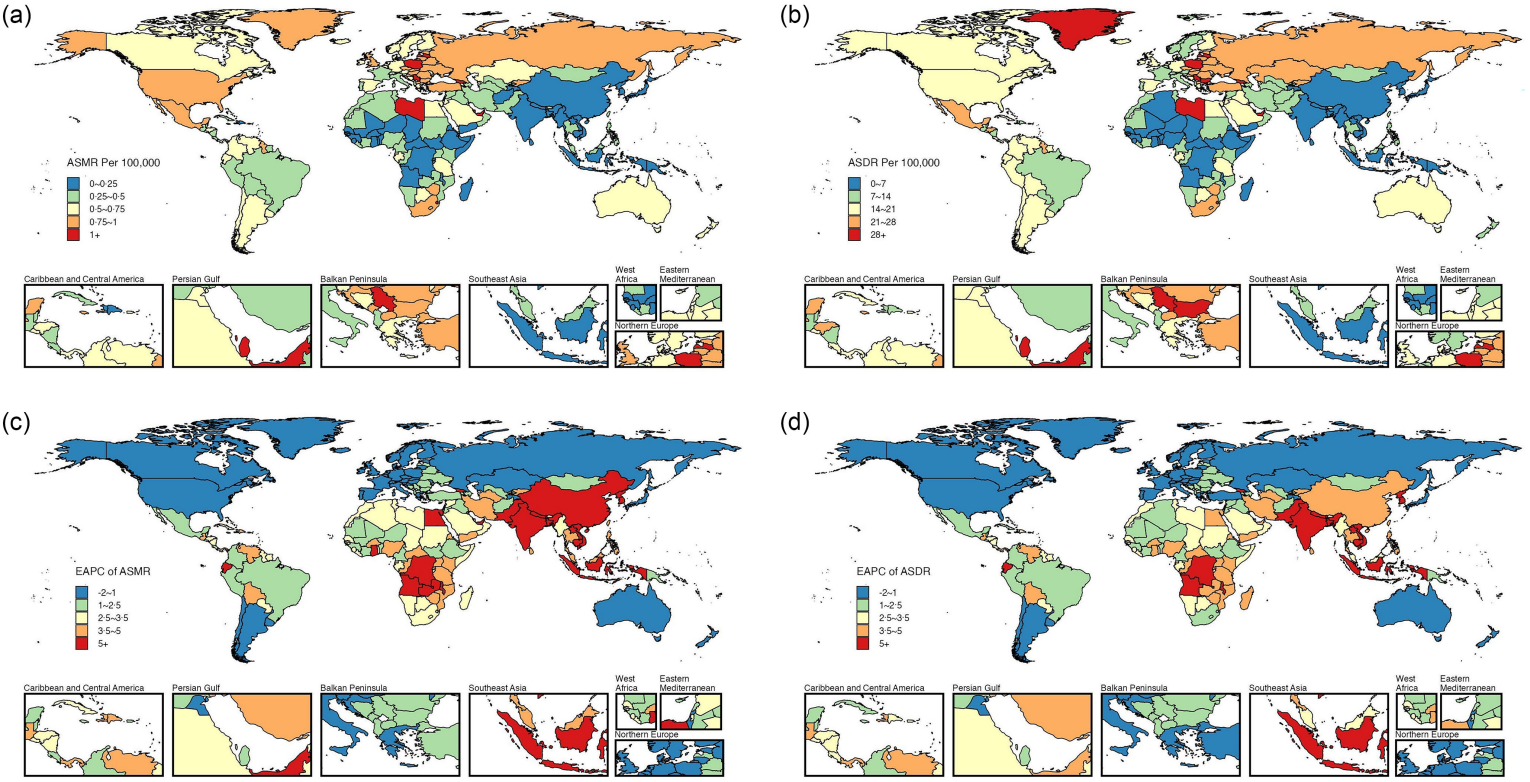
The ASMR (a) and ASDR (b) for female ovarian cancer attributable to high BMI in 2021, with corresponding EAPC (c) and (d) from 1990 to 2021, in 204 countries. ASMR, age-standardised mortality rate; ASDR, age-standardised DALY rate; DALY, disability-adjusted life years; EAPC, estimated annual percentage change.

**Fig. 5.**
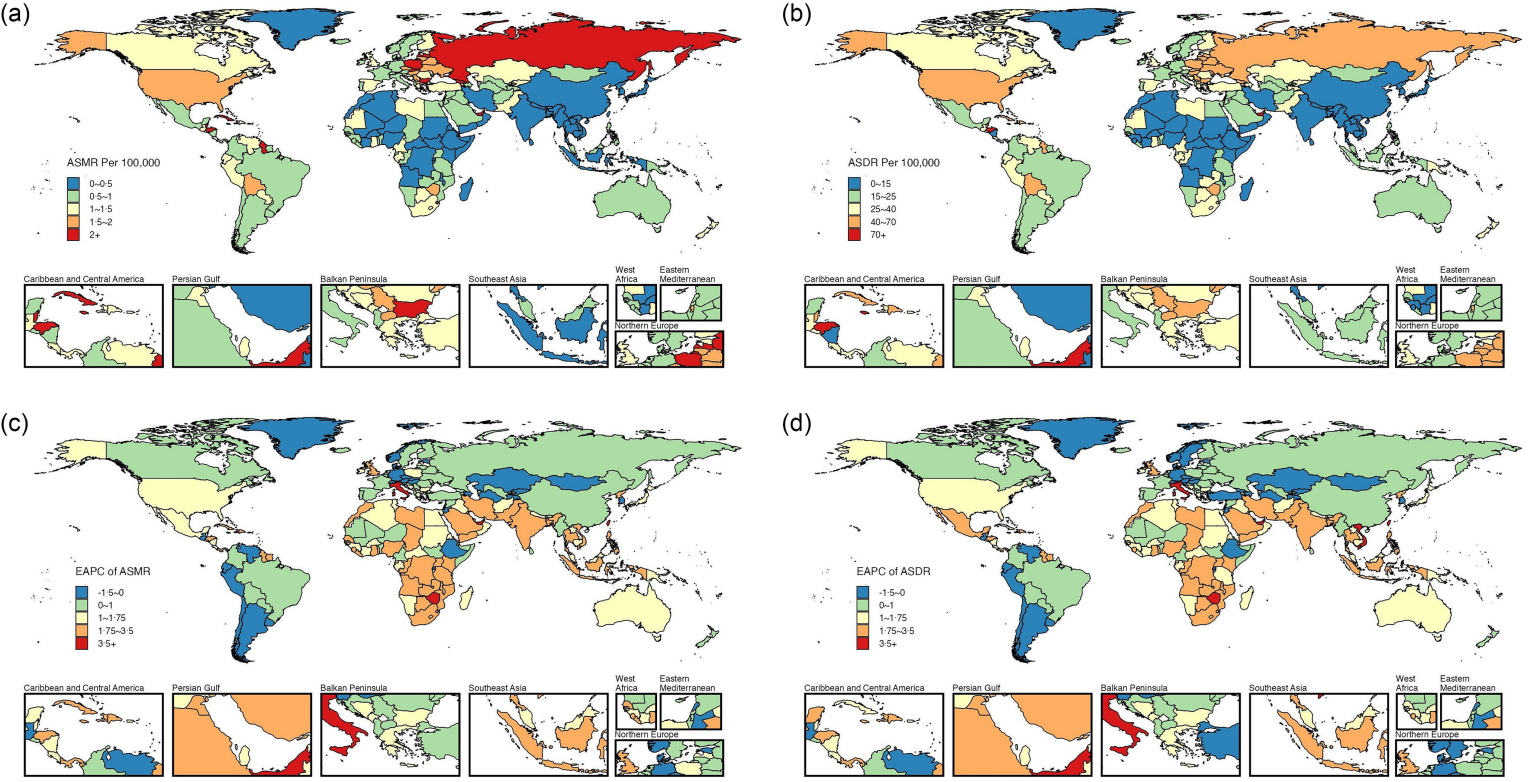
The ASMR (a) and ASDR (b) for female uterine cancer attributable to high BMI in 2021, with corresponding EAPC (c) and (d) from 1990 to 2021, in 204 countries. ASMR, age-standardised mortality rate; ASDR, age-standardised DALY rate; DALY, disability-adjusted life years; EAPC, estimated annual percentage change.

In 2021, the highest ASMR and ASDR for these three cancers attributable to high BMI were recorded in the United Arab Emirates (online Supplementary Table S5–S7; Fig. 3(a), (b); Fig. 4(a), (b); Fig. 5(a), (b)). From 1990 to 2021, female colon and rectum cancer and uterine cancer attributable to high BMI in the United Arab Emirates had the most significant increases in ASMR, with the EAPC values of 4·84 (95 % CI 4·18, 5·51) for female colon and rectum cancer and 4·95 (95 % CI 4·43, 5·47) for uterine cancer. Timor-Leste also exhibited a remarkable increase in ASMR for high BMI-attributed ovarian cancer, with an EAPC of 22·88 (95 % CI 19·02, 26·86) (online Supplementary Table S5–S7; Figs. 3(c), 4(c) and 5(c)). Countries with substantial increases in ASDR for these three cancers attributable to high BMI included Viet Nam, Timor-Leste and Zimbabwe, with the EAPC of 4·54 (95 % CI 4·3, 4·77), 18·09 (95 % CI 16·05, 20·16) and 4·68 (95 % CI 3·86, 5·52), respectively (online Supplementary Table S5–S7; Figs. 3(d), 4(d) and 5(d)). Between 1990 and 2021, Austria, Germany and Greenland showed the most significant declines in ASMR and ASDR for female colon and rectum cancer attributable to high BMI. For ovarian cancer, Greenland had the largest decrease in ASMR, followed by Germany and New Zealand, while Sweden, New Zealand and Germany reported the most significant reductions in ASDR. Furthermore, ASMR for uterine cancer attributable to high BMI decreased notably in Greenland, Ethiopia and the Republic of Korea, while ASDR also declined in Ethiopia, Greenland and the Maldives (online Supplementary Table S5–S7; Fig. 3(c), (d); Fig. 4(c), (d); Fig. 5(c), (d)).

### Age group differences in the burden of the three high BMI-attributed female pelvic cancers

**Fig. 6.**
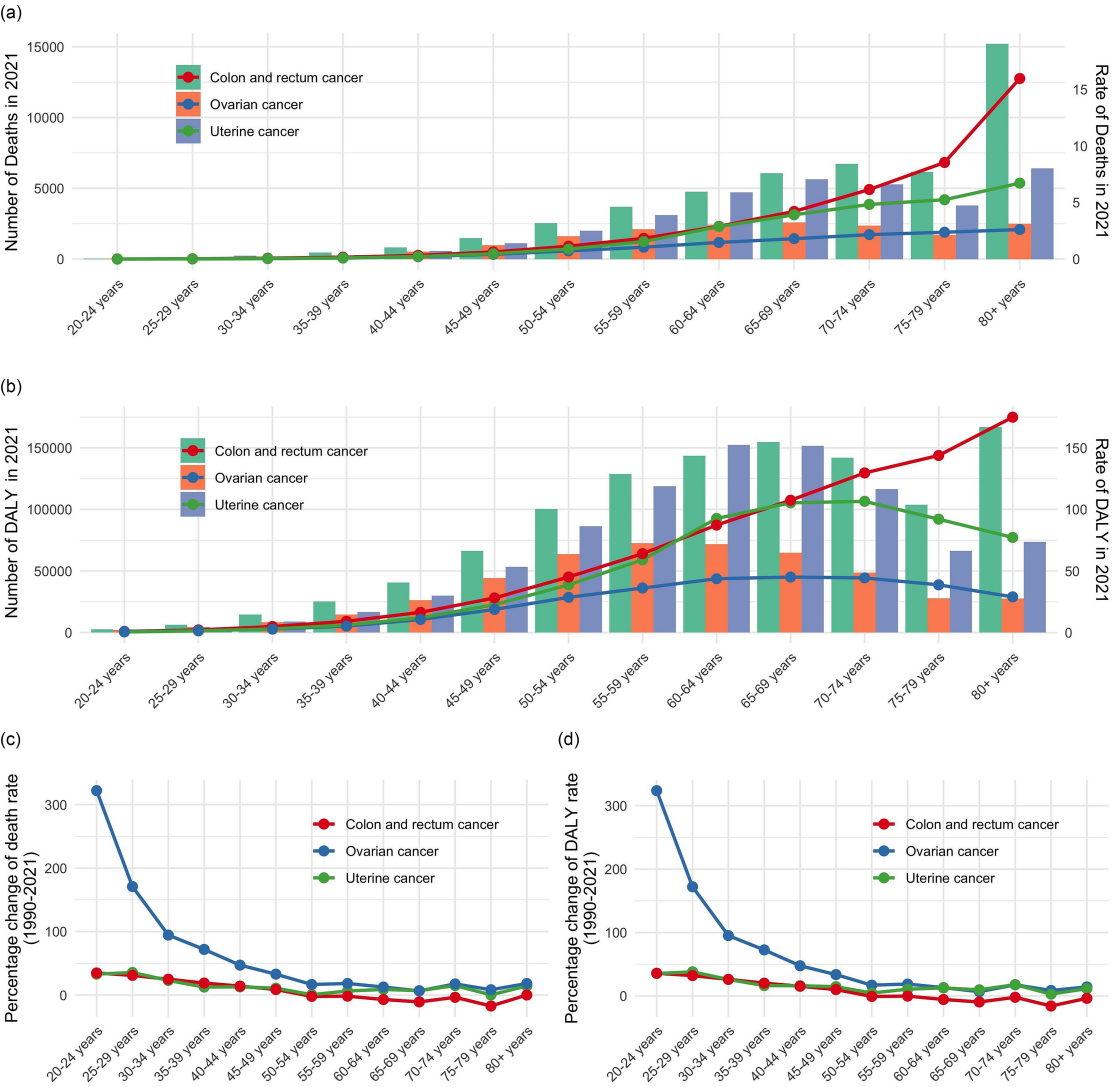
The numbers (bar) and rates (line) of deaths (a) and DALY (b) for the three female pelvic cancers attributable to high BMI by age group in 2021, with corresponding percentage changes (c) and (d) from 1990 to 2021. DALY, disability-adjusted life years.

The GBD 2021 database did not provide data on these three cancers attributable to high BMI for individuals under 20 years of age. Consequently, the analysis began with individuals aged 20 and older, divided into thirteen age groups (Fig. 6). In 2021, global deaths from female colon and rectum cancer and uterine cancer attributed to high BMI all peaked in individuals aged 80 years and older, while the highest number of deaths from ovarian cancer occurred in the 65–69 age group (Fig. 6(a)). The age groups with the highest DALY varied among the three cancers attributable to high BMI. The colon and rectum cancer had the highest DALY in the 80+ age group, ovarian cancer in the 55–59 age group and uterine cancer in the 60–64 age group (Fig. 6(b)). Death rates for the three cancers attributable to high BMI and DALY rates for colon and rectum cancer increased with age. However, DALY rates for ovarian and uterine cancer due to high BMI peaked at 65–69 and 70–74, respectively (Fig. 6(a), (b)). From 1990 to 2021, percentage changes in death and DALY rates for all three cancers attributable to high BMI generally decreased with age, with the most significant increases seen in the 20–24 and 25–29 age groups. In these two age groups, ovarian cancer showed a significantly larger increase compared with colon and rectum cancer and uterine cancer (Fig. 6(c), (d)).

### The associations between age-standardised rate and socio-demographic index for the three high BMI-attributed female pelvic cancers across twenty-one regions

**Fig. 7.**
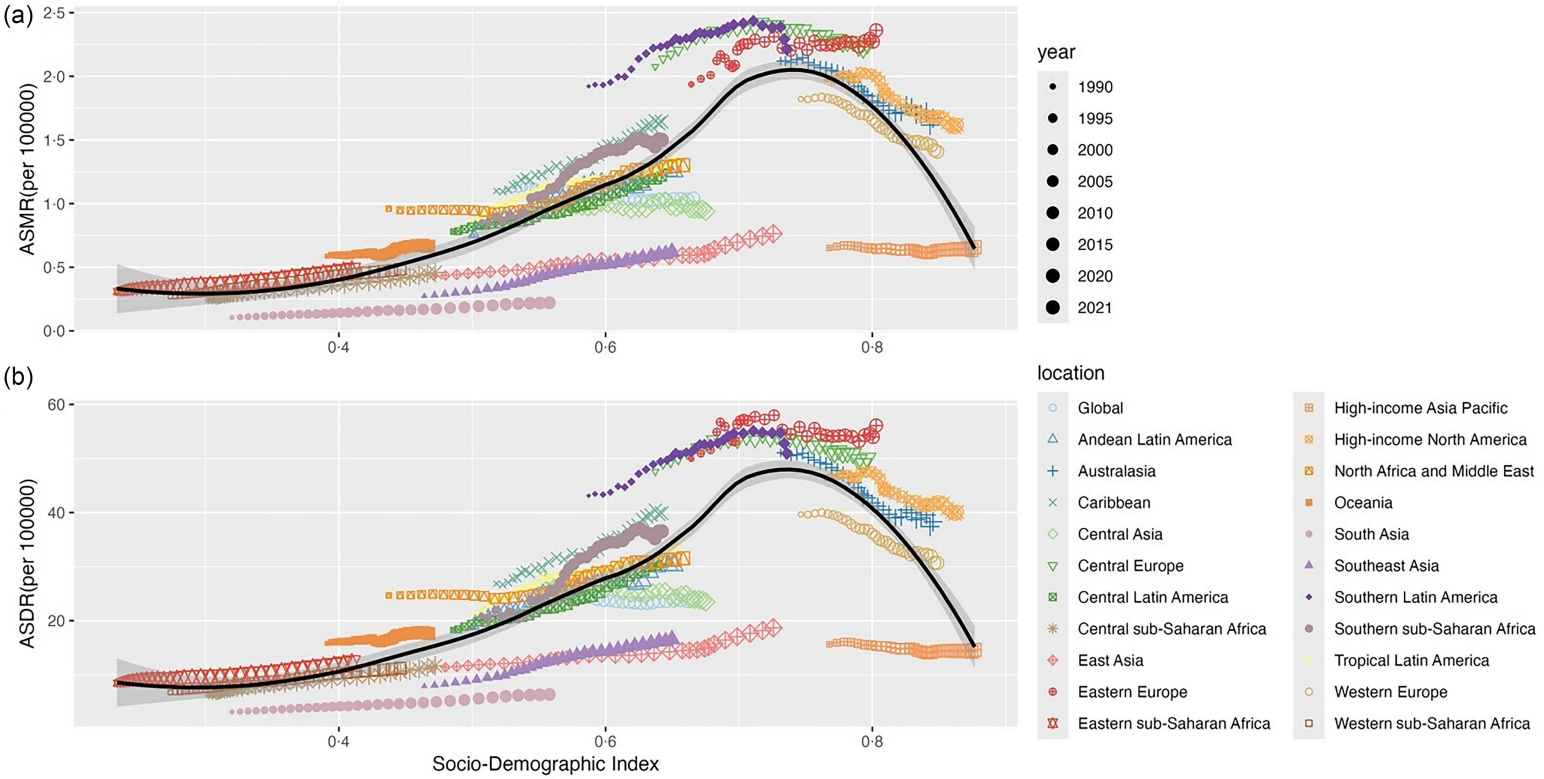
The associations between ASR and SDI for female colon and rectum cancer attributable to high BMI across twenty-one regions from 1990 to 2021. The black line represents the average expected relationship between SDI and ASR based on data for all regions from 1990 to 2021. The grey shaded area indicates the 95 % CI expected value. Each of the twenty-one regions is represented by a different colour and shape, with each dot representing a year’s disease burden in that region. Each region is plotted with thirty-two points, ranging from small to large, showing ASMR and ASDR observed each year from 1990 to 2021. Points above the solid line indicate that the burden is higher than expected, and points below the solid line indicate that the burden is lower than expected. ASR includes ASMR and ASDR. ASMR, age-standardised mortality rate; ASDR, age-standardised DALY rate; DALY, disability-adjusted life years; SDI, socio-demographic index.

**Fig. 8.**
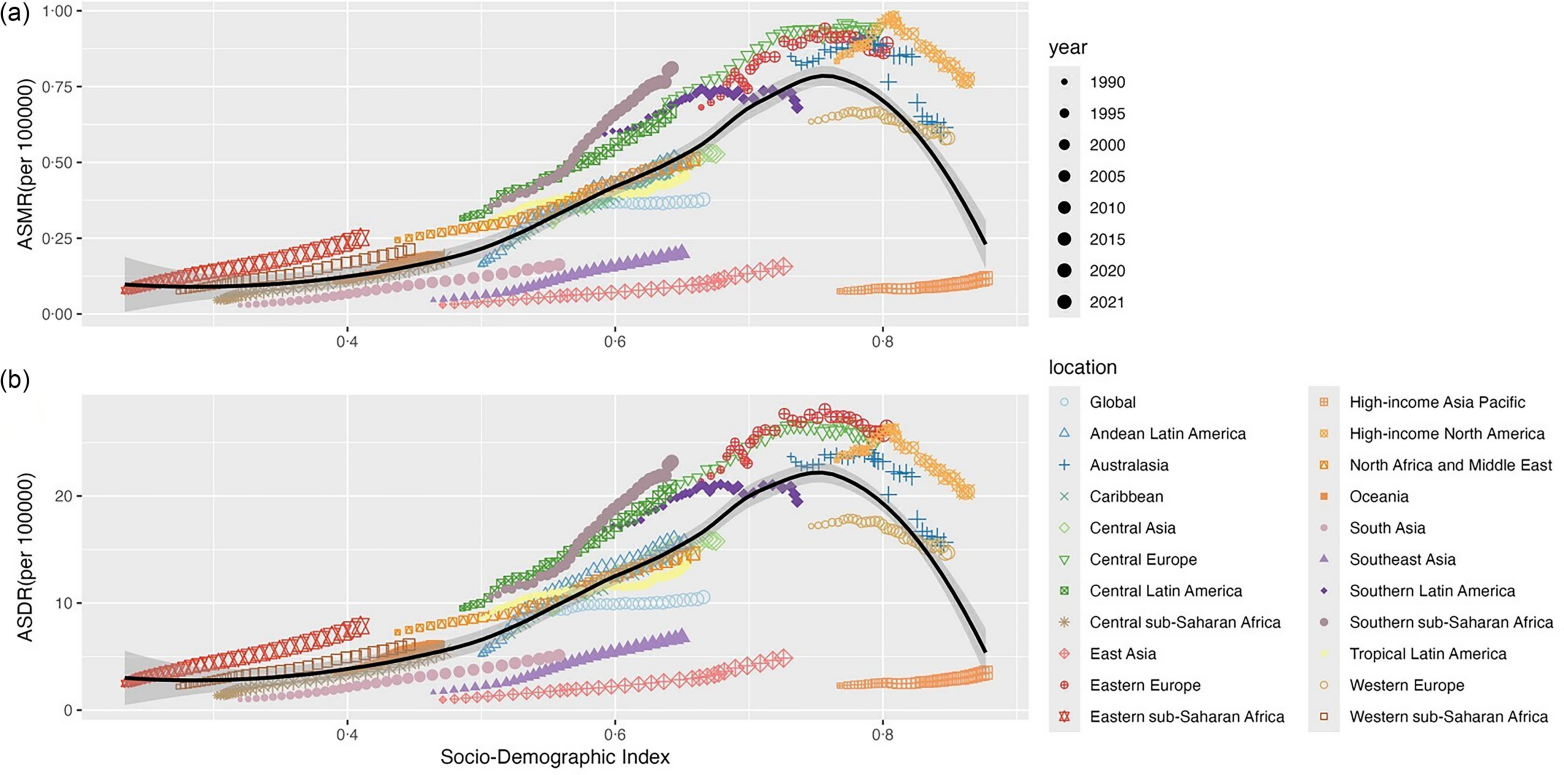
The associations between ASR and SDI for ovarian cancer attributable to high BMI across twenty-one regions from 1990 to 2021. The black line represents the average expected relationship between SDI and ASR based on data for all regions from 1990 to 2021. The grey shaded area indicates the 95 % CI expected value. Each of the twenty-one regions is represented by a different colour and shape, with each dot representing a year’s disease burden in that region. Each region is plotted with 32 points, ranging from small to large, showing ASMR and ASDR observed each year from 1990 to 2021. Points above the solid line indicate that the burden is higher than expected, and points below the solid line indicate that the burden is lower than expected. ASR includes ASMR and ASDR. ASMR, age-standardised mortality rate; ASDR, age-standardised DALY rate; DALY, disability-adjusted life years; SDI, socio-demographic index.

**Fig. 9.**
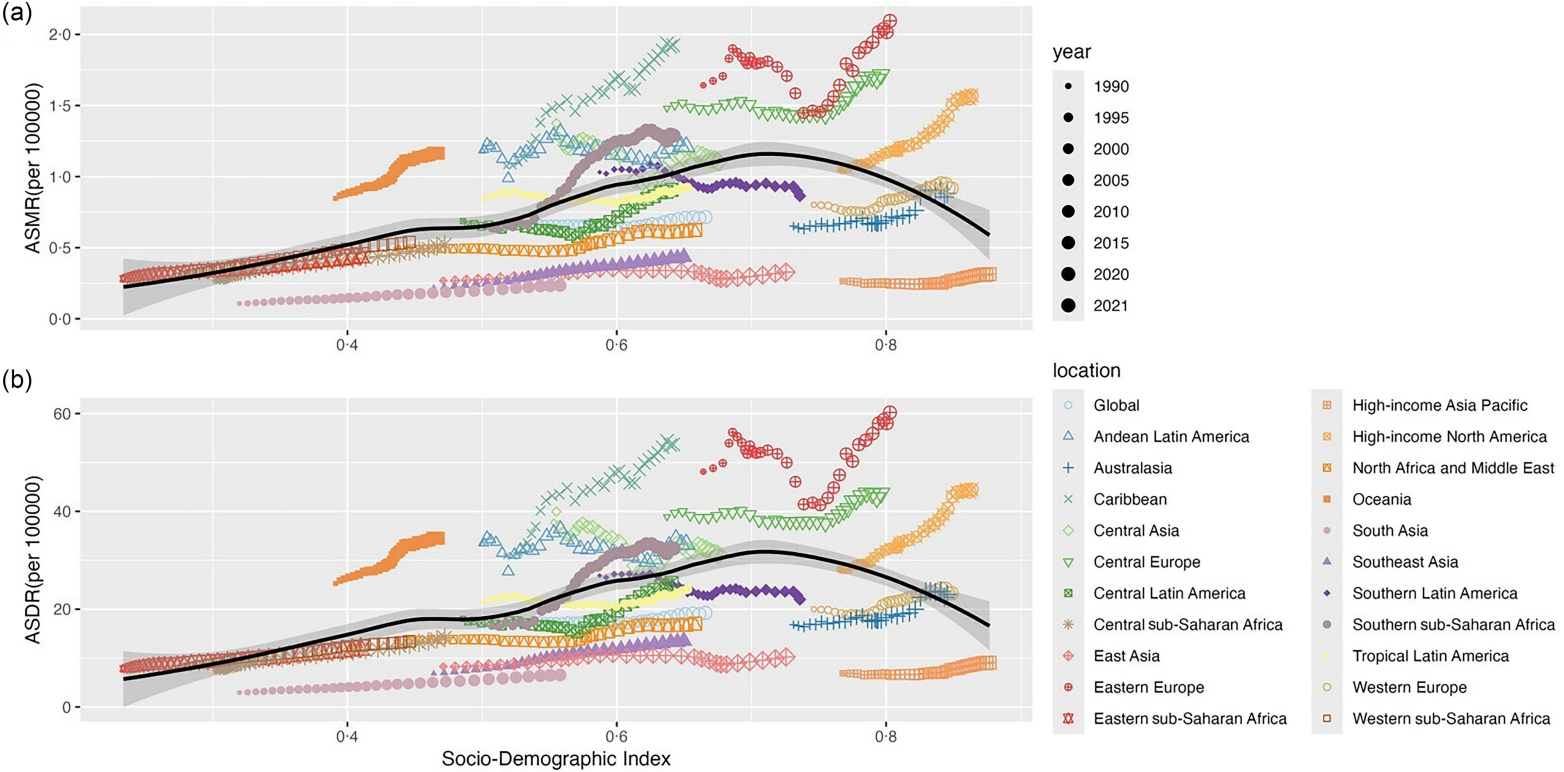
The associations between ASR and SDI for uterine cancer attributable to high BMI across twenty-one regions from 1990 to 2021. The black line represents the average expected relationship between SDI and ASR based on data for all regions from 1990 to 2021. The grey shaded area indicates the 95 % CI expected value. Each of the twenty-one regions is represented by a different colour and shape, with each dot representing a year’s disease burden in that region. Each region is plotted with thirty-two points, ranging from small to large, showing ASMR and ASDR observed each year from 1990 to 2021. Points above the solid line indicate that the burden is higher than expected, and points below the solid line indicate that the burden is lower than expected. ASR includes ASMR and ASDR. ASMR, age-standardised mortality rate; ASDR, age-standardised DALY rate; DALY, disability-adjusted life years; SDI, socio-demographic index.

From 1990 to 2021, the ASMR and ASDR for all three cancers attributable to high BMI initially increased and then decreased with rising SDI, peaking in regions classified as high-middle SDI. Across the twenty-one regions, increases in ASMR and ASDR for all three cancers were mainly concentrated in low-middle-income regions, but most high-income regions exceeded expected levels in all years (Figs. 7–9).

### The age-standardised mortality rate and age-standardised disability-adjusted life years rate global trends of three high BMI-attributed female pelvic cancers predicted by the Bayesian Age-Period-Cohort model

**Fig. 10.**
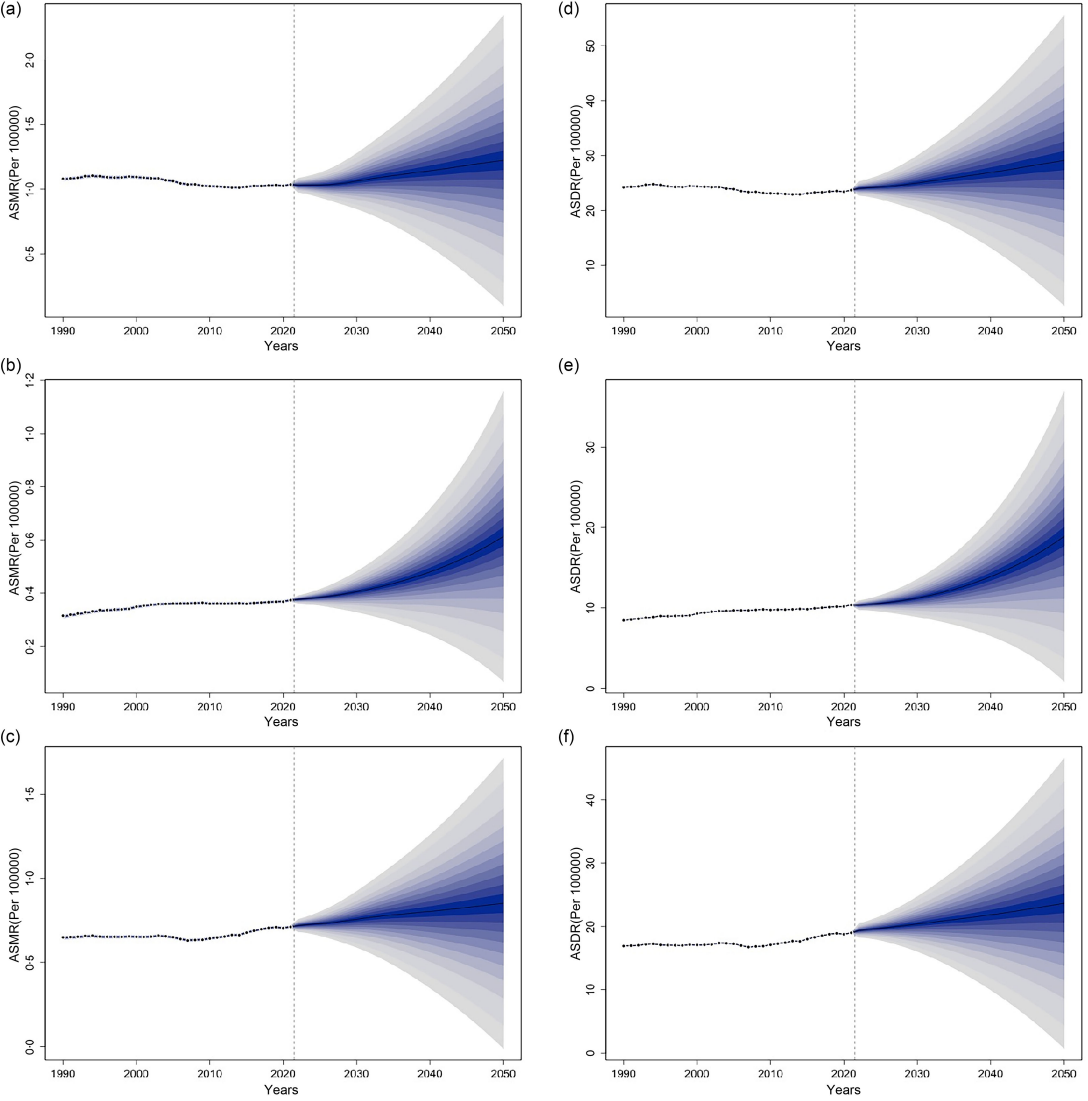
Time trends in ASMR and ASDR for high BMI-attributed female colon and rectum cancer (a) and (d), ovarian cancer (b) and (e) and uterine cancer (c) and (f) from 1990 to 2050. The vertical dashed line represents the starting point of the forecast, the forecast average is shown as a solid line, and the fan represents the 95 % UI. ASMR, age-standardised mortality rate; ASDR, age-standardised DALY rate; DALY, disability-adjusted life years; UI, uncertainty interval.

From 2022 to 2050, the ASMR and ASDR for these three cancers attributable to high BMI are expected to increase globally. Ovarian cancer is expected to rise the most, with the ASMR and ASDR expected to rise 63·03 and 83·03 %. In contrast, colon and rectum and uterine cancers showed similar increases in the ASMR and ASDR (Fig. 10).

## Discussion

Previous studies have separately assessed the burden of female colon and rectal, ovarian and uterine cancers, identifying high BMI as a common risk factor^(11,23–26)^. However, few studies have comprehensively assessed the combined impact of high BMI on these three cancers in women, limiting our understanding of the influence of BMI. The study is the first to comprehensively analyse the burden of high BMI on these three cancers. The results showed that 30·99 % of all three cancer deaths globally were linked to high BMI, and there was some commonality in the disease burden across regions and age differences, consistent with the study hypothesis. From the pathological mechanism analysis, high BMI primarily influences tumour occurrence and progression through pathways promoting chronic inflammation, metabolic alterations and hormonal changes^(27,28)^.

Globally, deaths and DALY for these three cancers attributable to high BMI increased in 2021 compared to 1990, with female colon and rectal cancer showing higher death counts, DALY and ASR than ovarian and uterine cancers, indicating a greater vulnerability to high BMI. From 1990 to 2021, the ASMR and ASDR for ovarian and uterine cancers rose, with ovarian cancer showing the largest increase. Despite high BMI’s significant impact on female colon and rectal cancer, both ASMR and ASDR have decreased over the past 32 years, likely due to increased colonoscopy screenings and advancements in tumour treatment technology^(29,30)^, reflecting successful obesity prevention efforts^(31)^. However, the challenge of high BMI persists, particularly for ovarian and uterine cancers, necessitating stronger prevention strategies to reduce BMI and control further progression to cancer.

At the regional level, the burden of female colon and rectal, ovarian and uterine cancers attributable to high BMI was highest in high- and high-middle SDI regions, such as East Asia, Western Europe, Eastern Europe, Central Europe and High-income North America. It may be that higher economic levels in these regions have led to unhealthy dietary patterns, such as excessive consumption of processed food and red meat. This is associated with increased BMI and cancer risk^(32)^. So public health campaigns and policy interventions are critical to address these dietary risks. The difference is that across the country, the United Arab Emirates reported the highest ASMR and ASDR for these three cancers attributable to high BMI. This phenomenon correlates with the high obesity rate in the United Arab Emirates, where up to 75·8 % of the female population is classified as overweight or obese^(33)^. Rapid economic growth in the United Arab Emirates has resulted in unhealthy eating habits, unbalanced nutrition and reduced physical activity^(34)^, leading to a cancer burden exceeding that in more developed regions.

While the burden was highest in high or high-middle SDI regions, the fastest increases in ASMR and ASDR from 1990 to 2021 occurred in low-middle SDI regions, particularly in Southeast Asia, South Asia, Central Asia and Southern Sub-Saharan Africa. Conversely, significant declines were observed in economically mature regions, aligning with previous findings^(13,35)^. This highlights a complex interaction between cancer burden and various regional factors, including race, ethnicity, geography and education^(36)^. Rapid socio-economic development often promotes unhealthy lifestyles, which is thought to be one of the main causes of elevated BMI. In emerging economic zones, inadequate cancer prevention and treatment resources contributed to higher cancer mortality and DALY. In contrast, high-income regions focus on obesity prevention and have sufficient financial resources to implement healthy food initiatives, weight loss programmes and cancer screening to reduce cancer risk caused by high BMI^(37)^. Many Western countries, including the USA, have successfully reduced obesity rates through health education, promoting healthy diets, lowering prices of healthy foods, increasing taxes on unhealthy foods and restricting unhealthy food advertising^(38)^. These efforts have been extended to schools and implemented from childhood onwards^(39)^. As women’s status and education levels improve, these countries focus more on preventing obesity in females^(40)^. Countries like the USA, the UK and Italy have introduced targeted screening for early detection of these female cancers, reducing mortality^(41–43)^. These initiatives enhance public health awareness and serve as valuable models for other nations.

In 2021, the highest number and rate of deaths and DALY attributable to high BMI for female colon and rectal cancer were predominantly observed in individuals over 80 years old, likely due to treatment challenges as health declines in older adults. For ovarian and uterine cancers, peak deaths and DALY were in the 55–59 age group, associated with postmenopausal hormonal changes that can lead to metabolic syndrome and abdominal obesity^(44)^. From 1990 to 2021, a general downward trend with age was seen in the burden of these cancers linked to high BMI. The increase was most significant in the 20–24 and 25–29 age groups, suggesting a shift towards younger demographics, with previous research supporting this observation^(13,45,46)^. This trend is likely related to poor dietary habits, smoking, sedentary lifestyles and work-related stress, all contributing to higher BMI^(47)^.

Our study also showed that the adverse effects of high BMI on these three cancers will continue to increase over the next nearly 30 years, with ovarian cancer being particularly prominent, which poses a huge challenge to global cancer prevention and treatment efforts.

Based on the above series of research results, we urgently need to take more active, effective and targeted measures based on existing measures to reduce the adverse impact of high BMI on these three cancers and control the occurrence and development of cancer. In 2018, Cancer Research UK initiated a campaign to raise awareness of the link between obesity and cancer^(48)^. Increasingly, countries are promoting plant-based diets, believed to mitigate obesity and cancer risk^(49)^. The 2020–2025 Dietary Guidelines for Americans recommend a ‘Healthy Vegetarian Dietary Pattern’^(50)^. Consequently, plant-based diets represent a promising avenue for future research in controlling BMI and reducing cancer risk. The burden of these cancers associated with high BMI varies across regions due to factors, including disease characteristics, social environment, cultural differences and economic status. Thus, countries must develop and adjust targeted prevention and treatment strategies based on their specific circumstances. The World Cancer Research Fund and the American Institute for Cancer Research have jointly published the Global Report on Diet, Nutrition, Physical Activity and Cancer, offering a practical framework for countries^(51)^. Current research mainly focuses on high-income nations, so low- and middle-income countries, along with diverse genetic and ethnic populations, should be included in future studies. In low and low-middle SDI areas, enhancing awareness of high BMI health risks is crucial as economic development progresses. These regions can learn from developed nations by promoting healthy eating, reducing healthy food costs and implementing affordable early cancer screening methods like gynaecologic ultrasound and colonoscopy. Conversely, in economically thriving regions with robust cancer screening and treatment, high obesity rates remain a challenge, necessitating continued focus on weight management. Furthermore, the burden of high BMI on these three cancers was trending younger. It’s essential to prioritise health management for young women, who face heightened cancer risks due to balancing home and work responsibilities. Governments should prioritise health management for young women and allocate resources effectively to support their weight control and cancer prevention initiatives. Through these comprehensive measures, we aim to alleviate the adverse effects of high BMI on women’s health and contribute positively to overall health.

This study has some limitations. The data were from the GBD database, and although reviewed by expert teams, data accuracy may be compromised in areas with limited medical resources and underdeveloped economies. Additionally, it should be pointed out that the GBD dataset uses a binary BMI ≥ 25 kg/m^2^ threshold and does not account for visceral *v*. subcutaneous fat distribution, which may influence cancer risk mechanisms. This limitation affects the interpretability of BMI’s specific impact on pelvic cancers.

### Conclusions

The study revealed the complex epidemiology of high BMI for the three female pelvic cancers. In 2021, the global disease burden of colon and rectal cancer was particularly prominent, while the burden of all three cancers was also high in high-income regions. However, over the past 32 years, the burden of ovarian and uterine cancers has been on the rise, and the increase in these three cancers was particularly significant in low-income regions and younger age groups. The burden of the three cancers associated with high BMI is expected to continue to increase through 2050. Therefore, countries and regions need to implement targeted obesity prevention and control measures focused on diet, nutrition and activity to mitigate the effects of high BMI on these cancers.

## Supporting information

Jiang et al. supplementary material Jiang et al. supplementary material

## Supplementary material

For supplementary material accompanying this paper, visit https://doi.org/10.1017/S0007114525105035.
