# Supplementary material for: The global, regional and national burden of three female pelvic cancers attributable to high BMI from 1990 to 2021: a systematic analysis for the Global Burden of Disease Study 2021 and projection to 2050
Source: Br J Nutr. 2025 Sep 3;134(5):364–76. doi: 10.1017/S0007114525105035 (PMC12580978; doi:10.1017/S0007114525105035)
Supplement: Jiang et al. supplementary material [file S0007114525105035sup001.doc]

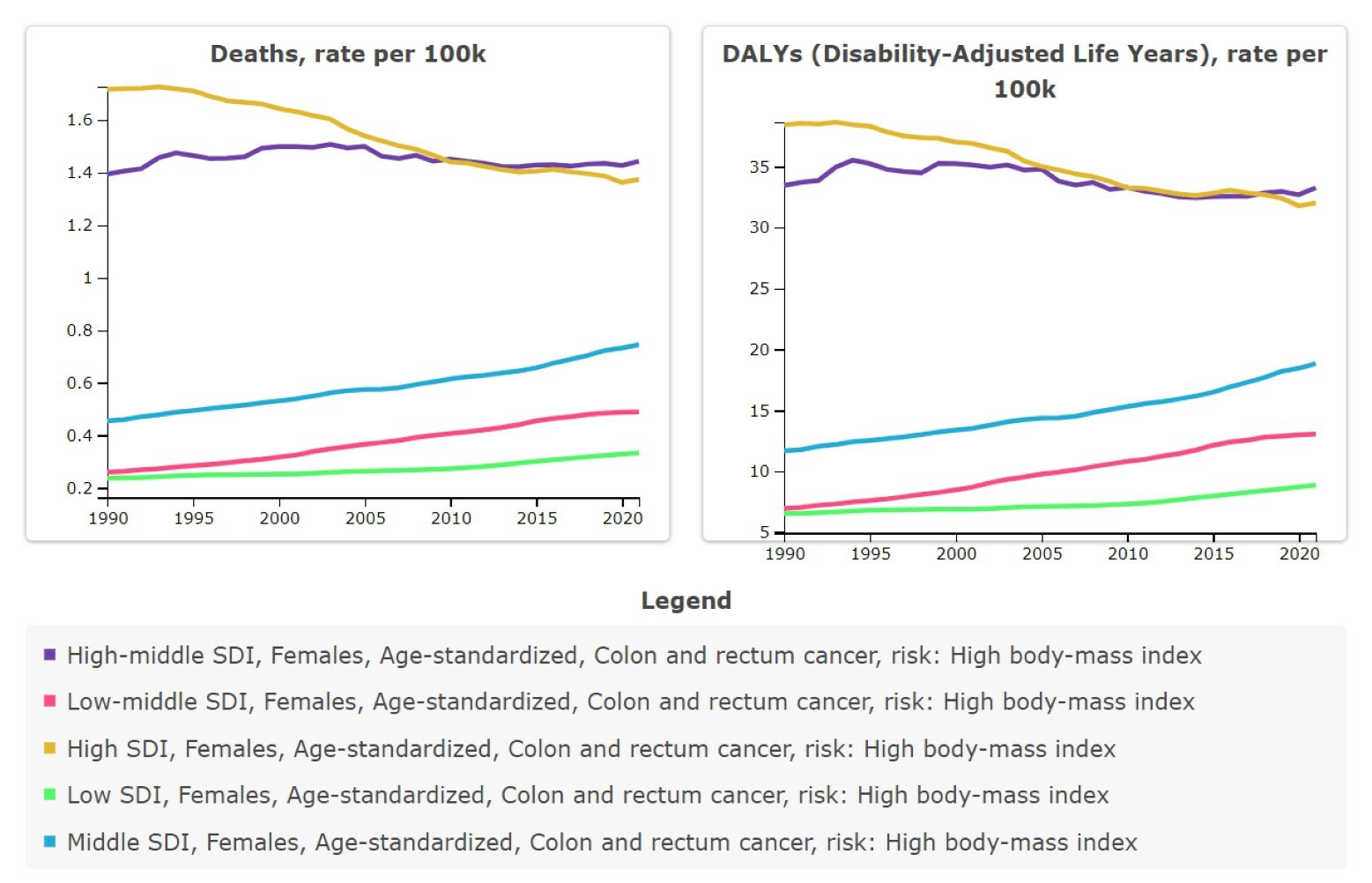


**Fig. S1** Temporal trends in ASMR and ASDR for female colon and rectum cancer attributable to high BMI from 1990 to 2021, in five SDI. Graphic source: GBD study 2021, VizHub - GBD Results [Internet]. [accessed 29 July 2024]. (Available from: http://ghdx.healthdata.org/gbd-results-tool). ASMR, age-standardized mortality rate; ASDR, age-standardized DALY rate; DALY, disability-adjusted life years; BMI, body mass index; SDI, socio-demographic index.


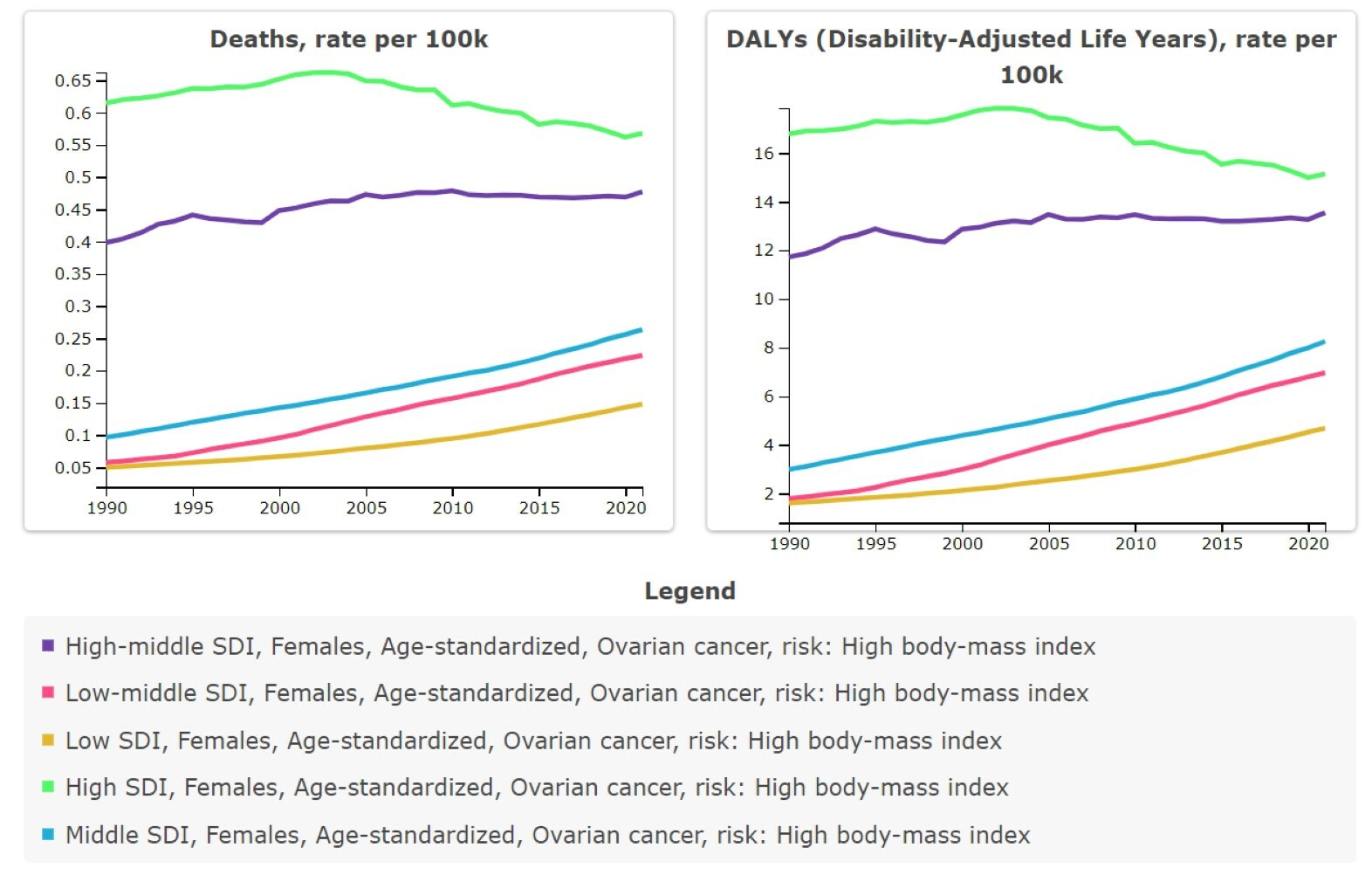


**Fig. S2** Temporal trends in ASMR and ASDR for ovarian cancer attributable to high BMI from 1990 to 2021, in five SDI. Both the ASMR and the ASDR decreased in high SDI regions. Graphic source: GBD study 2021, VizHub - GBD Results [Internet]. [accessed 29 July 2024]. (Available from: http://ghdx.healthdata.org/gbd-results-tool). ASMR, age-standardized mortality rate; ASDR, age-standardized DALY rate; DALY, disability-adjusted life years; BMI, body mass index; SDI, socio-demographic index.


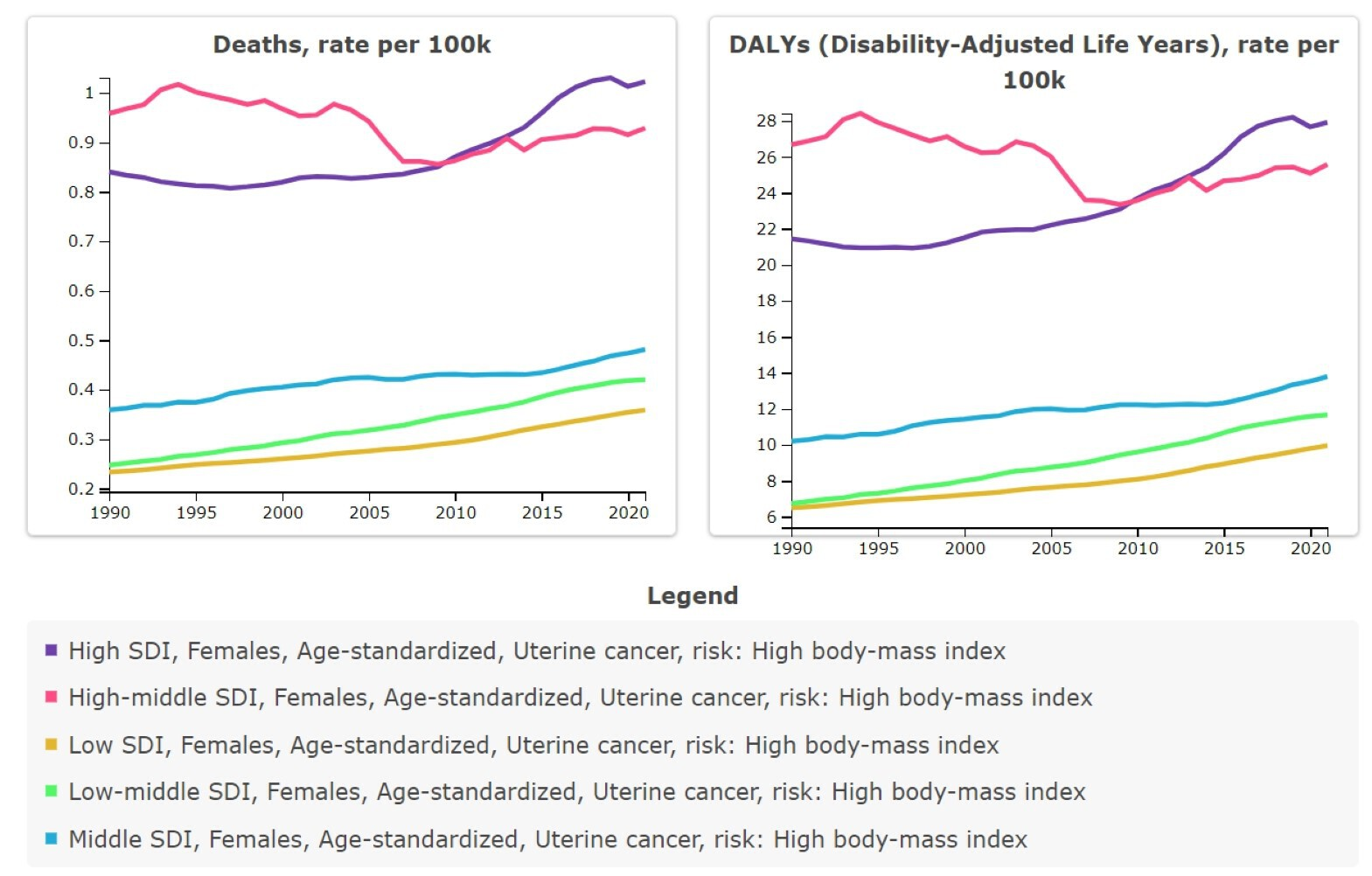


**Fig. S3** Temporal trends in ASMR and ASDR for uterine cancer attributable to high BMI from 1990 to 2021, in five SDI. Both the ASMR and the ASDR decreased in high-middle SDI regions. Graphic source: GBD study 2021, VizHub - GBD Results [Internet]. [accessed 29 July 2024]. (Available from: http://ghdx.healthdata.org/gbd-results-tool). ASMR, age-standardized mortality rate; ASDR, age-standardized DALY rate; DALY, disability-adjusted life years; BMI, body mass index; SDI, socio-demographic index.

**Table S1** The numbers of all-cause and high BMI deaths of female colon and rectum, ovarian, and uterine cancer in 2021

| **Type** | **Deaths** |
| --- | --- |
| Total of female pelvic cancers | 318,732 |
| High BMI-associated female pelvic cancers | 98,770 |

BMI, body mass index.

**Table S2** Deaths and DALYs for female colon and rectum cancer attributable to high BMI in 1990 and 2021, with corresponding EAPC from 1990 to 2021, in five SDI and 21 regions

|  | **Deaths** | | | | | **DALYs** | | | | |
| --- | --- | --- | --- | --- | --- | --- | --- | --- | --- | --- |
| **Location** | **Number of cases**  **(95%CI),**  **1990** | **ASMR**  **per 100,000**  **(95%CI),**  **1990** | **Number of cases**  **(95%CI),**  **2021** | **ASMR per 100,000**  **(95%CI),**  **2021** | **EAPC**  **of ASMR**  **(95%CI),**  **1990-2021** | **Number of**  **cases**  **(95%CI),**  **1990** | **ASDR**  **per 100,000**  **(95%CI),**  **1990** | **Number of**  **cases**  **(95%CI),**  **2021** | **ASDR**  **per 100,000**  **(95%CI),**  **2021** | **EAPC**  **of ASDR**  **(95%CI),**  **1990-2021** |
| **SDI** |  |  |  |  |  |  |  |  |  |  |
| Middle SDI | 2317  (881,3805) | 0.46  (0.17,0.75) | 10392  (4534,16672) | 0.75  (0.32,1.20) | 1.54  (1.50,1.57) | 66507  (25376,109294) | 11.65  (4.43,19.13) | 271358  (119587,432812) | 18.83  (8.28,30.06) | 1.48  (1.43,1.54) |
| High SDI | 11466  (4895,18702) | 1.72  (0.73,2.79) | 17248  (7350,27781) | 1.37  (0.60,2.19) | -0.88  (-0.94,-0.82) | 238746  (101776,384587) | 38.41  (16.36,61.62) | 337275  (147761,539197) | 32.01  (14.16,50.64) | -0.70  (-0.75,-0.65) |
| High-middle SDI | 7729  (3356,12450) | 1.39  (0.60,2.25) | 16090  (6939,25359) | 1.44  (0.62,2.28) | -0.05  (-0.13,0.03) | 188097  (81335,302353) | 33.44  (14.43,53.87) | 354770  (153778,560241) | 33.25  (14.42,52.68) | -0.24  (-0.32,-0.16) |
| Low-middle SDI | 768  (291,1238) | 0.26  (0.10,0.42) | 3628  (1550,5685) | 0.49  (0.21,0.77) | 2.25  (2.19,2.31) | 23293  (8855,37431) | 6.94  (2.64,11.19) | 104563  (44723,163283) | 13.05  (5.58,20.41) | 2.23  (2.17,2.29) |
| Low SDI | 266  (98,460) | 0.23  (0.09,0.41) | 859  (345,1371) | 0.33  (0.13,0.53) | 1.05  (0.95,1.15) | 8202  (3048,14176) | 6.51  (2.41,11.25) | 26196  (10611,42076) | 8.84  (3.56,14.11) | 0.90  (0.79,1.00) |
| **GBD regions** |  |  |  |  |  |  |  |  |  |  |
| Oceania | 8  (3,14) | 0.59  (0.23,1.00) | 24  (10,39) | 0.66  (0.28,1.07) | 0.46  (0.38,0.54) | 263  (107,459) | 15.79  (6.4,27.2) | 750  (312,1215) | 17.63  (7.35,28.48) | 0.38  (0.32,0.45) |
| East Asia | 1848  (644,3151) | 0.43  (0.15,0.74) | 8689  (3469,15102) | 0.77  (0.31,1.33) | 1.68  (1.58,1.79) | 53301  (18214,91517) | 11.25  (3.86,9.27) | 212010  (84682,368477) | 18.69  (7.44,32.46) | 1.44  (1.32,1.57) |
| Southeast Asia | 384  (136,634) | 0.27  (0.09,0.45) | 2171  (886,3595) | 0.62  (0.25,1.02) | 2.72  (2.60,2.84) | 12209  (4410,20208) | 7.88  (2.82,13.06) | 61798  (25533,102203) | 16.55  (6.81,27.23) | 2.47  (2.34,2.61) |
| Central Asia | 297  (123,474) | 1.07  (0.45,1.71) | 421  (183,663) | 0.94  (0.41,1.49) | -0.15  (-0.25,-0.05) | 8038  (3346,12742) | 28.7  (11.95,45.49) | 11218  (4831,17811) | 23.48  (10.11,37.1) | -0.49  (-0.55,-0.43) |
| Eastern Europe | 3550  (1542,5689) | 1.94  (0.84,3.10) | 5374  (2338,8338) | 2.36  (1.03,3.68) | 0.40  (0.28,0.51) | 88012  (38114,141440) | 49.94  (21.64,80.09) | 118493  (52135,185174) | 56.09  (24.61,87.95) | 0.05  (-0.08,0.19) |
| Central Europe | 1784  (786,2848) | 2.08  (0.92,3.32) | 3081  (1374,4970) | 2.26  (1.01,3.63) | 0.05  (-0.09,0.19) | 40553  (17787,65104) | 47.56  (20.89,76.3) | 61253  (27760,98661) | 50.04  (22.74,80.74) | -0.01  (-0.14,0.12) |
| Australasia | 278  (116,437) | 2.12  (0.89,3.34) | 516  (219,840) | 1.67  (0.71,2.69) | -0.92  (-0.99,-0.85) | 6324  (2603,9888) | 51.04  (20.9,79.95) | 10251  (4420,16477) | 38.29  (16.44,61.27) | -1.09  (-1.17,-1.02) |
| High-income  Asia Pacific | 734  (275,1189) | 0.65  (0.24,1.06) | 2127  (815,3483) | 0.66  (0.26,1.06) | -0.11  (-0.19,-0.03) | 17588  (6537,28434) | 15.62  (5.79,25.29) | 35341  (13837,57151) | 14.62  (5.81,23.38) | -0.37  (-0.45,-0.29) |
| Western Europe | 6605  (2815,10741) | 1.82  (0.78,2.96) | 8431  (3528,14010) | 1.41  (0.60,2.32) | -0.93  (-0.98,-0.88) | 130080  (55397,210842) | 39.64  (16.85,64.17) | 149860  (64070,247075) | 30.68  (13.12,50.19) | -0.91  (-0.96,-0.86) |
| Southern  Latin America | 490  (210,794) | 1.92  (0.82,3.12) | 1131  (503,1849) | 2.21  (0.99,3.60) | 0.68  (0.53,0.83) | 11020  (4777,17914) | 43.12  (18.65,70.06) | 24045  (10859,38649) | 50.95  (23.09,82.21) | 0.78  (0.66,0.9) |
| High-income  North America | 4025  (1790,6817) | 1.98  (0.85,3.19) | 5973  (2624,9344) | 1.61  (0.72,2.52) | -0.84  (-0.95,-0.74) | 89626  (38694,143225) | 46.38  (20.11,73.53) | 129328  (58805,198642) | 40.1  (18.41,61.54) | -0.6  (-0.68,-0.51) |
| Andean  Latin America | 77  (32,130) | 0.75  (0.31,1.26) | 385  (163,652) | 1.25  (0.53,2.12) | 1.63  (1.48,1.78) | 2090  (869,3533) | 18.73  (7.77,31.64) | 9478  (4122,15895) | 30.16  (13.12,50.63) | 1.46  (1.32,1.6) |
| Caribbean | 144 (61,229) | 1.10  (0.46,1.75) | 480  (205,782) | 1.64  (0.70,2.68) | 1.40  (1.36,1.44) | 3658  (1551,5852) | 26.85  (11.37,43) | 11317  (4929,18473) | 39.89  (17.37,65.16) | 1.4  (1.35,1.45) |
| Central  Latin America | 317  (133,509) | 0.78  (0.32,1.25) | 1656  (747,2665) | 1.23  (0.55,1.98) | 1.49  (1.41,1.57) | 8353  (3550,13384) | 18.26  (7.74,29.26) | 42902  (19358,68571) | 31.12  (14.03,49.75) | 1.73  (1.64,1.82) |
| Tropical Latin America | 407  (167,649) | 0.87  (0.36,1.39) | 1907  (800,3058) | 1.34  (0.56,2.15) | 1.31  (1.19,1.42) | 10903  (4568,17416) | 21.28  (8.87,33.84) | 48381  (20508,77444) | 34.22  (14.49,54.74) | 1.4  (1.3,1.5) |
| North Africa and Middle East | 768  (317,1244) | 0.96  (0.40,1.58) | 2750  (1230,4311) | 1.30  (0.59,2.05) | 1.24  (1.05,1.43) | 22591  (9202,36900) | 24.69  (10.15,40.09) | 75913  (33253,118988) | 31.55  (13.89,49.49) | 0.95  (0.79,1.11) |
| South Asia | 308  (105,517) | 0.11  (0.04,0.18) | 1698  (683,2740) | 0.22  (0.09,0.35) | 2.44  (2.36,2.51) | 10252  (3494,17107) | 3.14  (1.07,5.27) | 51936  (20561,82617) | 6.35  (2.52,10.15) | 2.32  (2.23,2.4) |
| Central Sub-Saharan Africa | 28  (11,49) | 0.24  (0.10,0.42) | 132  (50,229) | 0.46  (0.17,0.81) | 2.08  (1.98,2.19) | 833  (328,1448) | 6.23  (2.51,10.72) | 3909  (1497,6658) | 11.65  (4.41,20.2) | 2.06  (1.97,2.16) |
| Eastern  Sub-Saharan Africa | 117  (45,199) | 0.31  (0.12,0.52) | 408  (160,679) | 0.47  (0.19,0.78) | 1.28  (1,19,1.37) | 3628  (1412,6222) | 8.45  (3.29,14.44) | 12190  (4696,20127) | 12.08  (4.72,20.08) | 1.01  (0.91,1.11) |
| Western Sub-Saharan Africa | 112  (45,180) | 0.27  (0.11,0.44) | 458  (183,756) | 0.48  (0.20,0.79) | 1.99  (1.95,2.04) | 3046  (1238,4933) | 6.73  (2.72,10.9) | 12662  (4837,20974) | 11.22  (4.43,18.55) | 1.75  (1.71,1.8) |
| Southern Sub-Saharan Africa | 125  (53,207) | 0.85  (0.36,1.42) | 480  (206,751) | 1.50  (0.65,2.35) | 2.11  (1.89,2.33) | 3411  (1456,5470) | 20.9  (8.9,34.01) | 12737  (5473,19750) | 36.51  (15.68,56.7) | 2.25  (2.02,2.48) |

BMI, body mass index; DALYs, disability-adjusted life years; ASMR, age-standardized mortality rate; ASDR, age-standardized DALY rate; EAPC, estimated annual percentage change; UI,

uncertainty interval; CI, confidence interval; SDI, socio-demographic index.

**Table S3** Deaths and DALYs for ovarian cancer attributable to high BMI in 1990 and 2021, with corresponding EAPC from 1990 to 2021, in five SDI and 21 regions

|  | **Deaths** | | | | | **DALYs** | | | | |
| --- | --- | --- | --- | --- | --- | --- | --- | --- | --- | --- |
| **Location** | **Number of cases**  **(95%UI),**  **1990** | **ASMR per 100,000 (95%UI),**  **1990** | **Number of cases (95%UI),**  **2021** | **ASMR per 100,000 (95%UI),**  **2021** | **EAPC of ASMR (95%CI),**  **1990-2021** | **Number of**  **Cases**  **(95%UI),**  **1990** | **ASDR per 100,000 (95%UI),**  **1990** | **Number of**  **cases**  **(95%UI),**  **2021** | **ASDR per 100,000**  **(95%UI),**  **2021** | **EAPC of**  **ASDR (95%UC),**  **1990-2021** |
| **SDI** |  |  |  |  |  |  |  |  |  |  |
| Middle SDI | 543  (65,1064) | 0.1  (0.01,0.19) | 3833  (904,6972) | 0.26  (0.06,0.48) | 3.16  (3.09,3.24) | 17976  (2083,35888) | 2.99  (0.36,5.9) | 121139  (28727,221614) | 8.25  (1.95,15.09) | 3.2  (3.13,3.26) |
| High SDI | 3802  (812,7120) | 0.61  (0.13,1.15) | 6187  (1531,10979) | 0.57  (0.14,1.01) | -0.36  (-0.49,-0.23) | 96742  (20587,180828) | 16.78  (3.57,31.36) | 144449  (36080,255583) | 15.13  (3.79,26.82) | -0.43  (-0.56,-0.3) |
| High-middle SDI | 2244  (484,4207) | 0.4  (0.09,0.75) | 5095  (1241,9060) | 0.48  (0.12,0.85) | 0.47  (0.37,0.58) | 65389  (13956,121986) | 11.71  (2.49,21.84) | 138126  (33461,244871) | 13.54  (3.26,24.13) | 0.33  (0.25,0.42) |
| Low-middle SDI | 187  (13,392) | 0.06  (0,0.12) | 1771  (357,3220) | 0.22  (0.04,0.41) | 4.69  (4.49,4.89) | 6283  (459,13117) | 1.78  (0.13,3.74) | 57906  (11973,104671) | 6.96  (1.43,12.58) | 4.69  (4.49,4.89) |
| Low SDI | 62  (0,139) | 0.05  (0,0.11) | 433  (68,840) | 0.15  (0.02,0.29) | 3.61  (3.52,3.7) | 2127  (24,4781) | 1.6  (0.01,3.56) | 14943  (2349,28748) | 4.68  (0.74,9.04) | 3.55  (3.45,3.65) |
| **GBD regions** |  |  |  |  |  |  |  |  |  |  |
| Oceania | 2  (0,4) | 0.11  (0.02,0.22) | 8  (2,15) | 0.19  (0.05,0.34) | 1.91  (1.76,2.06) | 62  (12,126) | 3.41  (0.66,6.95) | 283  (73,534) | 5.93  (1.54,11.06) | 1.84  (1.69,2) |
| East Asia | 145  (-70,392) | 0.03  (-0.02,0.08) | 1814  (356,3763) | 0.16  (0.03,0.33) | 5.04  (4.87,5.2) | 4731  (-2392,12911) | 0.97  (-0.47,2.63) | 54989  (10889,112924) | 4.86  (0.96,9.99) | 4.99  (4.84,5.13) |
| Southeast Asia | 73  (-5,160) | 0.05  (0,0.1) | 763  (142,1449) | 0.2  (0.04,0.38) | 4.96  (4.69,5.24) | 2759  (-83,6007) | 1.67  (-0.06,3.64) | 26640  (5007,50775) | 6.82  (1.27,12.97) | 4.74  (4.45,5.03) |
| Central Asia | 82  (18,151) | 0.29  (0.06,0.54) | 255  (62,459) | 0.53  (0.13,0.95) | 1.95  (1.79,2.11) | 2522  (554,4677) | 9.08  (2,16.79) | 7936  (1896,14300) | 15.77  (3.76,28.49) | 1.81  (1.64,1.97) |
| Eastern Europe | 1182  (280,2096) | 0.68  (0.16,1.21) | 1832  (491,3169) | 0.89  (0.24,1.54) | 0.81  (0.61,1) | 35233  (8318,62486) | 21.36  (4.99,37.8) | 50056  (13203,86238) | 26.48  (6.87,45.59) | 0.59  (0.4,0.78) |
| Central Europe | 595  (138,1095) | 0.7  (0.16,1.3) | 1138  (295,2060) | 0.94  (0.24,1.71) | 0.95  (0.76,1.13) | 16873  (3841,31014) | 20.59  (4.65,38.13) | 27713  (7172,50230) | 25.65  (6.53,46.23) | 0.72  (0.53,0.91) |
| Australasia | 107  (22,198) | 0.85  (0.18,1.58) | 173  (45,315) | 0.62  (0.16,1.1) | -0.96  (-1.34,-0.58) | 2816  (599,5214) | 23.68  (5,43.97) | 3935  (1041,7041) | 15.67  (4.12,27.86) | -1.25  (-1.62,-0.88) |
| High-income  Asia Pacific | 85  (-9,189) | 0.07  (-0.01,0.17) | 253  (22,508) | 0.12  (0.01,0.24) | 1.27  (1.15,1.39) | 2550  (-283,5765) | 2.29  (-0.28,5.19) | 6117  (662,12207) | 3.5  (0.38,6.99) | 1.21  (1.09,1.33) |
| Western Europe | 2051  (421,3876) | 0.63  (0.13,1.2) | 2895  (671,5329) | 0.58  (0.14,1.06) | -0.39  (-0.49,-0.3) | 50346  (10328,95161) | 17.18  (3.53,32.53) | 62381  (14564,114044) | 14.7  (3.42,26.9) | -0.62  (-0.72,-0.52) |
| Southern Latin  America | 152  (35,286) | 0.59  (0.14,1.12) | 320  (84,567) | 0.68  (0.18,1.2) | 0.65  (0.48,0.83) | 4241  (978,7970) | 16.81  (3.87,31.61) | 8627  (2305,15147) | 19.48  (5.21,34.22) | 0.67  (0.5,0.84) |
| High-income  North America | 1604  (382,2944) | 0.83  (0.2,1.52) | 2701  (726,4685) | 0.78  (0.21,1.34) | -0.35  (-0.59,-0.11) | 41346  (10085,74963) | 23.36  (5.74,42.27) | 64190  (17684,111223) | 20.45  (5.66,35.38) | -0.54  (-0.75,-0.32) |
| Andean Latin  America | 18  (3,38) | 0.16  (0.03,0.33) | 158  (39,305) | 0.5  (0.12,0.97) | 3.76  (3.35,4.18) | 620  (111,1285) | 5.2  (0.93,10.8) | 5018  (1245,9643) | 15.55  (3.86,29.85) | 3.68  (3.26,4.1) |
| Caribbean | 30  (6,57) | 0.22  (0.05,0.42) | 130  (31,238) | 0.46  (0.11,0.84) | 2.32  (2.11,2.53) | 951  (199,1788) | 6.81  (1.44,12.82) | 3937  (950,7222) | 14.23  (3.43,26.1) | 2.28  (2.07,2.5) |
| Central Latin  America | 145  (31,271) | 0.32  (0.07,0.59) | 924  (262,1669) | 0.67  (0.19,1.21) | 2.4  (2.3,2.49) | 4750  (1033,8901) | 9.51  (2.09,17.81) | 29277  (8418,52488) | 20.87  (5.99,37.44) | 2.52  (2.43,2.6) |
| Tropical Latin  America | 145  (30,282) | 0.28  (0.06,0.55) | 646  (150,1192) | 0.46  (0.11,0.84) | 1.24  (1.11,1.37) | 4626  (972,9022) | 8.52  (1.78,16.56) | 19032  (4476,34719) | 13.52  (3.18,24.66) | 1.18  (1.06,1.29) |
| North Africa and  Middle East | 215  (44,427) | 0.24  (0.05,0.49) | 1200  (345,2096) | 0.51  (0.15,0.89) | 2.53  (2.47,2.59) | 6994  (1428,13919) | 7.27  (1.49,14.33) | 37560  (10820,65762) | 14.62  (4.22,25.6) | 2.37  (2.31,2.42) |
| South Asia | 93  (-17,214) | 0.03  (-0.01,0.07) | 1303  (208,2414) | 0.16  (0.03,0.3) | 5.86  (5.7,6.02) | 3320  (-477,7497) | 0.98  (-0.16,2.23) | 42615  (6815,78757) | 5.11  (0.82,9.43) | 5.61  (5.49,5.74) |
| Central  Sub-Saharan Africa | 6  (0,14) | 0.05  (0,0.1) | 61  (10,125) | 0.18  (0.03,0.37) | 4.7  (4.53,4.87) | 195  (1,451) | 1.35  (0.01,3.08) | 2048  (345,4184) | 5.55  (0.92,11.34) | 4.73  (4.57,4.9) |
| Eastern  Sub-Saharan Africa | 33  (0,70) | 0.08  (0,0.17) | 253  (42,498) | 0.25  (0.04,0.49) | 3.84  (3.8,3.89) | 1133  (12,2412) | 2.48  (0.02,5.26) | 8753  (1483,17323) | 7.84  (1.32,15.4) | 3.74  (3.69,3.78) |
| Western  Sub-Saharan Africa | 33  (5,61) | 0.07  (0.01,0.14) | 239  (51,462) | 0.21  (0.05,0.41) | 3.44  (3.38,3.5) | 1031  (162,1905) | 2.18  (0.34,4.04) | 7736  (1633,14872) | 6.12  (1.3,11.8) | 3.34  (3.28,3.41) |
| Southern Sub-Saharan Africa | 56  (14,107) | 0.36  (0.09,0.68) | 278  (76,494) | 0.81  (0.22,1.43) | 2.89  (2.78,3) | 1774  (442,3343) | 10.58  (2.68,19.98) | 8404  (2318,14969) | 23.2(6.41,  41.25) | 2.79  (2.67,2.9) |

BMI, body mass index; DALYs, disability-adjusted life years; ASMR, age-standardized mortality rate; ASDR, age-standardized DALY rate; EAPC, estimated annual percentage change; UI, uncertainty interval; CI, confidence interval; SDI, socio-demographic index.

**Table S4** Deaths and DALYs for uterine cancer attributable to high BMI in 1990 and 2021, with corresponding EAPC from 1990 to 2021, in five SDI and 21 regions

|  | **Deaths** | | | | | **DALYs** | | | | |
| --- | --- | --- | --- | --- | --- | --- | --- | --- | --- | --- |
| **Location** | **Number of cases**  **(95%UI),**  **1990** | **ASMR per 100,000 (95%UI),**  **1990** | **Number of cases (95%UI),**  **2021** | **ASMR per 100,000 (95%UI),**  **2021** | **EAPC of ASMR (95%CI),**  **1990-2021** | **Number of**  **cases**  **(95%UI),**  **1990** | **ASDR per 100,000 (95%UI),**  **1990** | **Number of**  **cases**  **(95%UI),**  **2021** | **ASDR per 100,000 (95%UI),**  **2021** | **EAPC of ASDR (95%CI),**  **1990-2021** |
| **SDI** |  |  |  |  |  |  |  |  |  |  |
| Middle SDI | 1930  (1296,2641) | 0.36  (0.24,0.49) | 6899  (4839,9442) | 0.48  (0.34,0.66) | 0.81  (0.74,0.89) | 59298  (39261,81785) | 10.18  (6.8,14.03) | 202166  (141666,276177) | 13.78  (9.65,18.81) | 0.84  (0.77,0.91) |
| High SDI | 5501  (3912,7460) | 0.84  (0.6,1.14) | 11838  (8412,15583) | 1.02  (0.74,1.34) | 0.78  (0.62,0.94) | 131276  (94742,176287) | 21.45  (15.47,28.69) | 284156  (206127,368034) | 27.91  (20.5,36.15) | 1.05  (0.91,1.2) |
| High-middle SDI | 5428  (3844,7215) | 0.96  (0.68,1.28) | 10215  (7255,13456) | 0.93  (0.66,1.22) | -0.37  (-0.51,-0.23) | 151012  (106435,201013) | 26.67  (18.82,35.51) | 269988  (191569,355602) | 25.57  (18.19,33.72) | -0.44  (-0.59,-0.29) |
| Low-middle SDI | 740  (512,1005) | 0.25  (0.17,0.34) | 3174  (2190,4304) | 0.42  (0.29,0.57) | 1.79  (1.77,1.82) | 22215  (15282,30100) | 6.72  (4.63,9.14) | 93283  (63672,124564) | 11.67  (7.97,15.63) | 1.85  (1.83,1.88) |
| Low SDI | 264  (174,373) | 0.23  (0.15,0.33) | 947  (621,1365) | 0.36  (0.23,0.51) | 1.39  (1.32,1.45) | 8061  (5296,11370) | 6.49  (4.28,9.15) | 29007  (19141,42164) | 9.94  (6.52,14.4) | 1.35  (1.28,1.42) |
| **GBD regions** |  |  |  |  |  |  |  |  |  |  |
| Oceania | 13  (8,21) | 0.85  (0.5,1.33) | 46  (24,70) | 1.16  (0.62,1.74) | 1.1  (1.04,1.15) | 431  (252,670) | 25.26  (14.91,39.56) | 1508  (795,2398) | 34.48  (18.34,53.45) | 1.06  (1,1.11) |
| East Asia | 1247  (782,1874) | 0.27  (0.17,0.4) | 3778  (2322,5853) | 0.33  (0.2,0.51) | 0.32  (0.03,0.62) | 40485  (24801,60826) | 8.26  (5.08,12.38) | 115404  (69749,178922) | 10.23  (6.19,15.84) | 0.4  (0.11,0.69) |
| Southeast Asia | 317  (202,449) | 0.22  (0.14,0.31) | 1619  (993,2247) | 0.43  (0.27,0.6) | 2.25  (2.13,2.37) | 10725  (6640,15290) | 6.84  (4.32,9.75) | 52259  (31653,72923) | 13.52  (8.19,18.87) | 2.21  (2.07,2.35) |
| Central Asia | 364  (255,483) | 1.31  (0.92,1.74) | 516  (361,685) | 1.11  (0.77,1.47) | -0.55  (-0.83,-0.28) | 10563  (7464,14013) | 37.69  (26.66,50) | 15355  (10728,20511) | 31.2  (21.83,41.64) | -0.66  (-0.91,-0.42) |
| Eastern Europe | 2975  (2124,3943) | 1.64  (1.17,2.17) | 4625  (3257,6085) | 2.1  (1.47,2.75) | 0.24  (-0.13,0.61) | 83568  (59577,109788) | 48.12  (34.34,63) | 123207  (87301,162417) | 60.22  (42.49,79.04) | 0.15  (-0.23,0.54) |
| Central Europe | 1283  (908,1700) | 1.48  (1.04,1.96) | 2264  (1611,3038) | 1.72  (1.23,2.3) | 0.38  (0.21,0.56) | 33116  (23490,43665) | 38.88  (27.5,51.36) | 51709  (37169,69051) | 43.7  (31.44,58.21) | 0.3  (0.15,0.45) |
| Australasia | 86  (60,116) | 0.65  (0.46,0.88) | 259  (183,342) | 0.88  (0.63,1.16) | 1.11  (0.88,1.35) | 2093  (1461,2858) | 16.82  (11.69,23.01) | 6045  (4302,7957) | 22.97  (16.43,30.23) | 1.13  (0.92,1.35) |
| High-income  Asia Pacific | 302  (220,402) | 0.26  (0.19,0.35) | 733  (499,983) | 0.31  (0.22,0.42) | 0.7  (0.49,0.92) | 7792  (5651,10473) | 6.9  (4.99,9.28) | 16900  (11939,22693) | 9.07  (6.39,12.08) | 1.08  (0.88,1.28) |
| Western Europe | 2818  (1995,3794) | 0.8  (0.57,1.08) | 5011  (3494,6799) | 0.92  (0.65,1.23) | 0.78  (0.65,0.9) | 63924  (45520,85722) | 19.99  (14.25,26.73) | 107428  (76973,144397) | 23.28  (16.87,30.96) | 0.86  (0.73,0.99) |
| Southern Latin  America | 267  (188,365) | 1.03  (0.73,1.4) | 428  (295,563) | 0.86  (0.6,1.14) | -0.54  (-0.66,-0.42) | 6890  (4846,9306) | 26.85  (18.86,36.2) | 10267  (7192,13516) | 21.99  (15.49,28.84) | -0.59  (-0.73,-0.45) |
| High-income  North America | 2194  (1534,2977) | 1.05  (0.74,1.42) | 5700  (4036,7314) | 1.57  (1.12,2) | 1.4  (1.27,1.52) | 53882  (38326,72144) | 28.14  (20.15,37.38) | 147743  (107553,185881) | 44.48  (32.54,56.04) | 1.65  (1.53,1.76) |
| Andean Latin America | 126  (83,176) | 1.18  (0.78,1.65) | 375  (240,561) | 1.21  (0.78,1.81) | 0.01  (-0.2,0.22) | 3774  (2503,5320) | 33.39  (22.12,47.15) | 10500  (6752,15639) | 33.29  (21.33,49.61) | -0.1  (-0.31,0.11) |
| Caribbean | 145  (101,193) | 1.07  (0.74,1.43) | 550  (382,742) | 1.92  (1.34,2.6) | 1.84  (1.63,2.05) | 4279  (2944,5664) | 30.86  (21.27,40.87) | 15008  (10584,20030) | 53.69  (37.87,71.73) | 1.72  (1.49,1.94) |
| Central Latin America | 291  (204,390) | 0.69  (0.48,0.92) | 1238  (865,1673) | 0.91  (0.63,1.23) | 0.96  (0.59,1.32) | 8271  (5846,11016) | 18.03  (12.71,24.03) | 35219  (24857,47522) | 25.35  (17.88,34.21) | 1.17  (0.77,1.56) |
| Tropical Latin America | 398  (277,549) | 0.84  (0.58,1.14) | 1315  (915,1761) | 0.92  (0.64,1.24) | 0.07  (-0.06,0.19) | 10834  (7651,14745) | 21.4  (15,29.29) | 34288  (24075,45527) | 24.1  (16.93,32.02) | 0.1  (-0.05,0.25) |
| North Africa and Middle East | 406  (269,579) | 0.49  (0.33,0.71) | 1368  (921,1816) | 0.62  (0.42,0.83) | 0.91  (0.7,1.12) | 12362  (8207,17217) | 13.52  (8.99,19) | 41374  (28044,54860) | 17.04  (11.51,22.61) | 0.82  (0.62,1.01) |
| South Asia | 297  (203,417) | 0.11  (0.07,0.15) | 1799  (1195,2637) | 0.23  (0.16,0.35) | 2.58  (2.53,2.64) | 9110  (6177,12746) | 2.96  (2.02,4.15) | 52833  (35608,76798) | 6.56  (4.4,9.56) | 2.62  (2.57,2.68) |
| Central Sub-Saharan Africa | 34  (21,51) | 0.27  (0.17,0.41) | 157  (91,257) | 0.52  (0.3,0.86) | 2.18  (2.1,2.25) | 1018  (656,1533) | 7.4(4.75,  11.16) | 4759  (2771,7791) | 14.16  (8.23,23.17) | 2.12  (2.05,2.2) |
| Eastern Sub-Saharan Africa | 108  (64,153) | 0.28  (0.17,0.4) | 400  (249,617) | 0.45  (0.28,0.68) | 1.45  (1.38,1.53) | 3303  (1947,4686) | 7.82  (4.62,11.09) | 12142  (7462,19005) | 12.15  (7.6,18.73) | 1.35  (1.27,1.43) |
| Western Sub-Saharan Africa | 129  (85,189) | 0.3  (0.2,0.45) | 531  (342,764) | 0.54  (0.35,0.77) | 1.95  (1.91,1.99) | 3593  (2353,5147) | 8  (5.25,11.51) | 14896  (9582,21452) | 13.41  (8.62,19.25) | 1.75  (1.71,1.78) |
| Southern Sub-Saharan Africa | 95  (64,138) | 0.63  (0.42,0.92) | 424  (275,568) | 1.29  (0.83,1.72) | 2.76  (2.43,3.09) | 2629  (1777,3805) | 16.45  (11.11,23.76) | 11305  (7427,14968) | 32.41  (21.25,42.91) | 2.71  (2.39,3.03) |

BMI, body mass index; DALYs, disability-adjusted life years; ASMR, age-standardized mortality rate; ASDR, age-standardized DALY rate; EAPC, estimated annual percentage change; UI,

uncertainty interval; CI, confidence interval; SDI, socio-demographic index.

**Table S5** Deaths and DALYs for female colon and rectum cancer attributable to high BMI in 1990 and 2021, with corresponding EAPC from 1990 to 2021, in 204 countries

|  | **Deaths** | | | | | **DALYs** | | | | |
| --- | --- | --- | --- | --- | --- | --- | --- | --- | --- | --- |
| **Countries** | **Number of cases (95% UI), 1990** | **ASMR per 100,000 (95%**  **UI), 1990** | **Number of cases (95%**  **UI), 2021** | **ASMR per 100,000 (95% UI), 2021** | **EAPC of ASMR (95% CI),1990-2021** | **Number of cases (95% UI), 1990** | **ASDR per 100,000 (95% UI), 1990** | **Number of**  **cases (95% UI), 2021** | **ASDR per 100,000 (95% UI), 2021** | **EAPC of ASDR (95% CI), 1990-2021** |
| Afghanistan | 42  (8,90) | 1.21  (0.26,2.55) | 83  (21,168) | 1.42  (0.41,2.88) | 0.58  (0.47,0.7) | 1325  (243,2883) | 35.54  (6.84,76.25) | 2881  (666,5956) | 41.37  (10.45,84.87) | 0.53  (0.41,0.65) |
| Albania | 7  (3,13) | 0.7  (0.28,1.24) | 21  (8,37) | 0.89  (0.36,1.61) | 1.18  (0.95,1.4) | 164(66,293) | 15.4  (6.2,27.41) | 417  (167,763) | 18.46  (7.39,33.92) | 0.97  (0.75,1.19) |
| Algeria | 23  (9,38) | 0.47  (0.18,0.81) | 93  (38,157) | 0.65  (0.27,1.11) | 1.73  (1.46,2) | 637  (251,1091) | 10.35  (4.12,17.79) | 2444  (1009,4114) | 13.77  (5.67,23.11) | 1.23  (1.04,1.41) |
| American Samoa | 0  (0,0) | 1.67  (0.7,2.74) | 1  (0,1) | 2.36  (1.05,3.81) | 1.3  (1.18,1.41) | 5  (2,9) | 43.35  (18.8,69.98) | 15  (7,25) | 61.59  (28.3,99.68) | 1.29  (1.17,1.41) |
| Andorra | 0  (0,1) | 1.03  (0.38,1.88) | 1  (0,1) | 0.85  (0.34,1.54) | -0.31  (-0.46,-0.17) | 6  (2,12) | 23.01  (8.41,42.24) | 14  (6,26) | 18.67  (7.29,33.68) | -0.37  (-0.52,-0.22) |
| Angola | 4  (2,7) | 0.22  (0.08,0.38) | 28  (10,54) | 0.42  (0.14,0.84) | 2.07  (1.94,2.21) | 138  (53,237) | 5.96  (2.23,10.24) | 873  (303,1697) | 11.25  (3.9,21.72) | 1.96  (1.83,2.09) |
| Antigua and Barbuda | 0  (0,1) | 1  (0.41,1.6) | 1  (0,2) | 2.05  (0.85,3.26) | 2.38  (2.16,2.59) | 7  (3,11) | 24.49  (9.97,39.14) | 27  (12,43) | 47.16  (20.28,74.43) | 2.21  (2.01,2.41) |
| Argentina | 357  (155,579) | 1.99  (0.86,3.22) | 763  (342,1224) | 2.33  (1.05,3.73) | 0.78  (0.58,0.97) | 8042  (3543,13218) | 44.99  (19.81,74.01) | 16434  (7454,26798) | 54.23  (24.64,88.77) | 0.89  (0.72,1.06) |
| Armenia | 26  (11,41) | 1.69  (0.71,2.68) | 45  (20,72) | 1.76  (0.78,2.77) | 0.5  (0.32,0.68) | 714  (300,1119) | 44.4  (18.65,70.05) | 994  (443,1564) | 39.62  (17.64,62.37) | -0.08  (-0.24,0.08) |
| Australia | 219  (91,343) | 2  (0.83,3.15) | 424  (181,682) | 1.63  (0.7,2.62) | -0.88  (-0.97,-0.8) | 4980  (2017,7818) | 48.19  (19.4,75.52) | 8420  (3657,13524) | 37.48  (16.26,59.85) | -1.02  (-1.11,-0.93) |
| Austria | 150  (60,253) | 1.89  (0.76,3.11) | 120  (49,205) | 1.02  (0.41,1.73) | -2.04  (-2.12,-1.97) | 2847  (1143,4722) | 40.08  (16.22,65.38) | 2118  (858,3601) | 21.45  (8.85,35.93) | -2.03  (-2.1,-1.95) |
| Azerbaijan | 24  (10,40) | 0.82  (0.33,1.36) | 47  (20,78) | 0.82  (0.35,1.36) | 0.23  (0.05,0.41) | 717  (294,1172) | 23.67  (9.78,38.81) | 1355  (582,2270) | 21.91  (9.42,36.1) | -0.17  (-0.33,-0.01) |
| Bahamas | 1  (1,2) | 1.4  (0.59,2.26) | 5  (2,8) | 2.19  (0.91,3.61) | 1.72  (1.55,1.88) | 34  (14,55) | 37.01  (15.85,60.23) | 125  (52,204) | 54.34  (22.69,88.87) | 1.51  (1.37,1.65) |
| Bahrain | 1  (0,1) | 1.28  (0.53,2.1) | 5  (2,8) | 1.61  (0.7,2.58) | 0.49  (0.25,0.73) | 28  (11,46) | 31.42  (12.81,50.51) | 147  (62,236) | 36.3  (15.71,57.21) | 0.15  (-0.02,0.32) |
| Bangladesh | 15  (4,26) | 0.07  (0.02,0.12) | 99  (36,178) | 0.14  (0.05,0.26) | 2.92  (2.76,3.09) | 489  (144,899) | 1.95  (0.57,3.52) | 3174  (1152,5688) | 4.21  (1.54,7.6) | 3.06  (2.85,3.26) |
| Barbados | 3  (1,5) | 1.75  (0.74,2.84) | 9  (4,15) | 3.04  (1.33,5.02) | 2.43  (2.06,2.8) | 66  (28,108) | 43.07  (18.18,69.66) | 192  (85,314) | 69.55  (30.95,113.56) | 2.21  (1.87,2.55) |
| Belarus | 136  (56,227) | 1.63  (0.67,2.74) | 218  (90,370) | 2.1  (0.87,3.57) | 0.03  (-0.43,0.5) | 3343  (1362,5502) | 41.7  (17.03,68.68) | 4974  (2069,8505) | 51.68  (21.48,87.65) | -0.13  (-0.58,0.33) |
| Belgium | 174  (71,281) | 1.8  (0.74,2.91) | 196  (82,330) | 1.3  (0.55,2.16) | -1.03  (-1.11,-0.95) | 3308  (1374,5325) | 38.01  (16.04,61.1) | 3454  (1454,5715) | 28.13  (11.86,46.99) | -1  (-1.1,-0.91) |
| Belize | 0  (0,1) | 0.78  (0.33,1.27) | 2  (1,3) | 1.4  (0.64,2.27) | 1.93  (1.5,2.35) | 9  (4,14) | 19.12  (8.02,30.53) | 55  (26,86) | 34.43  (15.88,53.95) | 1.91  (1.52,2.31) |
| Benin | 3  (1,5) | 0.31(0.11,0.52) | 11  (4,19) | 0.42  (0.15,0.71) | 1.12  (1.04,1.2) | 90  (34,153) | 8.21  (3.08,13.85) | 306  (113,541) | 10.1  (3.72,17.58) | 0.77  (0.7,0.85) |
| Bermuda | 1  (0,2) | 2.62  (1.07,4.4) | 2  (1,3) | 2.33  (1.02,3.81) | -0.76  (-0.99,-0.52) | 21  (9,34) | 58.38  (24.31,94.9) | 37  (17,61) | 50.51  (22.94,82.72) | -0.84  (-1.08,-0.61) |
| Bhutan | 0  (0,1) | 0.3  (0.11,0.55) | 1  (0,2) | 0.35  (0.13,0.67) | 0.36  (0.24,0.47) | 13  (5,25) | 9.21  (3.16,16.89) | 32  (12,61) | 9.97  (3.86,19.44) | 0.12  (0.01,0.22) |
| Bolivia (Plurinational State of) | 18  (6,37) | 1.1  (0.35,2.21) | 79  (28,146) | 1.7  (0.61,3.11) | 1.41  (1.36,1.46) | 516  (161,1041) | 28.28  (8.79,57.1) | 2029  (728,3699) | 40.7  (14.51,74.31) | 1.1  (1.06,1.15) |
| Bosnia and Herzegovina | 28  (12,46) | 1.24  (0.52,2.07) | 66  (28,108) | 1.83  (0.79,2.98) | 1.41  (1.26,1.56) | 736  (313,1206) | 30.69  (12.98,50.73) | 1416  (617,2329) | 41.82  (18.36,68.68) | 1.18  (1,1.37) |
| Botswana | 2(1,4) | 0.71  (0.27,1.26) | 8  (3,14) | 1.07  (0.4,1.86) | 1.88  (1.44,2.32) | 56  (22,103) | 17.2  (6.65,31.35) | 203  (72,380) | 23.66  (8.6,42.88) | 1.62  (1.13,2.11) |
| Brazil | 400  (163,636) | 0.88  (0.36,1.41) | 1870  (785,3010) | 1.35  (0.56,2.16) | 1.28  (1.17,1.39) | 10722  (4484,17145) | 21.43  (8.9,34.08) | 47480  (20119,76267) | 34.35  (14.56,55.15) | 1.38  (1.28,1.48) |
| Brunei Darussalam | 1  (0,1) | 1.02  (0.39,1.75) | 2  (1,4) | 1.27  (0.52,2.16) | 1.23  (1.06,1.4) | 18  (7,30) | 29.24  (11.51,49.52) | 75  (31,125) | 35.52  (14.66,59.01) | 1.14  (0.96,1.33) |
| Bulgaria | 129  (57,203) | 1.99  (0.87,3.18) | 210  (88,351) | 2.47  (1.04,4.08) | 1.01  (0.81,1.22) | 3247  (1442,5112) | 49.28  (21.83,78.15) | 4408  (1853,7264) | 58.31  (24.48,96.37) | 0.8  (0.63,0.97) |
| Burkina Faso | 2  (1,4) | 0.09  (0.03,0.16) | 7  (2,12) | 0.14  (0.04,0.24) | 1.51  (1.42,1.59) | 64  (20,110) | 2.5  (0.76,4.35) | 207  (61,367) | 3.63  (1.05,6.38) | 1.38  (1.27,1.49) |
| Burundi | 3  (1,5) | 0.22  (0.07,0.41) | 6  (2,11) | 0.27  (0.09,0.51) | 0.16  (-0.03,0.35) | 80  (24,147) | 5.93  (1.81,10.87) | 177  (60,319) | 6.78  (2.29,12.17) | -0.09  (-0.3,0.12) |
| Cabo Verde | 0  (0,0) | 0.2  (0.08,0.36) | 2  (1,3) | 0.72  (0.28,1.21) | 3.78  (3.42,4.14) | 6  (2,11) | 4.93  (1.86,8.45) | 37  (15,62) | 14.39  (5.72,24.01) | 3.1  (2.74,3.47) |
| Cambodia | 7  (2,14) | 0.27  (0.08,0.5) | 31  (11,53) | 0.42  (0.15,0.73) | 1.31  (1.21,1.41) | 236  (67,449) | 7.91  (2.26,14.9) | 907  (326,1603) | 11.57  (4.2,20.19) | 1.1  (1.01,1.2) |
| Cameroon | 12  (5,20) | 0.58  (0.23,0.97) | 50  (19,90) | 0.87  (0.33,1.55) | 1.5  (1.42,1.57) | 344  (135,574) | 14.05  (5.58,23.39) | 1369  (503,2448) | 19.55  (7.26,35.21) | 1.22  (1.14,1.29) |
| Canada | 364  (164,578) | 1.94  (0.87,3.08) | 642  (287,1028) | 1.49  (0.67,2.39) | -0.69  (-0.79,-0.58) | 7898  (3527,12414) | 44.58  (19.89,69.79) | 12266  (5522,19435) | 33.36  (15.17,53.31) | -0.78  (-0.86,-0.69) |
| Central African Republic | 2  (1,4) | 0.28  (0.1,0.54) | 6  (2,12) | 0.47  (0.17,0.94) | 1.7  (1.65,1.75) | 56  (19,114) | 7.76  (2.78,15.52) | 190  (71,392) | 12.94  (4.79,26.83) | 1.65  (1.6,1.71) |
| Chad | 3  (1,5) | 0.2  (0.07,0.34) | 9  (4,16) | 0.37  (0.14,0.65) | 2.28  (2.17,2.39) | 73  (27,128) | 4.91  (1.83,8.6) | 256  (102,456) | 8.87  (3.56,15.82) | 2.14  (2.03,2.25) |
| Chile | 75  (31,122) | 1.42  (0.58,2.29) | 262  (116,422) | 1.79  (0.8,2.87) | 0.97  (0.86,1.09) | 1771  (733,2831) | 31.92  (13.18,51.07) | 5629  (2569,8981) | 40.78  (18.69,65.01) | 1.04  (0.94,1.15) |
| China | 1774  (616,3029) | 0.43  (0.15,0.74) | 8236  (3262,14370) | 0.75  (0.3,1.31) | 1.63  (1.51,1.75) | 51344  (17481,88161) | 11.26  (3.85,19.31) | 202371  (80646,353994) | 18.49  (7.34,32.36) | 1.39  (1.26,1.53) |
| Colombia | 82  (34,133) | 0.95  (0.39,1.54) | 412  (180,691) | 1.35  (0.59,2.26) | 1.04  (0.89,1.18) | 2111  (883,3465) | 22.31  (9.32,36.34) | 10088  (4373,16705) | 33.67  (14.59,55.69) | 1.27  (1.1,1.45) |
| Comoros | 0  (0,1) | 0.39  (0.13,0.7) | 2  (1,3) | 0.68  (0.24,1.23) | 1.68  (1.6,1.75) | 11  (4,20) | 10.05  (3.32,17.75) | 47  (17,86) | 16.6  (5.92,30.11) | 1.46  (1.34,1.57) |
| Congo | 3  (1,5) | 0.43  (0.15,0.79) | 12  (5,22) | 0.87  (0.33,1.52) | 2.13  (1.98,2.28) | 82  (29,151) | 12.25  (4.23,22.39) | 381  (153,681) | 23  (9.06,40.89) | 1.9  (1.71,2.09) |
| Cook Islands | 0  (0,0) | 1.15  (0.49,1.93) | 0  (0,0) | 0.93  (0.42,1.6) | -0.78  (-1.03,-0.53) | 2  (1,3) | 30.23  (13.14,50.71) | 3  (1,5) | 23.1  (10.67,39.8) | -0.91  (-1.19,-0.64) |
| Costa Rica | 9  (4,15) | 1.04  (0.43,1.71) | 54  (24,87) | 1.8  (0.78,2.88) | 1.86  (1.71,2) | 234  (99,377) | 25.06  (10.57,40.49) | 1323  (571,2090) | 44.57  (19.18,70.46) | 1.93  (1.77,2.09) |
| Croatia | 72  (32,116) | 2.03  (0.9,3.29) | 136  (59,223) | 2.41  (1.04,3.91) | 0.73  (0.55,0.91) | 1588  (706,2559) | 44.38  (19.59,71.29) | 2566  (1103,4160) | 52.01  (22.27,84.48) | 0.62  (0.43,0.82) |
| Cuba | 69  (28,108) | 1.34  (0.55,2.11) | 241  (101,412) | 2.23  (0.94,3.79) | 1.82  (1.73,1.91) | 1746  (716,2800) | 33.65  (13.82,54.12) | 5416  (2263,9021) | 53.96  (22.52,89.72) | 1.66  (1.54,1.79) |
| Cyprus | 4  (2,7) | 1.12  (0.43,1.87) | 12  (5,20) | 1.13  (0.48,1.88) | 0.19  (0.05,0.33) | 96  (37,162) | 22.76  (8.76,37.99) | 237  (100,399) | 22.42  (9.55,37.3) | 0.12  (-0.02,0.26) |
| Czechia | 274  (119,438) | 3.2  (1.39,5.13) | 266  (117,433) | 2.01  (0.89,3.3) | -1.87  (-2.04,-1.7) | 5771  (2519,9151) | 71.25  (31.09,113.46) | 5112  (2232,8376) | 43.76  (19.09,72.33) | -1.94  (-2.09,-1.8) |
| Côte d'Ivoire | 5  (2,8) | 0.27  (0.1,0.45) | 19  (7,32) | 0.37  (0.14,0.63) | 1.39  (1.27,1.52) | 144  (55,249) | 6.65  (2.61,11.13) | 552  (207,961) | 9.03  (3.42,15.63) | 1.35  (1.22,1.48) |
| Democratic People's Republic of Korea | 23  (7,46) | 0.26  (0.08,0.51) | 102  (35,199) | 0.52  (0.18,1.01) | 2.44  (2.34,2.55) | 592  (167,1193) | 5.99  (1.75,12.01) | 2148  (741,4236) | 11.18  (3.82,22.12) | 2.14  (2.02,2.25) |
| Democratic Republic of the Congo | 16  (6,28) | 0.2  (0.07,0.35) | 77  (28,140) | 0.41  (0.15,0.75) | 2.44  (2.28,2.6) | 463  (166,818) | 4.99  (1.84,8.79) | 2193  (853,3919) | 10.08  (3.81,18.37) | 2.43  (2.26,2.59) |
| Denmark | 82  (35,133) | 1.7  (0.73,2.77) | 118  (49,198) | 1.65  (0.69,2.75) | -0.42  (-0.7,-0.15) | 1681  (711,2722) | 39.18  (16.18,63.01) | 2126  (904,3546) | 34.21  (14.49,56.78) | -0.7  (-0.97,-0.44) |
| Djibouti | 0  (0,0) | 0.21  (0.07,0.41) | 1  (0,2) | 0.38  (0.13,0.72) | 1.81  (1.75,1.87) | 5  (2,9) | 5.69  (1.9,11.09) | 34  (11,66) | 9.69  (3.24,18.53) | 1.65  (1.57,1.72) |
| Dominica | 1  (0,1) | 1.44  (0.62,2.39) | 1  (0,2) | 2.16  (0.94,3.49) | 1.44  (1.35,1.53) | 11  (5,19) | 32.73  (14.26,52.32) | 21  (9,33) | 47.94  (21.54,76.74) | 1.38  (1.3,1.46) |
| Dominican Republic | 8  (3,14) | 0.47  (0.17,0.79) | 42  (17,77) | 0.81  (0.32,1.48) | 2.18  (1.98,2.39) | 248  (97,425) | 12.05  (4.59,20.57) | 1114  (458,2010) | 21.07(  8.65,37.87) | 2.16  (2,2.31) |
| Ecuador | 18  (8,29) | 0.7  (0.29,1.13) | 114  (48,188) | 1.33  (0.55,2.2) | 2.44  (2.13,2.76) | 468  (202,745) | 16.46  (7.11,26.51) | 2818  (1224,4580) | 32.35  (14.04,52.6) | 2.4  (2.1,2.7) |
| Egypt | 92  (39,147) | 0.75  (0.32,1.22) | 470  (211,733) | 1.93  (0.87,2.99) | 4.13  (3.74,4.52) | 2889  (1245,4617) | 18.99  (8.11,30.49) | 14034  (6268,21920) | 44.24  (19.93,68.68) | 3.61  (3.28,3.94) |
| El Salvador | 9  (4,15) | 0.59  (0.25,0.96) | 42  (18,70) | 1.15  (0.49,1.9) | 2.26  (2.1,2.42) | 257  (110,420) | 15.43  (6.6,25.2) | 1031  (438,1716) | 29.09  (12.35,48.49) | 2.14  (1.99,2.29) |
| Equatorial Guinea | 0  (0,1) | 0.39  (0.15,0.71) | 2  (1,5) | 0.8  (0.29,1.62) | 2.44  (2.34,2.55) | 13  (5,24) | 10.89  (3.99,19.66) | 69  (24,146) | 20.34  (7.1,42.23) | 2.11  (1.99,2.22) |
| Eritrea | 1  (0,2) | 0.2  (0.07,0.35) | 6  (2,10) | 0.35  (0.13,0.62) | 1.84  (1.77,1.91) | 44  (16,77) | 5.65  (2.05,9.77) | 168  (61,296) | 9.16  (3.43,16.26) | 1.61  (1.52,1.69) |
| Estonia | 26  (11,43) | 1.96  (0.82,3.15) | 36(16,62) | 1.87  (0.84,3.17) | -0.36  (-0.52,-0.2) | 610  (254,983) | 47.57  (19.82,76.54) | 659  (292,1117) | 40.46  (17.79,68.5) | -0.79  (-0.98,-0.61) |
| Eswatini | 2  (1,4) | 1.4  (0.54,2.52) | 7  (3,12) | 2.29  (0.9,4.02) | 1.89  (1.39,2.39) | 54  (22,96) | 32.31  (12.76,57.33) | 184  (70,337) | 52.93  (20.4,94.85) | 1.91  (1.35,2.48) |
| Ethiopia | 38  (10,68) | 0.39  (0.11,0.71) | 65  (23,112) | 0.31  (0.11,0.54) | -1.35  (-1.61,-1.09) | 1210  (308,2252) | 10.88  (2.86,19.79) | 1888  (663,3342) | 7.86  (2.78,13.75) | -1.76  (-2.04,-1.48) |
| Fiji | 2  (1,3) | 0.99  (0.41,1.7) | 5  (2,9) | 1.4(  0.62,2.25) | 1.22  (1.01,1.44) | 55  (24,92) | 26.1  (11.23,44.14) | 149  (66,246) | 34.79  (15.5,57.11) | 1.06  (0.82,1.3) |
| Finland | 58  (24,95) | 1.25  (0.53,2.05) | 93  (38,154) | 1.14  (0.48,1.88) | -0.34  (-0.45,-0.24) | 1159  (486,1921) | 27.24  (11.45,45.33) | 1641  (700,2723) | 24.63  (10.32,40.73) | -0.39  (-0.46,-0.31) |
| France | 729  (296,1185) | 1.35  (0.56,2.17) | 1237  (510,2032) | 1.25  (0.51,2.05) | -0.18  (-0.29,-0.08) | 13349  (5555,21438) | 28.09  (11.75,45.32) | 20723  (8683,34145) | 27.19  (11.52,44.89) | -0.01  (-0.11,0.1) |
| Gabon | 3  (1,6) | 0.96  (0.35,1.94) | 7  (3,13) | 1.39  (0.58,2.51) | 1.08  (0.9,1.25) | 80  (29,163) | 24.98  (9.14,50.14) | 205  (82,372) | 34.19  (13.88,62.04) | 0.88  (0.68,1.07) |
| Gambia | 0  (0,1) | 0.2  (0.07,0.35) | 1  (1,3) | 0.3  (0.11,0.54) | 1.13  (0.95,1.31) | 9(3,16) | 5.32  (1.83,9.29) | 43  (17,76) | 7.7  (2.98,13.76) | 1  (0.79,1.21) |
| Georgia | 35  (14,54) | 0.91  (0.37,1.43) | 43  (19,71) | 1.2  (0.52,1.96) | 1.93  (1.46,2.39) | 908  (373,1424) | 24.55  (10.07,38.45) | 976  (425,1599) | 29.28  (12.68,47.92) | 1.41  (1.03,1.79) |
| Germany | 2039  (854,3322) | 2.39  (1,3.89) | 1696  (700,2837) | 1.39  (0.57,2.32) | -2.08  (-2.22,-1.93) | 39310  (16443,63668) | 51.49  (21.56,83.03) | 30336  (12689,50683) | 30.48  (13.06,49.83) | -1.96  (-2.11,-1.82) |
| Ghana | 8  (3,14) | 0.25  (0.09,0.43) | 52  (19,91) | 0.59  (0.21,1.03) | 2.9  (2.84,2.96) | 261  (97,452) | 6.85  (2.52,11.93) | 1511  (576,2625) | 14.41  (5.41,25.14) | 2.44  (2.37,2.51) |
| Greece | 107  (45,173) | 1.29  (0.53,2.08) | 213  (89,346) | 1.4  (0.6,2.25) | -0.06  (-0.29,0.16) | 2235  (944,3583) | 27.43  (11.6,44.01) | 3698  (1579,5947) | 29.92  (12.65,48.68) | 0.05  (-0.11,0.21) |
| Greenland | 1  (0,1) | 4.97  (2.2,8.23) | 1  (0,2) | 2.92  (1.26,4.94) | -1.93  (-2.05,-1.81) | 22  (9,36) | 120.19  (52.25,200.29) | 24  (10,40) | 70.21  (29.31,119.4) | -1.95  (-2.06,-1.83) |
| Grenada | 0  (0,1) | 1.1  (0.44,1.79) | 1  (0,2) | 1.89  (0.82,3.06) | 2.32  (2.02,2.63) | 11  (4,18) | 30.21  (12.23,49.68) | 28  (13,45) | 47.19  (21.14,75) | 2.01  (1.71,2.3) |
| Guam | 0  (0,1) | 1.3  (0.5,2.18) | 1  (0,2) | 1.04  (0.45,1.68) | -0.06  (-0.46,0.34) | 11  (5,18) | 28.89  (11.53,47.58) | 33  (14,52) | 30.98  (13.68,49.59) | 0.71  (0.39,1.03) |
| Guatemala | 10  (4,16) | 0.62  (0.26,1.01) | 59  (26,97) | 1.03  (0.45,1.7) | 1.55  (1.27,1.83) | 275  (119,437) | 14.34  (6.18,23.14) | 1528  (684,2500) | 24.72  (11,40.51) | 1.76  (1.52,2) |
| Guinea | 3  (1,6) | 0.2  (0.08,0.34) | 7  (3,14) | 0.27  (0.09,0.48) | 0.95  (0.91,0.98) | 96  (35,163) | 5.46  (2.01,9.32) | 219  (77,410) | 6.96  (2.46,12.96) | 0.84  (0.8,0.87) |
| Guinea-Bissau | 1  (0,1) | 0.32  (0.12,0.6) | 2  (1,4) | 0.55  (0.21,0.95) | 1.96  (1.89,2.03) | 21  (7,39) | 8.85  (3.2,16.63) | 67  (24,123) | 14.26  (5.14,25.42) | 1.74  (1.67,1.81) |
| Guyana | 2  (1,3) | 0.86  (0.33,1.41) | 5  (2,8) | 1.37  (0.59,2.29) | 1.91  (1.67,2.16) | 45  (17,73) | 21.53  (8.23,35.43) | 124  (53,209) | 34.35  (14.8,57.73) | 1.9  (1.66,2.14) |
| Haiti | 9  (2,16) | 0.52  (0.15,0.95) | 32  (12,59) | 0.82  (0.29,1.54) | 1.78  (1.67,1.9) | 274  (81,512) | 14.43  (4.29,26.82) | 1015  (364,1901) | 22.35  (8.15,41.63) | 1.72  (1.59,1.84) |
| Honduras | 4  (2,8) | 0.43  (0.15,0.72) | 28  (12,50) | 0.86  (0.36,1.52) | 2.33  (2.05,2.62) | 133  (49,225) | 11.44  (4.14,19.36) | 771  (315,1316) | 21.34  (8.73,36.69) | 2.07  (1.81,2.32) |
| Hungary | 256  (105,405) | 2.91  (1.2,4.56) | 320  (143,523) | 2.62  (1.18,4.27) | -0.47  (-0.72,-0.22) | 5642  (2346,8797) | 66.52  (27.69,103.88) | 6622  (2958,10700) | 62.2  (27.77,100.68) | -0.35  (-0.61,-0.1) |
| Iceland | 2  (1,4) | 1.49  (0.63,2.42) | 4  (2,7) | 1.25  (0.55,2.11) | -0.55  (-0.67,-0.43) | 52  (22,84) | 34.91  (14.87,56.85) | 81  (35,134) | 27.22  (11.86,44.99) | -0.85  (-0.95,-0.75) |
| India | 235  (80,395) | 0.1  (0.03,0.17) | 1294  (503,2107) | 0.21  (0.08,0.34) | 2.5  (2.37,2.63) | 7951  (2721,13170) | 2.96  (1.01,4.95) | 39082  (15078,63342) | 5.96  (2.3,9.66) | 2.28  (2.13,2.43) |
| Indonesia | 115  (35,207) | 0.21  (0.06,0.38) | 689  (253,1280) | 0.54  (0.2,0.99) | 3.3  (3.1,3.5) | 3854  (1224,6938) | 6.25  (1.94,11.27) | 21254  (7766,39737) | 15.06  (5.53,27.86) | 3.08  (2.84,3.32) |
| Iran (Islamic Republic of) | 74  (32,124) | 0.63  (0.27,1.05) | 388  (167,606) | 1.05  (0.45,1.65) | 2.09  (1.83,2.34) | 2259  (962,3728) | 16.16  (6.94,26.89) | 10337  (4438,15966) | 25.14  (10.82,38.86) | 1.81  (1.55,2.07) |
| Iraq | 23  (9,38) | 0.56  (0.21,0.91) | 88  (37,151) | 0.75  (0.32,1.28) | 1.12  (0.9,1.35) | 705  (262,1164) | 15.86  (5.94,26.05) | 2642  (1112,4524) | 19.31  (8.06,32.66) | 0.82  (0.64,1) |
| Ireland | 42  (18,70) | 1.87  (0.79,3.07) | 56  (24,93) | 1.24  (0.55,2.08) | -1.11  (-1.21,-1.02) | 915  (377,1498) | 42.98  (17.68,70.29) | 1116  (490,1847) | 27.43  (12.02,45.22) | -1.28  (-1.37,-1.19) |
| Israel | 52  (22,83) | 2.03  (0.87,3.22) | 105  (46,172) | 1.41  (0.62,2.32) | -1.82  (-2.15,-1.48) | 1128  (474,1797) | 43.92  (18.6,70.25) | 1904  (851,3113) | 28.72  (12.72,46.79) | -1.9  (-2.23,-1.58) |
| Italy | 735  (304,1207) | 1.38  (0.57,2.27) | 1185  (503,1963) | 1.22  (0.53,2) | -0.51  (-0.62,-0.41) | 15244  (6340,25187) | 30.86  (12.78,50.72) | 20400  (8841,33462) | 26.1  (11.29,42.49) | -0.71  (-0.83,-0.59) |
| Jamaica | 10  (4,16) | 1.02  (0.44,1.59) | 33  (15,53) | 1.96  (0.9,3.19) | 2.08  (1.82,2.35) | 228  (99,359) | 24.74  (10.78,38.79) | 788  (370,1296) | 49.46  (23.27,81.34) | 2.27  (1.98,2.56) |
| Japan | 662  (250,1071) | 0.69  (0.26,1.12) | 1758  (658,2918) | 0.68  (0.26,1.09) | -0.22  (-0.33,-0.11) | 15614  (5911,25390) | 16.66  (6.29,27.05) | 28041  (10732,44867) | 15.31  (5.96,24.46) | -0.45  (-0.56,-0.33) |
| Jordan | 10  (4,16) | 1.49  (0.63,2.48) | 51  (21,87) | 1.59  (0.67,2.74) | 0.34  (-0.07,0.75) | 302  (133,501) | 39.96  (17.35,66.94) | 1424  (586,2463) | 37.18  (15.54,64.32) | -0.25  (-0.64,0.13) |
| Kazakhstan | 126  (53,203) | 1.62  (0.68,2.61) | 135  (60,217) | 1.31  (0.58,2.09) | -0.45  (-0.73,-0.17) | 3303  (1385,5261) | 42  (17.57,66.85) | 3462  (1533,5589) | 31.98  (14.12,51.61) | -0.75  (-0.98,-0.51) |
| Kenya | 11  (4,18) | 0.24  (0.09,0.41) | 70  (27,119) | 0.56  (0.22,0.96) | 3.27  (3.04,3.5) | 342  (128,574) | 7.04  (2.63,11.82) | 2163  (862,3692) | 15.27  (6.02,25.97) | 3.02  (2.83,3.21) |
| Kiribati | 0  (0,0) | 0.72  (0.29,1.21) | 0  (0,1) | 1.06  (0.44,1.72) | 1.2  (1.1,1.3) | 5  (2,8) | 21.02  (8.69,34.62) | 13  (6,23) | 28.64  (12.03,47.79) | 0.98  (0.88,1.07) |
| Kuwait | 2  (1,3) | 0.89  (0.4,1.4) | 15  (7,24) | 1.22  (0.57,1.95) | 1.5  (0.86,2.14) | 59  (26,92) | 22.4  (10.02,35.07) | 461  (222,743) | 28.99  (13.79,46.49) | 1.14  (0.47,1.82) |
| Kyrgyzstan | 21  (9,34) | 1.18  (0.49,1.91) | 24  (11,38) | 0.91  (0.39,1.44) | -0.64  (-0.8,-0.47) | 569  (239,923) | 31.85  (13.36,51.53) | 686  (298,1077) | 23.63  (10.28,36.97) | -0.84  (-0.98,-0.7) |
| Lao People's Democratic Republic | 4  (1,8) | 0.34  (0.09,0.68) | 13  (5,24) | 0.54  (0.2,0.99) | 1.59  (1.48,1.69) | 115  (29,234) | 9.44  (2.49,19.24) | 402  (149,735) | 14.62  (5.55,27.11) | 1.49  (1.36,1.62) |
| Latvia | 49  (21,80) | 2.08  (0.89,3.4) | 56  (25,92) | 2  (0.87,3.29) | -0.06  (-0.26,0.15) | 1143  (491,1850) | 51.3  (22.11,82.86) | 1069  (466,1751) | 45.3  (19.73,74.8) | -0.4  (-0.59,-0.21) |
| Lebanon | 14  (5,24) | 1.35  (0.51,2.36) | 45  (19,73) | 1.31  (0.55,2.15) | -0.02  (-0.19,0.15) | 361  (137,624) | 31.55  (12.05,54.41) | 928  (389,1509) | 28.57  (12,46.29) | -0.33  (-0.45,-0.2) |
| Lesotho | 3  (1,6) | 0.66  (0.26,1.12) | 11  (4,20) | 1.74  (0.65,3.23) | 4.26  (3.69,4.82) | 86  (33,147) | 15.66  (6.08,26.76) | 294  (109,551) | 42.97  (15.95,80.67) | 4.45  (3.84,5.07) |
| Liberia | 2  (1,3) | 0.37  (0.13,0.62) | 6  (2,12) | 0.6  (0.2,1.2) | 1.91  (1.73,2.08) | 54  (20,91) | 9.6  (3.59,16.31) | 177  (58,347) | 14.85  (5.01,29.65) | 1.68  (1.5,1.85) |
| Libya | 13  (5,22) | 1.46  (0.58,2.53) | 59  (25,101) | 2.3  (0.96,4.03) | 1.95  (1.78,2.13) | 358  (146,616) | 38.11  (15.44,65.69) | 1732  (737,2958) | 58.32  (25.09,99.95) | 1.77  (1.63,1.92) |
| Lithuania | 47  (20,76) | 1.66  (0.72,2.68) | 76  (33,127) | 1.87  (0.81,3.07) | 0.31  (0.15,0.47) | 1084  (474,1770) | 39.89  (17.38,64.7) | 1428  (616,2343) | 41.51  (17.75,68.52) | -0.02  (-0.18,0.15) |
| Luxembourg | 7  (3,11) | 2.03  (0.84,3.21) | 9  (4,15) | 1.39  (0.59,2.28) | -1.13  (-1.32,-0.95) | 137  (56,216) | 44.15  (18.02,69.63) | 161  (69,265) | 27.77  (11.83,45.84) | -1.33  (-1.54,-1.13) |
| Madagascar | 6  (2,11) | 0.26  (0.1,0.46) | 24  (10,41) | 0.44  (0.17,0.77) | 1.59  (1.41,1.78) | 185  (66,329) | 6.76  (2.44,11.92) | 721  (280,1252) | 10.84  (4.36,18.77) | 1.47  (1.28,1.67) |
| Malawi | 2  (1,4) | 0.1  (0.03,0.18) | 8  (3,15) | 0.18  (0.06,0.34) | 1.61  (1.5,1.73) | 73  (24,126) | 2.98  (0.99,5.25) | 256  (84,499) | 5.18  (1.69,10.06) | 1.59  (1.46,1.71) |
| Malaysia | 45  (19,73) | 0.92  (0.38,1.49) | 219  (91,358) | 1.59  (0.66,2.58) | 1.74  (1.65,1.84) | 1348  (567,2225) | 25.5  (10.78,42.26) | 5994  (2572,9835) | 40.28  (17.05,66.46) | 1.41  (1.27,1.55) |
| Maldives | 0  (0,0) | 0.34  (0.1,0.63) | 0  (0,1) | 0.27  (0.11,0.47) | -0.97  (-1.14,-0.79) | 5  (1,10) | 10.49  (2.97,19.65) | 13  (5,23) | 7.57  (3.02,12.96) | -1.32  (-1.48,-1.17) |
| Mali | 6  (2,10) | 0.29  (0.11,0.5) | 15  (5,27) | 0.35  (0.13,0.62) | 0.6  (0.51,0.69) | 172  (67,295) | 7.8  (3.03,13.28) | 456  (169,843) | 9.16  (3.38,16.23) | 0.57  (0.46,0.67) |
| Malta | 3  (1,5) | 1.35  (0.54,2.16) | 8  (4,14) | 1.47  (0.63,2.48) | 0  (-0.13,0.12) | 72  (30,116) | 30.61  (12.57,49.14) | 162  (69,275) | 32.73  (14.14,54.84) | -0.03  (-0.15,0.08) |
| Marshall Islands | 0  (0,0) | 1.33  (0.56,2.3) | 0  (0,1) | 1.79  (0.71,3.01) | 0.81  (0.68,0.95) | 3  (1,6) | 36.4  (15.23,63.03) | 10  (4,18) | 48.3  (18.98,83.3) | 0.77  (0.62,0.92) |
| Mauritania | 3  (1,5) | 0.56  (0.23,0.99) | 9  (3,14) | 0.88  (0.34,1.44) | 1.51  (1.41,1.6) | 76  (31,133) | 14.04  (5.78,24.65) | 227  (90,381) | 19.92  (7.86,32.87) | 1.15  (1.06,1.24) |
| Mauritius | 2  (1,3) | 0.52  (0.21,0.86) | 12  (5,19) | 1.21  (0.52,1.92) | 2.46  (2.26,2.66) | 56  (23,91) | 13.43  (5.46,21.74) | 309  (132,492) | 31.59  (13.52,50.11) | 2.4  (2.19,2.61) |
| Mexico | 142  (59,225) | 0.71  (0.3,1.15) | 764  (340,1198) | 1.13  (0.51,1.78) | 1.56  (1.43,1.69) | 3715  (1546,5861) | 15.99  (6.64,25.26) | 20669  (9221,32562) | 29.46  (13.13,46.36) | 1.99  (1.87,2.11) |
| Micronesia (Federated States of) | 0  (0,1) | 1.44  (0.54,2.68) | 1  (0,1) | 1.8  (0.68,3.19) | 0.71  (0.67,0.76) | 11  (4,20) | 40.75  (15.31,75.24) | 21  (8,38) | 48.73  (18.82,87.28) | 0.58  (0.53,0.64) |
| Monaco | 1  (0,2) | 2  (0.75,3.48) | 1  (1,3) | 2.46  (1.07,4.29) | 0.77  (0.66,0.88) | 17  (6,29) | 44.64  (16.56,78.49) | 27  (12,47) | 53.66  (23.43,92.6) | 0.7  (0.59,0.82) |
| Mongolia | 4  (2,7) | 0.7  (0.29,1.24) | 10  (4,18) | 0.8  (0.32,1.4) | 0.17  (0.01,0.33) | 116  (48,204) | 19.53  (8.09,34.45) | 297  (120,517) | 20.62  (8.31,35.88) | -0.08  (-0.24,0.09) |
| Montenegro | 4  (2,6) | 1.13  (0.49,1.84) | 10  (4,17) | 1.9  (0.82,3.15) | 1.79  (1.61,1.96) | 94  (40,152) | 26.84  (11.45,43.43) | 215  (94,351) | 39.88  (17.33,65.2) | 1.41  (1.17,1.65) |
| Morocco | 46  (17,82) | 0.65  (0.24,1.14) | 202  (74,376) | 1.16  (0.44,2.17) | 2.14  (2.03,2.24) | 1360  (498,2457) | 17.57  (6.54,31.72) | 5823  (2117,10916) | 31.34  (11.46,58.5) | 2.11  (2,2.21) |
| Mozambique | 3  (1,4) | 0.09  (0.03,0.16) | 10  (4,18) | 0.18  (0.06,0.33) | 2.63  (2.41,2.86) | 70  (24,122) | 2.15  (0.74,3.75) | 269  (97,476) | 4.17  (1.5,7.45) | 2.58  (2.35,2.8) |
| Myanmar | 48  (15,98) | 0.37  (0.12,0.74) | 131  (50,234) | 0.46  (0.18,0.84) | 0.48  (0.33,0.63) | 1615  (511,3307) | 11.39  (3.63,23.45) | 3934  (1498,6936) | 13.22  (5,23.27) | 0.17  (-0.01,0.35) |
| Namibia | 1  (0,2) | 0.34  (0.13,0.59) | 4  (2,8) | 0.59  (0.24,1.02) | 1.64  (1.4,1.88) | 34  (14,58) | 8.92  (3.53,15.38) | 122  (46,212) | 14.29  (5.49,24.88) | 1.37  (1.11,1.63) |
| Nauru | 0  (0,0) | 2.26  (0.88,4.26) | 0  (0,0) | 2.6  (0.98,4.47) | 0.47  (0.43,0.51) | 2  (1,3) | 64.13(24.23,123.46) | 3  (1,5) | 71.55  (26.35,123.41) | 0.37  (0.31,0.42) |
| Nepal | 4  (1,8) | 0.08  (0.02,0.16) | 19  (6,35) | 0.14  (0.05,0.26) | 1.98  (1.52,2.44) | 156  (43,295) | 2.72  (0.74,5.15) | 644  (218,1194) | 4.57  (1.53,8.53) | 1.99  (1.51,2.47) |
| Netherlands | 237  (99,395) | 1.93  (0.8,3.21) | 377  (155,635) | 1.85  (0.76,3.11) | -0.03  (-0.18,0.13) | 4860  (2039,8026) | 43.33  (18.17,71.05) | 7546  (3052,12592) | 42.36  (17.36,70.36) | 0.04  (-0.13,0.21) |
| New Zealand | 59  (24,96) | 2.69  (1.11,4.36) | 92  (37,151) | 1.91  (0.77,3.14) | -1.05  (-1.13,-0.97) | 1343  (556,2163) | 65.32  (27.19,105.65) | 1831  (758,3000) | 42.63  (17.79,69.83) | -1.38  (-1.46,-1.3) |
| Nicaragua | 5  (2,7) | 0.56  (0.23,0.92) | 24  (10,39) | 0.89  (0.38,1.46) | 1.93  (1.67,2.19) | 130  (54,210) | 14.39  (6,23.17) | 626  (269,1036) | 22.27  (9.61,36.83) | 1.77  (1.55,2) |
| Niger | 3  (1,5) | 0.22  (0.08,0.39) | 12  (4,23) | 0.31  (0.1,0.57) | 1.06  (1.02,1.11) | 88  (32,161) | 5.86  (2.15,10.53) | 354  (133,667) | 7.54  (2.75,14.21) | 0.81  (0.76,0.86) |
| Nigeria | 53  (22,88) | 0.26  (0.11,0.44) | 219  (83,382) | 0.5  (0.2,0.86) | 2.28  (2.22,2.34) | 1304  (538,2194) | 6.05  (2.5,10.1) | 5865  (2207,10467) | 11.21  (4.24,19.59) | 2.08  (2.02,2.14) |
| Niue | 0  (0,0) | 1.08  (0.42,1.85) | 0  (0,0) | 1.5  (0.68,2.49) | 0.93  (0.88,0.99) | 0  (0,1) | 28.44  (11.34,48.84) | 0  (0,1) | 38.45  (17.86,63.49) | 0.75  (0.68,0.82) |
| North Macedonia | 14  (6,24) | 1.54  (0.64,2.51) | 37  (17,64) | 2.28  (1.01,3.93) | 1.24  (0.91,1.58) | 364  (147,593) | 36.5  (14.77,59.46) | 826  (369,1439) | 47.86  (21.33,82.75) | 0.87  (0.58,1.15) |
| Northern Mariana Islands | 0  (0,0) | 1.55  (0.62,2.68) | 0  (0,1) | 1.83  (0.79,2.86) | 0.47  (0.07,0.87) | 4  (2,7) | 40.85  (16.74,69.86) | 13  (6,19) | 45.81  (20.27,71.76) | 0.35  (-0.07,0.78) |
| Norway | 72  (30,117) | 1.74  (0.73,2.81) | 98  (43,162) | 1.59  (0.69,2.63) | -0.44  (-0.56,-0.32) | 1419  (595,2293) | 39.87  (16.96,64.66) | 1743  (755,2862) | 33.21  (14.5,54.14) | -0.69  (-0.83,-0.55) |
| Oman | 1  (0,2) | 0.3  (0.12,0.54) | 3  (1,6) | 0.42  (0.18,0.7) | 1.45  (1.25,1.65) | 27  (11,47) | 8.17  (3.31,14.44) | 99  (41,159) | 10.25  (4.3,16.69) | 1.07  (0.83,1.31) |
| Pakistan | 53  (18,90) | 0.21  (0.07,0.36) | 285  (114,474) | 0.48  (0.19,0.79) | 2.74  (2.48,3) | 1642  (568,2738) | 5.78  (1.98,9.74) | 9005  (3668,15070) | 12.88  (5.16,21.38) | 2.66  (2.41,2.91) |
| Palau | 0  (0,0) | 3  (1.17,5.16) | 0  (0,1) | 3.76  (1.53,6.26) | 0.83  (0.77,0.89) | 4  (2,7) | 75.49  (30.43,130.24) | 9  (4,15) | 83.68  (35.64,138.4) | 0.37  (0.32,0.42) |
| Palestine | 10  (4,18) | 2.28  (0.95,4) | 30  (13,47) | 2.46  (1.04,3.83) | 0.41  (0.27,0.56) | 287  (117,508) | 57.35  (23.3,101.5) | 828  (356,1303) | 58.33  (25.17,90.99) | 0.2  (0.07,0.34) |
| Panama | 7  (3,11) | 0.94  (0.38,1.55) | 38  (17,61) | 1.61  (0.72,2.59) | 1.8  (1.68,1.92) | 157  (64,252) | 20.91  (8.5,33.65) | 851  (385,1363) | 37.49  (16.94,60.05) | 2.02  (1.91,2.13) |
| Papua New Guinea | 3  (1,5) | 0.28  (0.1,0.54) | 9  (3,16) | 0.31  (0.12,0.55) | 0.28  (0.23,0.33) | 93  (34,184) | 8.52  (3.1,16.61) | 305  (117,545) | 9.57  (3.64,17.04) | 0.27  (0.21,0.32) |
| Paraguay | 7  (3,12) | 0.6  (0.25,1.01) | 36  (15,65) | 1.2  (0.5,2.16) | 2.56  (2.38,2.74) | 181  (74,304) | 15.04  (6.14,25.12) | 901  (377,1584) | 28.86  (12.06,50.83) | 2.41  (2.23,2.6) |
| Peru | 41  (17,70) | 0.68  (0.27,1.16) | 192  (81,344) | 1.09  (0.46,1.96) | 1.32  (1.11,1.53) | 1106  (460,1871) | 17.03  (7.08,28.78) | 4631  (2017,8325) | 26.18  (11.37,47.09) | 1.14  (0.93,1.35) |
| Philippines | 50  (18,81) | 0.34  (0.12,0.56) | 306  (121,506) | 0.69  (0.27,1.15) | 2.61  (2.46,2.76) | 1591  (574,2585) | 8.99  (3.19,14.38) | 8972  (3594,15013) | 18.76  (7.51,31.33) | 2.63  (2.48,2.77) |
| Poland | 562  (245,900) | 2.13  (0.93,3.42) | 1117  (481,1829) | 2.43  (1.05,3.99) | 0.11  (-0.07,0.28) | 12313  (5252,19730) | 48.01  (20.49,76.99) | 20856  (9107,34323) | 50.47  (21.93,82.98) | -0.02  (-0.19,0.14) |
| Portugal | 131  (53,218) | 1.66  (0.66,2.76) | 242  (104,406) | 1.51  (0.66,2.47) | -0.28  (-0.48,-0.08) | 2754  (1127,4585) | 36.01  (14.81,60.04) | 4335  (1904,7098) | 33.71  (14.97,54.15) | -0.2  (-0.43,0.04) |
| Puerto Rico | 26  (12,42) | 1.37  (0.6,2.2) | 66  (30,106) | 1.55  (0.7,2.51) | 0.22  (-0.01,0.45) | 602  (272,948) | 31.28  (14.1,49.2) | 1350  (619,2185) | 38.59  (17.6,61.29) | 0.52  (0.3,0.74) |
| Qatar | 1  (0,1) | 2.38  (0.95,4.02) | 6  (3,10) | 2.54  (1.08,4.2) | 0.55  (0.02,1.09) | 25  (10,41) | 55.8  (22.78,93.16) | 187  (83,314) | 54.66  (23.81,91.7) | 0.33  (-0.11,0.77) |
| Republic of Korea | 64  (21,109) | 0.4  (0.13,0.69) | 329  (123,579) | 0.6  (0.23,1.04) | 1.15  (1.01,1.29) | 1746  (581,2950) | 9.69  (3.19,16.38) | 6358  (2481,10880) | 12.56  (4.91,21.27) | 0.71  (0.56,0.87) |
| Republic of Moldova | 50  (22,80) | 1.92  (0.85,3.03) | 74  (33,116) | 2.04  (0.92,3.19) | 0.68  (0.26,1.11) | 1321  (589,2107) | 49.18  (21.94,78.34) | 1763  (787,2770) | 50.62  (22.59,79.57) | 0.55  (0.16,0.94) |
| Romania | 162  (68,260) | 1.06  (0.44,1.7) | 429  (177,728) | 1.92  (0.81,3.28) | 1.53  (1.33,1.73) | 4150  (1759,6755) | 26.98  (11.42,44.04) | 9105  (3837,15438) | 45.92  (19.74,76.85) | 1.31  (1.1,1.51) |
| Russian Federation | 2247  (983,3581) | 1.89  (0.82,3) | 3980  (1724,6214) | 2.56  (1.11,3.98) | 0.73  (0.57,0.89) | 56062  (24632,89610) | 48.81  (21.33,78.2) | 86401  (37682,133154) | 59.52  (25.79,92.13) | 0.31  (0.14,0.48) |
| Rwanda | 5  (2,10) | 0.34  (0.1,0.6) | 15  (5,27) | 0.42  (0.13,0.79) | -0.06  (-0.4,0.28) | 168  (52,308) | 9.33  (2.85,16.9) | 430  (135,806) | 10.57  (3.28,19.34) | -0.46  (-0.84,-0.08) |
| Saint Kitts and Nevis | 0  (0,1) | 1.41  (0.57,2.32) | 1  (0,1) | 1.74  (0.75,2.78) | 1.3  (1.06,1.55) | 7  (3,11) | 34.97  (14.39,57.17) | 14  (6,23) | 38.06  (16.93,59.96) | 0.73  (0.55,0.92) |
| Saint Lucia | 0  (0,1) | 0.9  (0.35,1.46) | 1  (1,2) | 1.12  (0.5,1.85) | 0.11  (-0.15,0.37) | 11  (4,18) | 23.08  (9.23,36.62) | 34  (15,56) | 27.2  (12.08,44.26) | 0.18  (-0.03,0.39) |
| Saint Vincent and the Grenadines | 0  (0,0) | 0.76  (0.3,1.25) | 1  (0,1) | 1.26  (0.53,2.06) | 1.63  (1.44,1.82) | 7  (3,12) | 18.6  (7.28,30.37) | 22  (10,35) | 31.28  (14.1,50.56) | 1.6  (1.44,1.77) |
| Samoa | 1  (0,1) | 1.34  (0.58,2.18) | 1  (1,2) | 1.81  (0.81,2.97) | 0.96  (0.9,1.03) | 17  (7,27) | 36.47  (15.83,58.29) | 38  (17,63) | 49.19  (22.26,81.01) | 0.97  (0.9,1.05) |
| San Marino | 0  (0,1) | 1.85  (0.72,3.16) | 1  (0,1) | 1.08  (0.43,1.97) | -1.13  (-1.37,-0.88) | 7  (3,12) | 38.48  (15.56,63.7) | 9  (4,16) | 23.46  (9.14,41.53) | -0.88  (-1.13,-0.62) |
| Sao Tome and Principe | 0  (0,0) | 0.33  (0.13,0.53) | 0  (0,1) | 0.57  (0.24,0.98) | 1.75  (1.64,1.86) | 3  (1,5) | 7.94  (3.19,13.09) | 8  (4,14) | 13.01  (5.68,21.69) | 1.4  (1.24,1.56) |
| Saudi Arabia | 14  (6,25) | 0.58  (0.23,1) | 94  (41,152) | 1.15  (0.52,1.83) | 2.54  (2.09,3) | 453  (177,782) | 15.59  (6.14,26.8) | 3420  (1486,5497) | 30.71  (13.64,49.08) | 2.53  (2.1,2.96) |
| Senegal | 5  (2,8) | 0.29  (0.11,0.5) | 19  (7,33) | 0.49  (0.19,0.84) | 1.85  (1.71,1.99) | 139  (54,238) | 7.84  (2.96,13.37) | 539  (193,948) | 12.21  (4.45,21.36) | 1.59  (1.44,1.74) |
| Serbia | 123  (54,210) | 2.19  (0.95,3.73) | 231  (102,379) | 2.43  (1.08,4.01) | -0.02  (-0.19,0.14) | 3040  (1328,5136) | 50.18  (21.67,85) | 4914  (2213,8069) | 56.37  (25.36,93.45) | 0.08  (-0.1,0.26) |
| Seychelles | 0  (0,1) | 1.29  (0.49,2.02) | 1  (1,2) | 2.35  (1.06,3.77) | 2.22  (2,2.45) | 11  (4,17) | 36.52  (14.57,57.56) | 37  (17,60) | 60.59  (28.29,96.36) | 1.91  (1.71,2.11) |
| Sierra Leone | 2  (1,4) | 0.21  (0.07,0.39) | 7  (3,13) | 0.39  (0.14,0.71) | 2.41  (2.27,2.55) | 51  (17,97) | 5.07  (1.69,9.53) | 194  (70,350) | 9.52  (3.44,17.19) | 2.41  (2.26,2.56) |
| Singapore | 7  (3,12) | 0.63  (0.22,1.04) | 37  (15,61) | 0.83  (0.34,1.35) | 0.67  (0.47,0.87) | 210  (78,344) | 16.32  (5.95,26.81) | 867  (357,1409) | 19.85  (8.21,32.35) | 0.42  (0.22,0.62) |
| Slovakia | 92  (39,156) | 2.62  (1.11,4.39) | 149  (66,247) | 2.57  (1.14,4.29) | -0.17  (-0.24,-0.1) | 2136  (904,3596) | 62.45  (26.62,104.74) | 3143  (1389,5252) | 58.51  (25.87,98.07) | -0.31  (-0.39,-0.24) |
| Slovenia | 31  (13,49) | 2  (0.86,3.21) | 44  (19,73) | 1.5  (0.65,2.49) | -1.07  (-1.36,-0.78) | 660  (287,1056) | 44.78  (19.51,71.84) | 761  (331,1266) | 30.8  (13.6,51.61) | -1.4  (-1.7,-1.09) |
| Solomon Islands | 0  (0,1) | 0.71  (0.26,1.3) | 2  (1,3) | 0.95  (0.34,1.75) | 0.98  (0.81,1.14) | 15  (6,29) | 20.53  (7.41,38.39) | 59  (22,109) | 27.8  (10.03,50.79) | 1.02  (0.84,1.2) |
| Somalia | 5  (2,10) | 0.35  (0.1,0.7) | 19  (5,36) | 0.49  (0.13,0.91) | 1.13  (1.08,1.17) | 184  (56,371) | 10.59  (3.18,20.86) | 639  (184,1258) | 14.22  (4.01,27.63) | 0.99  (0.94,1.05) |
| South Africa | 105  (44,176) | 0.91  (0.38,1.54) | 391  (171,603) | 1.51  (0.66,2.34) | 1.8  (1.62,1.98) | 2853  (1205,4706) | 22.58  (9.54,37.68) | 10153  (4410,15572) | 36.67  (15.95,56.39) | 1.91  (1.74,2.08) |
| South Sudan | 2  (1,5) | 0.22  (0.07,0.43) | 6  (2,10) | 0.3  (0.09,0.52) | 1  (0.8,1.19) | 66  (21,137) | 5.8  (1.87,11.95) | 179  (59,325) | 7.85  (2.63,14.12) | 0.94  (0.7,1.17) |
| Spain | 558  (230,924) | 1.73  (0.72,2.86) | 1008  (429,1661) | 1.57  (0.68,2.57) | -0.37  (-0.48,-0.27) | 11511  (4855,19048) | 38.41  (16.26,63.51) | 18097  (7947,29813) | 34.77  (15.22,56.81) | -0.4  (-0.51,-0.29) |
| Sri Lanka | 10  (4,16) | 0.19  (0.07,0.32) | 43  (17,77) | 0.29  (0.12,0.51) | 1.67  (1.5,1.85) | 287  (107,470) | 4.83  (1.82,8.04) | 1083  (435,1903) | 7.19  (2.85,12.7) | 1.56  (1.36,1.75) |
| Sudan | 31  (11,60) | 0.68  (0.25,1.3) | 97  (38,180) | 1.04  (0.41,1.85) | 1.42  (1.34,1.5) | 940  (313,1871) | 18.74  (6.32,36.92) | 3131  (1171,6012) | 27.81  (11,51.68) | 1.33  (1.25,1.41) |
| Suriname | 1  (0,2) | 0.76  (0.28,1.26) | 4  (2,7) | 1.14  (0.47,1.91) | 1.64  (1.44,1.85) | 27  (10,45) | 19.32  (7.29,32.26) | 100  (43,167) | 29.01  (12.3,48.07) | 1.54  (1.34,1.74) |
| Sweden | 136  (57,228) | 1.49  (0.63,2.5) | 187  (79,312) | 1.41  (0.6,2.34) | -0.14  (-0.28,-0.01) | 2639  (1109,4376) | 33.73  (14.35,55.68) | 3233  (1379,5417) | 29.27  (12.3,49.21) | -0.29  (-0.43,-0.15) |
| Switzerland | 68  (28,110) | 1.02  (0.43,1.66) | 95  (38,155) | 0.82  (0.34,1.34) | -0.73  (-0.85,-0.6) | 1319  (554,2114) | 22.63  (9.43,36.19) | 1681  (690,2769) | 17.53  (7.34,28.96) | -0.85  (-0.98,-0.73) |
| Syrian Arab Republic | 20  (8,33) | 0.83  (0.33,1.37) | 69  (29,114) | 1.22  (0.51,2.01) | 1.08  (0.93,1.23) | 606  (251,999) | 21.48  (8.81,35.11) | 1920  (803,3216) | 28.31  (11.85,46.99) | 0.73  (0.54,0.92) |
| Taiwan (Province of China) | 51  (20,80) | 0.71  (0.28,1.12) | 351  (150,567) | 1.5  (0.64,2.44) | 2.03  (1.66,2.41) | 1365  (527,2192) | 17.39  (6.74,27.75) | 7491  (3218,12152) | 34.56  (14.86,55.76) | 1.87  (1.57,2.17) |
| Tajikistan | 11  (4,17) | 0.7  (0.29,1.13) | 15  (6,26) | 0.52  (0.21,0.87) | -1.03  (-1.22,-0.85) | 301  (124,493) | 19.24  (7.92,31.41) | 465  (185,829) | 13.56  (5.34,23.39) | -1.24  (-1.38,-1.09) |
| Thailand | 84  (31,145) | 0.43  (0.16,0.76) | 574  (225,981) | 0.97  (0.38,1.64) | 2.42  (2.24,2.61) | 2556  (968,4357) | 11.95  (4.5,20.55) | 14620  (5980,24350) | 25.74  (10.61,43.01) | 2.27  (2.05,2.49) |
| Timor-Leste | 0  (0,0) | 0.11  (0.02,0.22) | 1  (0,2) | 0.19  (0.06,0.36) | 1.82  (1.54,2.11) | 6  (1,11) | 3.1  (0.72,6.16) | 26  (8,47) | 5.58  (1.72,10.09) | 1.94  (1.62,2.27) |
| Togo | 2  (1,3) | 0.26  (0.1,0.43) | 10  (3,17) | 0.48  (0.16,0.84) | 2.26  (2.16,2.37) | 48  (19,81) | 6.52  (2.57,10.98) | 276  (99,501) | 11.49  (4.01,20.63) | 2.05  (1.94,2.15) |
| Tokelau | 0  (0,0) | 1.13  (0.45,1.99) | 0  (0,0) | 1.4  (0.64,2.3) | 0.65  (0.63,0.67) | 0  (0,0) | 31.33  (13.11,54.37) | 0  (0,0) | 38.13  (17.46,62.97) | 0.54  (0.5,0.58) |
| Tonga | 0  (0,1) | 1.14  (0.46,1.81) | 1  (0,1) | 1.38  (0.64,2.26) | 0.63  (0.57,0.69) | 10  (4,15) | 31.18  (12.98,49.56) | 15  (7,25) | 34.65  (16.05,56.76) | 0.27  (0.2,0.33) |
| Trinidad and Tobago | 6  (3,9) | 1.4  (0.6,2.19) | 18  (7,29) | 1.72  (0.73,2.82) | 0.5  (0.37,0.64) | 152  (66,238) | 34.19  (14.85,53.55) | 439  (189,726) | 44.33  (19.26,73.49) | 0.66  (0.51,0.8) |
| Tunisia | 15  (6,26) | 0.63  (0.25,1.09) | 60  (25,110) | 0.88  (0.37,1.62) | 1.04  (0.99,1.09) | 413  (178,710) | 15.91  (6.72,27.53) | 1537  (666,2767) | 21.85  (9.42,39.33) | 0.99  (0.95,1.03) |
| Turkey | 323  (136,526) | 1.8  (0.76,2.95) | 822  (359,1338) | 1.68  (0.73,2.73) | -0.26  (-0.67,0.15) | 9129  (3743,14995) | 46.64  (19.23,76) | 19706  (8574,31872) | 39.37  (17.11,63.68) | -0.73  (-1.09,-0.37) |
| Turkmenistan | 6  (3,11) | 0.57  (0.23,0.94) | 12  (5,20) | 0.55  (0.22,0.9) | -0.34  (-0.86,0.18) | 182  (74,300) | 15.64  (6.38,25.81) | 354  (143,586) | 14.83  (6.03,24.56) | -0.36  (-0.87,0.15) |
| Tuvalu | 0  (0,0) | 1.07  (0.41,1.88) | 0  (0,0) | 1.45  (0.61,2.41) | 0.98  (0.94,1.02) | 1  (0,2) | 30.26  (11.77,53.86) | 2  (1,4) | 38.72  (16,65.34) | 0.79  (0.74,0.84) |
| Uganda | 10  (3,17) | 0.29  (0.09,0.53) | 43  (16,76) | 0.51  (0.18,0.9) | 1.26  (0.99,1.54) | 284  (95,515) | 7.83  (2.58,14.06) | 1327  (497,2363) | 13.77  (5.02,24.52) | 1.21  (0.91,1.51) |
| Ukraine | 994  (421,1555) | 2.12  (0.91,3.32) | 935  (394,1667) | 1.91  (0.8,3.43) | -0.55  (-0.67,-0.42) | 24449  (10416,38314) | 54.99  (23.52,86.15) | 22200  (9305,39967) | 49.02  (20.41,88.99) | -0.65  (-0.79,-0.52) |
| United Arab Emirates | 4  (1,7) | 2.32  (0.85,4.21) | 26  (11,45) | 6.06  (2.53,10.26) | 4.84  (4.18,5.51) | 122  (44,226) | 62.75  (23.17,113.79) | 854  (354,1434) | 115.25(48.94,195.32) | 3.21  (2.72,3.71) |
| United Kingdom | 1211  (520,1993) | 2.16  (0.93,3.54) | 1362  (589,2242) | 1.74  (0.76,2.85) | -0.76  (-0.89,-0.62) | 23910  (10280,38944) | 48.28  (20.74,78.2) | 24884  (10880,40692) | 37.49  (16.45,61.14) | -0.86  (-1,-0.72) |
| United Republic of Tanzania | 25(10,42) | 0.46  (0.18,0.77) | 104  (40,179) | 0.81  (0.31,1.39) | 2.04  (1.95,2.13) | 737  (275,1230) | 12.09  (4.59,20.28) | 2925  (1133,5082) | 19.96  (7.83,34.54) | 1.8  (1.7,1.89) |
| United States of America | 3839  (1622,6187) | 1.98  (0.84,3.18) | 5330  (2338,8268) | 1.63  (0.73,2.51) | -0.86  (-0.98,-0.75) | 81704  (35042,130126) | 46.57  (20.09,73.9) | 117037  (53259,178460) | 40.87  (18.79,62.48) | -0.58  (-0.67,-0.49) |
| United States Virgin Islands | 1  (0,2) | 2.59  (1.09,4.19) | 2  (1,3) | 1.52  (0.63,2.69) | -1.57  (-1.8,-1.34) | 28  (12,44) | 58.7  (25.17,94.05) | 32  (13,57) | 36.34  (15.24,64.72) | -1.37  (-1.57,-1.17) |
| Uruguay | 57  (24,96) | 2.52  (1.05,4.18) | 106  (46,174) | 2.94  (1.3,4.85) | 0.37  (0.27,0.47) | 1207  (504,1993) | 56.24  (23.59,92.43) | 1980  (878,3257) | 65.28  (29.04,107.58) | 0.39  (0.3,0.49) |
| Uzbekistan | 44  (18,73) | 0.67  (0.28,1.11) | 88  (35,147) | 0.6  (0.24,1.01) | -0.32  (-0.72,0.07) | 1227  (518,1985) | 18.34  (7.75,29.94) | 2628  (1072,4376) | 16.54  (6.71,27.62) | -0.39  (-0.77,-0.01) |
| Vanuatu | 0  (0,0) | 0.76  (0.28,1.48) | 1  (0,2) | 1.02  (0.4,1.88) | 0.81  (0.71,0.91) | 7  (2,13) | 20.34  (7.5,39.34) | 27  (11,51) | 27.08  (10.63,50.26) | 0.75  (0.62,0.88) |
| Venezuela (Bolivarian Republic of) | 50  (21,81) | 1.01  (0.43,1.65) | 235  (102,407) | 1.46  (0.63,2.53) | 1.21  (1.1,1.32) | 1340  (570,2153) | 24.59  (10.48,39.68) | 6016  (2604,10394) | 36.84  (15.92,63.71) | 1.28  (1.14,1.43) |
| Viet Nam | 18  (4,33) | 0.08  (0.02,0.14) | 148  (51,266) | 0.26  (0.09,0.46) | 4.54  (4.35,4.74) | 511  (127,934) | 2.17  (0.55,3.95) | 4162  (1392,7569) | 7.04  (2.38,12.72) | 4.54  (4.3,4.77) |
| Yemen | 10  (3,18) | 0.38  (0.13,0.7) | 42  (14,77) | 0.59  (0.19,1.07) | 1.43  (1.29,1.58) | 297  (104,582) | 10.54  (3.76,20.35) | 1309  (421,2404) | 15.49  (5,28.09) | 1.23  (1.1,1.36) |
| Zambia | 5  (2,9) | 0.38  (0.14,0.65) | 31  (11,65) | 0.87  (0.31,1.77) | 2.56  (2.41,2.72) | 165  (63,290) | 10.23  (3.79,17.84) | 958  (332,2117) | 22.6  (8.11,47.86) | 2.42  (2.26,2.59) |
| Zimbabwe | 12  (4,19) | 0.57  (0.22,0.93) | 59  (24,102) | 1.51  (0.62,2.54) | 4.24  (3.52,4.96) | 328  (130,540) | 14.56  (5.69,23.76) | 1781  (724,3116) | 39.23  (15.95,68.21) | 4.38  (3.57,5.2) |

BMI, body mass index; DALYs, disability-adjusted life years; ASMR, age-standardized mortality rate; ASDR, age-standardized DALY rate; EAPC, estimated annual percentage change; UI, uncertainty interval;

CI, confidence interval.

**Table S6** Deaths and DALYs for ovarian cancer attributable to high BMI in 1990 and 2021, with corresponding EAPC from 1990 to 2021, in 204 countries

|  | **Deaths** | | | | | **DALYs** | | | | |
| --- | --- | --- | --- | --- | --- | --- | --- | --- | --- | --- |
| **countries** | **Number of cases (95% UI), 1990** | **ASMR per 100,000 (95% UI), 1990** | **Number of cases (95% UI), 2021** | **ASMR per 100,000 (95% UI), 2021** | **EAPC of ASMR (95% CI),1990-2021** | **Number of**  **cases (95% UI), 1990** | **ASDR per 100,000 (95% UI), 1990** | **Number of**  **cases (95% UI),**  **2021** | **ASDR per 100,000 (95% UI), 2021** | **EAPC of ASDR (95% CI),**  **1990-2021** |
| Afghanistan | 5  (1,16) | 0.13  (0.02,0.41) | 15  (3,39) | 0.23  (0.04,0.61) | 1.93  (1.69,2.18) | 165  (22,531) | 4.28  (0.55,13.79) | 556  (104,1523) | 7.49  (1.45,19.39) | 1.93  (1.68,2.18) |
| Albania | 2  (0,4) | 0.19  (0.04,0.37) | 7  (2,13) | 0.29  (0.07,0.57) | 1.73  (1.57,1.89) | 57  (12,115) | 5.17  (1.07,10.4) | 167  (40,327) | 7.78  (1.85,15.22) | 1.71  (1.54,1.89) |
| Algeria | 7  (1,15) | 0.12  (0.02,0.24) | 44  (12,82) | 0.25  (0.07,0.47) | 2.66  (2.56,2.77) | 229  (48,459) | 3.35  (0.68,6.7) | 1356  (356,2454) | 6.86  (1.81,12.54) | 2.35  (2.28,2.42) |
| American Samoa | 0  (0,0) | 0.26  (0.07,0.49) | 0  (0,0) | 0.56  (0.16,1.04) | 2.6  (2.52,2.68) | 1  (0,2) | 8.73  (2.24,16.36) | 5  (1,9) | 18.19  (5.36,33.95) | 2.52  (2.44,2.6) |
| Andorra | 0  (0,0) | 0.19  (0.03,0.41) | 0  (0,0) | 0.18  (0.04,0.36) | 0.14  (0,0.29) | 1  (0,3) | 5  (0.82,11.07) | 3  (1,7) | 4.71  (1.02,9.76) | 0.12  (-0.03,0.26) |
| Angola | 1  (0,2) | 0.03  (0,0.09) | 12  (2,27) | 0.16  (0.02,0.34) | 5.42  (5.25,5.6) | 27  (0,71) | 1.07  (-0.02,2.78) | 434  (66,936) | 5.02  (0.75,10.83) | 5.32  (5.14,5.5) |
| Antigua and Barbuda | 0  (0,0) | 0.37  (0.07,0.68) | 0  (0,1) | 0.84  (0.2,1.51) | 2.93  (2.75,3.11) | 3  (1,6) | 11.4  (2.34,21.5) | 14  (4,26) | 24.19  (5.92,44.27) | 2.72  (2.55,2.89) |
| Argentina | 115  (27,216) | 0.64  (0.15,1.21) | 214  (57,385) | 0.71  (0.19,1.27) | 0.64  (0.47,0.81) | 3191  (727,6046) | 18.2  (4.13,34.54) | 5757  (1552,10230) | 20.15  (5.44,35.71) | 0.64  (0.47,0.81) |
| Armenia | 7  (2,14) | 0.47  (0.11,0.87) | 18  (5,33) | 0.73  (0.2,1.31) | 1.57  (1.38,1.75) | 222  (52,423) | 13.69  (3.21,26.03) | 488  (136,879) | 20.19  (5.51,36.53) | 1.44  (1.23,1.65) |
| Australia | 91  (19,172) | 0.87  (0.19,1.63) | 151  (39,274) | 0.63  (0.17,1.13) | -0.95  (-1.36,-0.53) | 2409  (512,4515) | 24.26  (5.12,45.55) | 3397  (908,6081) | 16.08  (4.28,28.7) | -1.24  (-1.65,-0.83) |
| Austria | 51  (10,98) | 0.72  (0.14,1.37) | 51  (11,102) | 0.51  (0.11,0.98) | -0.98  (-1.08,-0.88) | 1176  (232,2217) | 18.49  (3.53,35.23) | 1077  (234,2046) | 12.24  (2.65,23.1) | -1.12  (-1.24,-0.99) |
| Azerbaijan | 7  (1,13) | 0.22  (0.04,0.45) | 24  (6,49) | 0.38  (0.09,0.77) | 2.17  (1.78,2.56) | 218  (46,442) | 7.18  (1.47,14.55) | 782  (184,1640) | 11.96  (2.79,24.8) | 2  (1.61,2.39) |
| Bahamas | 1  (0,1) | 0.68  (0.16,1.33) | 3  (1,5) | 1.13  (0.3,2.07) | 1.86  (1.75,1.96) | 22  (5,42) | 22.17  (5.14,43.3) | 86  (23,157) | 35.8  (9.6,65.14) | 1.75  (1.63,1.87) |
| Bahrain | 1  (0,1) | 0.73  (0.16,1.45) | 5  (2,10) | 1.35  (0.41,2.54) | 1.66  (1.55,1.78) | 22  (5,43) | 21.03  (4.82,41.91) | 176  (55,324) | 36.75  (11.28,67.96) | 1.51  (1.39,1.63) |
| Bangladesh | 2  (-3,8) | 0.01  (-0.01,0.03) | 80  (8,180) | 0.11  (0.01,0.24) | 9.59  (9.2,9.99) | 68  (-90,284) | 0.26  (-0.37,1.12) | 2851  (330,6399) | 3.65  (0.41,8.22) | 9.59  (9.21,9.97) |
| Barbados | 1  (0,2) | 0.59  (0.13,1.16) | 3  (1,5) | 0.93  (0.26,1.71) | 1.88  (1.72,2.04) | 25  (5,49) | 18.4  (4.02,35.32) | 69  (20,125) | 27.53  (8.23,49.29) | 1.67  (1.53,1.82) |
| Belarus | 40  (9,75) | 0.49  (0.11,0.93) | 72  (17,137) | 0.77  (0.18,1.45) | 1.18  (0.62,1.75) | 1145  (259,2162) | 14.74  (3.31,27.93) | 1988  (471,3794) | 22.8  (5.31,42.73) | 1.17  (0.6,1.74) |
| Belgium | 55  (11,104) | 0.65  (0.13,1.22) | 69  (14,126) | 0.54  (0.12,0.99) | -0.68  (-0.8,-0.55) | 1341  (263,2509) | 17.42  (3.44,32.57) | 1454  (312,2631) | 13.54  (2.96,24.34) | -0.94  (-1.07,-0.81) |
| Belize | 0  (0,0) | 0.21  (0.05,0.38) | 1  (0,1) | 0.4  (0.12,0.7) | 2.18  (1.79,2.57) | 3  (1,6) | 6.72  (1.63,11.78) | 23  (7,39) | 12.7  (3.77,21.65) | 2.15  (1.79,2.51) |
| Benin | 1  (0,2) | 0.1  (0.02,0.2) | 5  (1,10) | 0.17  (0.04,0.32) | 1.59  (1.52,1.66) | 35  (7,70) | 3.11  (0.61,6.15) | 166  (37,328) | 5.08  (1.16,10.09) | 1.42  (1.35,1.49) |
| Bermuda | 0  (0,1) | 0.87  (0.18,1.7) | 1  (0,1) | 0.98  (0.28,1.78) | 0.05  (-0.17,0.27) | 9  (2,17) | 24.8  (5.17,47.57) | 17  (5,30) | 27.35  (8.23,49.1) | -0.04  (-0.25,0.18) |
| Bhutan | 0  (0,0) | 0.12  (0.01,0.28) | 1  (0,2) | 0.27  (0.05,0.63) | 2.67  (2.62,2.72) | 6  (1,14) | 4.01  (0.48,9.29) | 28  (5,68) | 8.74  (1.6,20.81) | 2.57  (2.51,2.62) |
| Bolivia (Plurinational State of) | 3  (0,7) | 0.14  (0.02,0.36) | 24  (6,48) | 0.47  (0.12,0.94) | 4.02  (3.78,4.26) | 88  (12,215) | 4.52  (0.6,11.1) | 761  (186,1522) | 14.42  (3.54,28.76) | 3.88  (3.65,4.12) |
| Bosnia and Herzegovina | 10  (2,19) | 0.41  (0.09,0.78) | 24  (6,44) | 0.72  (0.18,1.32) | 2.13  (1.94,2.33) | 309  (68,583) | 12.24  (2.66,23) | 628  (159,1153) | 20.3  (5.17,37.34) | 1.97  (1.76,2.18) |
| Botswana | 1  (0,2) | 0.25  (0.05,0.53) | 5  (1,10) | 0.59  (0.14,1.1) | 3.02  (2.81,3.22) | 25  (5,57) | 7.2  (1.59,16.01) | 157  (36,308) | 16.4  (3.78,31.67) | 2.83  (2.64,3.03) |
| Brazil | 143  (30,278) | 0.29  (0.06,0.55) | 634  (148,1170) | 0.46  (0.11,0.85) | 1.21  (1.08,1.34) | 4565  (961,8891) | 8.61  (1.8,16.73) | 18672  (4399,34138) | 13.58  (3.2,24.81) | 1.15  (1.04,1.26) |
| Brunei Darussalam | 0  (0,0) | 0.21  (0.01,0.47) | 1  (0,2) | 0.56  (0.13,1) | 3.48  (3.16,3.81) | 5  (0,11) | 7.54  (0.62,16.5) | 45  (11,81) | 19.17  (4.56,34.83) | 3.34  (3.04,3.65) |
| Bulgaria | 38  (9,68) | 0.58  (0.14,1.05) | 71  (16,136) | 0.98  (0.23,1.86) | 1.52  (1.23,1.81) | 1120  (270,2014) | 17.71  (4.22,31.77) | 1850  (437,3498) | 29.08  (7.02,55.46) | 1.43  (1.17,1.7) |
| Burkina Faso | 0  (0,1) | 0.01  (-0.01,0.03) | 1  (0,4) | 0.02  (-0.01,0.06) | 4.09  (3.8,4.39) | 8  (-6,27) | 0.28  (-0.26,0.96) | 53  (-6,139) | 0.82  (-0.15,2.21) | 3.57  (3.3,3.84) |
| Burundi | 1  (0,2) | 0.04  (-0.02,0.12) | 3  (0,6) | 0.11  (0.01,0.25) | 2.69  (2.47,2.92) | 17  (-7,53) | 1.27  (-0.46,3.81) | 89  (5,208) | 3.12  (0.18,7.32) | 2.64  (2.41,2.88) |
| Cabo Verde | 0  (0,0) | 0.03  (0.01,0.06) | 1  (0,1) | 0.2  (0.04,0.41) | 6.56  (5.79,7.34) | 1  (0,2) | 0.97  (0.18,2.02) | 16  (4,32) | 5.95  (1.33,12.28) | 6.12  (5.38,6.87) |
| Cambodia | 1  (0,2) | 0.03  (-0.01,0.08) | 10  (0,24) | 0.13  (0,0.3) | 5.46  (5.41,5.51) | 28  (-10,84) | 0.89  (-0.32,2.66) | 359  (12,837) | 4.39  (0.12,10.25) | 5.38  (5.33,5.43) |
| Cameroon | 4  (1,7) | 0.15  (0.03,0.3) | 21  (5,43) | 0.3  (0.07,0.61) | 2.26  (2.18,2.34) | 120  (25,238) | 4.44  (0.94,8.89) | 700  (161,1393) | 8.78  (2.01,17.74) | 2.15  (2.06,2.24) |
| Canada | 110  (25,204) | 0.62  (0.14,1.16) | 237  (61,426) | 0.62  (0.16,1.12) | 0.09  (-0.13,0.31) | 2869  (656,5386) | 17.21  (3.92,32.32) | 5430  (1415,9810) | 16.04  (4.19,28.76) | -0.2  (-0.39,-0.02) |
| Central African Republic | 0  (0,1) | 0.05  (0,0.11) | 2  (0,4) | 0.14  (0.02,0.3) | 3.78  (3.66,3.91) | 12  (0,28) | 1.48  (0.03,3.68) | 71  (13,153) | 4.43  (0.78,9.57) | 3.68  (3.55,3.81) |
| Chad | 1  (0,1) | 0.04  (0.01,0.09) | 3  (0,6) | 0.09  (0.01,0.2) | 2.63  (2.59,2.66) | 19  (3,41) | 1.22  (0.18,2.63) | 82  (12,180) | 2.63  (0.41,5.78) | 2.55  (2.51,2.59) |
| Chile | 25  (5,46) | 0.44  (0.1,0.82) | 84  (21,146) | 0.61  (0.16,1.06) | 0.95  (0.62,1.28) | 725  (160,1356) | 12.76  (2.82,23.85) | 2322  (608,4029) | 17.72  (4.65,30.7) | 0.94  (0.61,1.27) |
| China | 139(-68,380) | 0.03(-0.02,0.08) | 1744(342,3601) | 0.16  (0.03,0.32) | 5.02  (4.86,5.18) | 4569  (-2299,12584) | 0.98  (-0.47,2.67) | 52980  (10497,108333) | 4.85  (0.96,9.92) | 4.96  (4.82,5.1) |
| Colombia | 31  (6,61) | 0.33  (0.06,0.64) | 177  (43,324) | 0.59  (0.14,1.07) | 1.96  (1.81,2.11) | 986  (182,1914) | 9.63  (1.86,18.75) | 5256  (1250,9769) | 17.7  (4.19,32.81) | 2.09  (1.92,2.26) |
| Comoros | 0  (0,0) | 0.09  (0,0.23) | 1  (0,2) | 0.36  (0.06,0.78) | 4.26  (4.15,4.37) | 4  (0,8) | 2.88  (-0.09,6.96) | 34  (6,73) | 11.05  (1.83,23.83) | 4.27  (4.14,4.4) |
| Congo | 1  (0,2) | 0.1  (0.01,0.23) | 6  (1,12) | 0.38  (0.08,0.74) | 4.44  (4.3,4.59) | 22  (2,53) | 3.17  (0.27,7.56) | 215  (45,427) | 11.82  (2.47,22.94) | 4.22  (4.05,4.4) |
| Cook Islands | 0  (0,0) | 0.15  (0.03,0.29) | 0  (0,0) | 0.25  (0.07,0.44) | 1.73  (1.61,1.84) | 0  (0,1) | 4.88  (1.19,9.17) | 1  (0,2) | 7.91  (2.3,14) | 1.66  (1.53,1.78) |
| Costa Rica | 2  (0,4) | 0.2  (0.04,0.39) | 14  (3,25) | 0.46  (0.11,0.83) | 2.44  (2.11,2.77) | 59  (13,112) | 6.04  (1.32,11.58) | 419  (104,748) | 14.29  (3.53,25.48) | 2.53  (2.24,2.83) |
| Croatia | 26  (6,49) | 0.71  (0.16,1.34) | 44  (11,79) | 0.9  (0.23,1.61) | 0.99  (0.77,1.22) | 701  (157,1292) | 19.49  (4.39,35.96) | 1025  (257,1828) | 24.1  (5.94,43.4) | 0.86  (0.62,1.1) |
| Cuba | 10  (2,19) | 0.19  (0.04,0.37) | 46  (11,86) | 0.47  (0.11,0.87) | 2.51  (2.29,2.74) | 312  (67,593) | 6.04  (1.3,11.51) | 1355  (324,2511) | 14.64  (3.53,27.15) | 2.38  (2.14,2.62) |
| Cyprus | 2  (0,3) | 0.37  (0.06,0.78) | 6  (1,12) | 0.56  (0.12,1.09) | 1.49  (1.21,1.77) | 42  (8,87) | 9.58  (1.71,19.8) | 146  (32,285) | 14.18  (3.16,27.6) | 1.39  (1.11,1.67) |
| Czechia | 63  (14,119) | 0.8  (0.18,1.51) | 100  (23,190) | 0.86  (0.21,1.63) | 0.36  (0.07,0.65) | 1687  (380,3187) | 22.91  (5.14,43.03) | 2331  (555,4423) | 22.69  (5.47,42.86) | 0.09  (-0.19,0.37) |
| Côte d'Ivoire | 3  (1,6) | 0.15  (0.03,0.29) | 18  (4,37) | 0.31  (0.06,0.61) | 2.43  (2.37,2.5) | 113  (21,222) | 4.58  (0.88,8.98) | 630  (124,1260) | 9.22  (1.78,18.67) | 2.33  (2.26,2.41) |
| Democratic People's Republic of Korea | 0  (-2,3) | 0.01  (-0.02,0.03) | 14  (1,34) | 0.07  (0,0.18) | 8.78  (8.65,8.92) | 9  (-70,104) | 0.1  (-0.64,0.99) | 308  (-37,844) | 1.6  (-0.23,4.49) | 9.52  (9.39,9.65) |
| Democratic Republic of the Congo | 3  (0,8) | 0.04  (0,0.09) | 36  (6,77) | 0.17  (0.03,0.36) | 5.14  (4.89,5.39) | 105  (-10,264) | 1.05  (-0.09,2.63) | 1173  (203,2490) | 4.93  (0.81,10.45) | 5.22  (4.96,5.48) |
| Denmark | 22  (4,41) | 0.53  (0.1,1) | 37  (8,68) | 0.59  (0.13,1.08) | -0.37  (-0.88,0.16) | 563  (103,1064) | 14.96  (2.77,28.67) | 808  (174,1446) | 14.59  (3.12,25.87) | -0.81  (-1.34,-0.28) |
| Djibouti | 0  (0,0) | 0.03  (-0.02,0.11) | 0  (0,1) | 0.12  (0,0.3) | 4.09  (3.98,4.2) | 1  (-1,3) | 1.14  (-0.61,3.48) | 16  (1,39) | 4  (0.14,9.59) | 3.93  (3.82,4.04) |
| Dominica | 0  (0,0) | 0.37  (0.08,0.71) | 0  (0,0) | 0.54  (0.15,0.99) | 1.31  (1.18,1.43) | 3  (1,7) | 10.17  (2.22,19.47) | 7  (2,12) | 15.61  (4.28,29.11) | 1.5  (1.41,1.59) |
| Dominican Republic | 2  (0,4) | 0.08  (0.01,0.18) | 12  (2,23) | 0.23  (0.04,0.44) | 3.56  (3.37,3.75) | 66  (10,138) | 2.88  (0.44,6.11) | 401  (85,769) | 7.46  (1.58,14.29) | 3.37  (3.18,3.56) |
| Ecuador | 1  (0,3) | 0.05  (0.01,0.09) | 50  (12,91) | 0.58  (0.14,1.05) | 8.43  (6.57,10.33) | 49  (10,91) | 1.57  (0.32,2.94) | 1571  (384,2810) | 17.81  (4.36,31.92) | 8.29  (6.4,10.22) |
| Egypt | 22  (5,59) | 0.15  (0.04,0.44) | 184  (55,324) | 0.59  (0.18,1.04) | 5.23  (4.62,5.84) | 738  (178,1946) | 4.46  (1.08,12.19) | 5953  (1831,10519) | 16.45  (4.98,28.88) | 4.91  (4.36,5.48) |
| El Salvador | 4  (1,7) | 0.23  (0.05,0.45) | 20  (5,38) | 0.58  (0.15,1.09) | 3.22  (2.99,3.46) | 124  (28,243) | 7.2  (1.61,14.15) | 633  (167,1191) | 18.13  (4.79,34.11) | 3.18  (2.95,3.41) |
| Equatorial Guinea | 0  (0,0) | 0.09  (0.01,0.2) | 1  (0,2) | 0.36  (0.07,0.73) | 4.69  (4.57,4.81) | 4  (1,8) | 2.89  (0.38,6.43) | 43  (8,87) | 11.11  (2.13,22.48) | 4.51  (4.38,4.64) |
| Eritrea | 0  (0,1) | 0.04  (0,0.1) | 2  (0,5) | 0.13  (0.01,0.29) | 3.66  (3.57,3.74) | 11  (-1,27) | 1.3  (-0.1,3.23) | 81  (6,175) | 4.06  (0.29,8.86) | 3.55  (3.46,3.64) |
| Estonia | 11  (2,19) | 0.83  (0.19,1.54) | 12  (3,22) | 0.79  (0.18,1.42) | -0.24  (-1.03,0.57) | 291  (68,537) | 24.47  (5.71,45.23) | 277  (65,501) | 21.08  (4.91,37.97) | -0.62  (-1.38,0.16) |
| Eswatini | 1  (0,2) | 0.56  (0.13,1.22) | 4  (1,8) | 1.11  (0.25,2.25) | 2.25  (2.11,2.39) | 28  (6,60) | 15.43  (3.44,32.96) | 116  (25,241) | 30.72  (6.82,62.88) | 2.27  (2.11,2.43) |
| Ethiopia | 5  (-1,17) | 0.05  (-0.01,0.14) | 23  (0,49) | 0.1  (0,0.21) | 1.92  (1.72,2.13) | 195  (-35,597) | 1.61  (-0.31,4.96) | 802  (16,1689) | 3.06  (0.05,6.42) | 1.68  (1.47,1.88) |
| Fiji | 1  (0,1) | 0.24  (0.06,0.48) | 2  (1,3) | 0.4  (0.12,0.71) | 1.56  (1.46,1.66) | 18  (4,36) | 7.63  (1.82,15.4) | 55  (16,101) | 12.15  (3.65,22.12) | 1.47  (1.39,1.54) |
| Finland | 27  (5,51) | 0.64  (0.12,1.21) | 39  (9,72) | 0.57  (0.13,1.04) | -0.31  (-0.5,-0.12) | 654  (128,1245) | 17.05  (3.29,32.44) | 818  (186,1507) | 14.44  (3.33,26.55) | -0.58  (-0.79,-0.37) |
| France | 196  (33,361) | 0.42  (0.07,0.77) | 394  (91,716) | 0.5  (0.12,0.91) | 0.58  (0.35,0.81) | 4573  (768,8332) | 10.93  (1.82,19.79) | 8067  (1883,14753) | 12.3  (2.83,22.26) | 0.37  (0.11,0.64) |
| Gabon | 1  (0,2) | 0.25  (0.05,0.48) | 3  (1,7) | 0.57  (0.14,1.13) | 2.6  (2.53,2.68) | 25  (5,48) | 7.59  (1.42,14.58) | 112  (29,221) | 17.1  (4.4,33.65) | 2.5  (2.42,2.58) |
| Gambia | 0  (0,0) | 0.08  (0.01,0.16) | 1  (0,2) | 0.18  (0.04,0.38) | 2.26  (2.01,2.5) | 5  (1,9) | 2.56  (0.46,5) | 33  (7,69) | 5.57  (1.23,11.92) | 2.2  (1.93,2.47) |
| Georgia | 7  (2,14) | 0.19  (0.05,0.38) | 34  (8,66) | 1.03  (0.24,1.97) | 6.62  (6.2,7.03) | 220  (54,430) | 6.09  (1.47,11.96) | 921  (214,1776) | 30.01  (6.89,57.51) | 6.32  (5.91,6.73) |
| Germany | 637  (133,1188) | 0.84  (0.17,1.58) | 625  (141,1165) | 0.61  (0.14,1.14) | -1.14  (-1.22,-1.05) | 15194  (3099,28603) | 22.47  (4.53,42.25) | 13440  (3078,25036) | 15.57  (3.61,28.99) | -1.25  (-1.34,-1.17) |
| Ghana | 2  (0,4) | 0.05  (0,0.1) | 24  (5,48) | 0.22  (0.05,0.45) | 5.06  (4.86,5.26) | 69  (8,141) | 1.66  (0.19,3.44) | 816  (184,1687) | 6.96  (1.59,14.12) | 4.68  (4.48,4.88) |
| Greece | 38  (7,73) | 0.46  (0.09,0.9) | 85  (20,157) | 0.7  (0.17,1.29) | 0.91  (0.68,1.13) | 987  (195,1917) | 12.78  (2.52,24.79) | 1889  (455,3473) | 18.63  (4.53,34.01) | 0.89  (0.72,1.07) |
| Greenland | 0  (0,0) | 1.31  (0.32,2.47) | 0  (0,1) | 0.92  (0.23,1.7) | -1.27  (-1.54,-1) | 7  (2,14) | 37.54  (9.06,70.32) | 10  (3,20) | 28.79  (7.15,53.83) | -1.02  (-1.22,-0.82) |
| Grenada | 0  (0,0) | 0.47  (0.09,1.01) | 1  (0,1) | 1.05  (0.26,1.97) | 3.02  (2.85,3.2) | 5  (1,11) | 15.9  (2.94,34.29) | 20  (5,37) | 32.98  (8.57,60.81) | 2.78  (2.61,2.94) |
| Guam | 0  (0,0) | 0.24  (0.05,0.49) | 0  (0,1) | 0.4  (0.1,0.74) | 2.68  (2.26,3.1) | 3  (1,7) | 7.01  (1.5,14.73) | 14  (4,26) | 14.18  (3.66,25.9) | 3.13  (2.74,3.52) |
| Guatemala | 2  (0,4) | 0.11  (0.03,0.2) | 19  (5,35) | 0.31  (0.08,0.57) | 4.06  (3.64,4.48) | 74  (17,136) | 3.34  (0.8,6.21) | 627  (163,1160) | 9.62  (2.51,17.82) | 4.1  (3.66,4.54) |
| Guinea | 1  (0,2) | 0.07  (0.01,0.14) | 4  (1,9) | 0.14  (0.03,0.28) | 2.17  (2.11,2.24) | 39  (7,78) | 2.19  (0.37,4.36) | 144  (29,290) | 4.26  (0.9,8.74) | 2.13  (2.06,2.19) |
| Guinea-Bissau | 0  (0,0) | 0.07  (0.01,0.15) | 1  (0,2) | 0.16  (0.03,0.32) | 2.93  (2.9,2.96) | 5(  1,12) | 2.14  (0.3,4.78) | 26  (5,54) | 5.01  (0.87,10.28) | 2.85  (2.82,2.88) |
| Guyana | 1  (0,2) | 0.35  (0.06,0.69) | 3  (1,5) | 0.8  (0.19,1.49) | 3.01  (2.61,3.42) | 27  (5,52) | 11.5  (2.06,22.39) | 99  (24,182) | 25.72  (6.16,47.45) | 2.95  (2.55,3.36) |
| Haiti | 1  (0,4) | 0.07  (-0.01,0.22) | 11  (1,25) | 0.23  (0.03,0.54) | 4.16  (4.05,4.27) | 53  (0,159) | 2.53  (-0.09,7.72) | 409  (49,912) | 7.86  (0.92,17.77) | 4.02  (3.92,4.13) |
| Honduras | 3  (1,6) | 0.28  (0.05,0.56) | 25  (6,51) | 0.68  (0.17,1.38) | 2.85  (2.65,3.05) | 105  (18,207) | 8.73  (1.56,17.55) | 825  (209,1716) | 21.07  (5.34,43.32) | 2.79  (2.59,3) |
| Hungary | 73  (18,130) | 0.88  (0.21,1.55) | 98  (26,178) | 0.92  (0.25,1.66) | 0.35  (0.12,0.59) | 2001  (475,3549) | 25.54  (5.92,45.27) | 2421  (653,4359) | 25.94  (7.06,46.62) | 0.21  (-0.02,0.44) |
| Iceland | 1  (0,2) | 0.82  (0.17,1.57) | 2  (0,4) | 0.65  (0.15,1.23) | -0.77  (-0.98,-0.55) | 31  (6,59) | 22.45  (4.78,42.45) | 45  (11,84) | 16.69  (3.98,31.22) | -1.07  (-1.27,-0.87) |
| India | 66  (-11,153) | 0.03  (-0.01,0.06) | 910  (153,1740) | 0.14  (0.02,0.27) | 5.75  (5.64,5.87) | 2358  (-354,5462) | 0.85  (-0.14,1.98) | 28797  (4800,55470) | 4.34  (0.73,8.35) | 5.39  (5.3,5.49) |
| Indonesia | 16  (-7,47) | 0.02  (-0.01,0.07) | 240  (35,510) | 0.16  (0.02,0.35) | 6.56  (6.11,7.02) | 652  (-216,1878) | 0.95  (-0.36,2.74) | 9068  (1450,19332) | 5.86  (0.88,12.53) | 6.23  (5.77,6.69) |
| Iran (Islamic Republic of) | 15  (3,31) | 0.11  (0.02,0.23) | 143  (43,254) | 0.35  (0.1,0.62) | 4.05  (3.9,4.2) | 532  (100,1071) | 3.47  (0.67,6.99) | 4519  (1371,8038) | 10.21  (3.08,18.15) | 3.82  (3.67,3.96) |
| Iraq | 11  (2,25) | 0.27  (0.06,0.6) | 62  (16,120) | 0.47  (0.12,0.89) | 1.91  (1.8,2.03) | 387  (81,841) | 8.46  (1.8,18.47) | 2112  (546,4148) | 14.22  (3.65,27.48) | 1.81  (1.71,1.91) |
| Ireland | 17  (4,32) | 0.8  (0.17,1.53) | 29  (7,55) | 0.72  (0.17,1.32) | -0.23  (-0.4,-0.05) | 448  (94,854) | 22.84  (4.79,43.51) | 702  (166,1285) | 18.49  (4.33,33.7) | -0.58  (-0.75,-0.42) |
| Israel | 18  (4,35) | 0.72  (0.15,1.36) | 38  (9,69) | 0.58  (0.14,1.04) | -0.77  (-0.93,-0.6) | 481  (105,911) | 19.33  (4.21,36.73) | 883  (207,1589) | 14.5  (3.38,26.09) | -0.93  (-1.09,-0.76) |
| Italy | 192  (39,367) | 0.39  (0.08,0.74) | 384  (83,719) | 0.49  (0.11,0.92) | 0.56  (0.42,0.7) | 4829  (935,9272) | 10.57  (1.98,20.37) | 8049  (1771,15259) | 12.26  (2.7,23.33) | 0.26  (0.14,0.39) |
| Jamaica | 4  (1,7) | 0.42  (0.09,0.77) | 13  (4,23) | 0.79  (0.22,1.43) | 2.1(  1.92,2.27) | 116  (25,210) | 13.34  (2.89,23.96) | 384  (109,689) | 24.48  (6.95,44) | 2.05  (1.86,2.25) |
| Japan | 80  (-6,175) | 0.09  (-0.01,0.19) | 180  (12,362) | 0.11  (0.01,0.23) | 0.64  (0.53,0.75) | 2406  (-182,5392) | 2.65  (-0.24,5.97) | 4152  (318,8616) | 3.38  (0.27,6.98) | 0.59  (0.49,0.68) |
| Jordan | 3  (1,5) | 0.36  (0.09,0.71) | 25  (7,46) | 0.67  (0.2,1.22) | 1.78  (1.43,2.14) | 84  (20,162) | 10.66  (2.65,20.65) | 787  (232,1443) | 18.53  (5.48,33.87) | 1.61  (1.28,1.93) |
| Kazakhstan | 37  (8,69) | 0.48  (0.11,0.88) | 76  (20,136) | 0.7  (0.18,1.25) | 0.94  (0.64,1.25) | 1148  (256,2099) | 14.71  (3.26,26.73) | 2355  (588,4213) | 21.26  (5.3,38.01) | 0.87  (0.55,1.18) |
| Kenya | 4  (0,8) | 0.08  (0.01,0.17) | 44  (10,89) | 0.31  (0.07,0.63) | 4.65  (4.57,4.72) | 136  (15,293) | 2.65  (0.27,5.73) | 1554  (362,3173) | 10.11  (2.32,20.55) | 4.53  (4.46,4.6) |
| Kiribati | 0  (0,0) | 0.05  (0.01,0.1) | 0  (0,0) | 0.12  (0.03,0.24) | 2.87  (2.7,3.03) | 0  (0,1) | 1.8  (0.4,3.52) | 2  (1,4) | 4.04  (1.11,8) | 2.69  (2.52,2.85) |
| Kuwait | 1  (0,2) | 0.52  (0.14,0.93) | 8(  3,14) | 0.55  (0.17,0.94) | 0.32  (-0.02,0.67) | 43  (11,76) | 15.04  (4.02,26.71) | 294  (90,501) | 15.58  (4.86,26.41) | 0.02  (-0.31,0.36) |
| Kyrgyzstan | 6  (1,11) | 0.33  (0.07,0.63) | 23  (6,42) | 0.79  (0.19,1.43) | 4.15  (3.47,4.83) | 171  (38,320) | 9.68  (2.18,18.3) | 741  (179,1341) | 23.92  (5.78,43.49) | 4.15  (3.52,4.78) |
| Lao People's Democratic Republic | 0  (0,1) | 0.03  (-0.01,0.1) | 5  (1,10) | 0.17  (0.02,0.37) | 6.24  (5.88,6.61) | 13  (-3,44) | 1.04  (-0.21,3.39) | 176  (19,381) | 5.82  (0.63,12.61) | 6.29  (5.9,6.68) |
| Latvia | 17  (4,31) | 0.76  (0.18,1.4) | 26  (6,48) | 1.18  (0.29,2.2) | 1.25  (0.86,1.64) | 476  (114,867) | 22.86  (5.43,41.65) | 641  (155,1191) | 33.93  (8.25,64.75) | 1.02  (0.65,1.39) |
| Lebanon | 5  (1,10) | 0.4  (0.08,0.89) | 23  (6,42) | 0.71  (0.17,1.27) | 2.05  (1.85,2.26) | 136  (28,297) | 11.26  (2.33,24.63) | 592  (148,1068) | 18.81  (4.7,33.92) | 1.81  (1.64,1.98) |
| Lesotho | 2  (0,4) | 0.31  (0.07,0.67) | 5  (1,11) | 0.79  (0.18,1.68) | 3.42  (3.2,3.64) | 50  (11,108) | 8.82  (1.93,19.09) | 160  (36,342) | 22.49  (5.13,47.89) | 3.42  (3.19,3.64) |
| Liberia | 1  (0,1) | 0.11  (0.02,0.21) | 3  (1,6) | 0.25  (0.06,0.5) | 3.01  (2.86,3.16) | 19  (3,38) | 3.32  (0.58,6.58) | 100  (23,198) | 7.65  (1.74,15.24) | 2.9  (2.75,3.05) |
| Libya | 4  (1,7) | 0.39  (0.08,0.8) | 30  (8,56) | 1.06  (0.28,1.96) | 3.4  (3.23,3.56) | 113  (23,236) | 11.66  (2.36,24.21) | 978  (264,1831) | 30.83  (8.32,57.05) | 3.28  (3.12,3.44) |
| Lithuania | 20  (5,37) | 0.72  (0.17,1.36) | 33  (8,61) | 0.99  (0.24,1.83) | 0.31  (-0.17,0.79) | 542  (125,1028) | 20.58  (4.73,39.44) | 782  (192,1450) | 27.08  (6.53,50.53) | 0.16  (-0.34,0.67) |
| Luxembourg | 3  (1,5) | 0.84  (0.17,1.57) | 4  (1,7) | 0.69  (0.17,1.28) | -0.29  (-0.46,-0.11) | 64  (13,120) | 22.07  (4.49,41.58) | 87  (22,162) | 16.8  (4.23,31.29) | -0.57  (-0.75,-0.39) |
| Madagascar | 2  (0,4) | 0.07  (0,0.15) | 12  (2,27) | 0.19  (0.03,0.42) | 3.48  (3.19,3.76) | 53  (-3,127) | 1.87  (-0.05,4.34) | 406  (61,893) | 5.53  (0.89,12.31) | 3.51  (3.22,3.8) |
| Malawi | 0  (0,1) | 0.02  (-0.02,0.06) | 6  (1,14) | 0.11  (0.01,0.28) | 6.47  (6.39,6.55) | 19  (-9,56) | 0.68  (-0.41,2.07) | 230  (28,513) | 4.2  (0.45,9.71) | 6  (5.89,6.1) |
| Malaysia | 7  (1,15) | 0.14  (0.02,0.29) | 57  (14,109) | 0.38  (0.09,0.72) | 3.58  (3.11,4.05) | 264  (47,542) | 4.69  (0.85,9.63) | 1856  (449,3636) | 11.96  (2.89,23.29) | 3.38  (2.92,3.85) |
| Maldives | 0  (0,0) | 0.1  (0.01,0.33) | 1  (0,1) | 0.31  (0.07,0.63) | 3.52  (3.25,3.78) | 2  (0,7) | 3.79  (0.32,12.49) | 21  (5,45) | 10.94  (2.48,22.79) | 3.46  (3.18,3.75) |
| Mali | 1  (0,2) | 0.03  (0,0.07) | 3  (0,6) | 0.06  (0.01,0.13) | 1.85  (1.72,1.97) | 24  (3,48) | 1.03  (0.12,2.08) | 102  (17,212) | 1.89  (0.32,3.89) | 1.95  (1.82,2.09) |
| Malta | 1  (0,2) | 0.56  (0.1,1.05) | 4  (1,7) | 0.75  (0.18,1.4) | 0.6  (0.29,0.92) | 35  (6,66) | 15.09  (2.7,28.56) | 85  (21,158) | 20.05  (4.95,37.32) | 0.62  (0.32,0.91) |
| Marshall Islands | 0  (0,0) | 0.22  (0.05,0.47) | 0  (0,0) | 0.5  (0.11,1.07) | 2.65  (2.53,2.76) | 1  (0,1) | 7.14  (1.81,14.85) | 4  (1,8) | 15.97  (3.58,35.65) | 2.61  (2.49,2.73) |
| Mauritania | 1  (0,1) | 0.13  (0.03,0.24) | 3  (1,6) | 0.27  (0.06,0.52) | 2.3  (2.22,2.38) | 21  (4,41) | 3.77  (0.78,7.29) | 100  (23,200) | 7.85  (1.81,15.57) | 2.24  (2.16,2.32) |
| Mauritius | 1  (0,2) | 0.18  (0.03,0.35) | 6  (1,10) | 0.57  (0.13,1.05) | 3.51  (3.11,3.91) | 27  (5,51) | 6.01  (1.04,11.71) | 177  (41,333) | 19.03  (4.4,35.59) | 3.55  (3.13,3.97) |
| Mexico | 93  (21,171) | 0.4  (0.09,0.73) | 544  (155,990) | 0.77  (0.22,1.41) | 2.13  (2.07,2.2) | 3090  (696,5637) | 11.95  (2.7,21.86) | 17708  (5016,31954) | 24.53  (6.95,44.33) | 2.29  (2.22,2.36) |
| Micronesia (Federated States of) | 0  (0,0) | 0.22  (0.05,0.44) | 0  (0,0) | 0.5  (0.11,1.05) | 2.66  (2.58,2.74) | 2  (1,4) | 7.57  (1.78,15.23) | 8  (2,16) | 16.53  (3.87,34.73) | 2.56  (2.47,2.64) |
| Monaco | 0  (0,0) | 0.61  (0.11,1.25) | 0  (0,1) | 0.67  (0.15,1.45) | 0.18  (0.13,0.24) | 5  (1,11) | 17.17  (3.18,35.59) | 7  (2,16) | 17.88  (4.02,38.56) | -0.04  (-0.1,0.02) |
| Mongolia | 2  (0,3) | 0.26  (0.04,0.55) | 6  (1,12) | 0.42  (0.08,0.83) | 1.45  (1.32,1.58) | 51  (8,107) | 8.48  (1.32,17.89) | 205  (42,414) | 12.83  (2.61,25.88) | 1.32  (1.18,1.46) |
| Montenegro | 2  (0,3) | 0.49  (0.11,0.96) | 4  (1,8) | 0.77  (0.2,1.42) | 1.57  (1.29,1.85) | 51  (12,99) | 14.46  (3.27,28.03) | 105  (27,193) | 20.64  (5.39,38.29) | 1.38  (1.03,1.72) |
| Morocco | 12  (2,25) | 0.17  (0.03,0.34) | 73  (18,140) | 0.4  (0.1,0.77) | 2.98  (2.91,3.04) | 396  (73,792) | 5.03  (0.93,10.3) | 2296  (575,4427) | 12.01  (2.99,23.03) | 2.97  (2.92,3.03) |
| Mozambique | 3  (0,6) | 0.07  (0,0.17) | 19  (3,41) | 0.26  (0.04,0.58) | 4.58  (4.36,4.79) | 86  (-3,201) | 2.26  (-0.07,5.3) | 673  (113,1456) | 8.52  (1.39,18.55) | 4.58  (4.36,4.79) |
| Myanmar | 9  (0,22) | 0.06  (0,0.16) | 49  (6,104) | 0.16  (0.02,0.34) | 2.8  (2.65,2.96) | 346  (8,832) | 2.38  (0.05,5.77) | 1764  (245,3689) | 5.7  (0.76,11.91) | 2.64  (2.47,2.81) |
| Namibia | 1  (0,1) | 0.15  (0.03,0.29) | 3  (1,6) | 0.38  (0.09,0.77) | 2.87  (2.68,3.06) | 17  (4,35) | 4.45  (0.9,8.76) | 99  (22,198) | 10.98  (2.44,22.06) | 2.7  (2.51,2.89) |
| Nauru | 0  (0,0) | 0.29  (0.07,0.66) | 0  (0,0) | 0.55  (0.12,1.19) | 2.14  (2.09,2.19) | 0  (0,1) | 10  (2.46,21.93) | 1  (0,2) | 18.25  (4.11,40.3) | 2.05  (1.99,2.11) |
| Nepal | 1  (-1,3) | 0.02  (-0.01,0.06) | 12  (1,28) | 0.08  (0,0.2) | 5.69  (5.47,5.91) | 38  (-15,116) | 0.64  (-0.29,1.96) | 436  (31,1031) | 3.02  (0.19,7.15) | 5.57  (5.33,5.82) |
| Netherlands | 75  (14,144) | 0.67  (0.13,1.28) | 112  (23,208) | 0.6  (0.13,1.11) | -0.58  (-0.68,-0.48) | 1832  (346,3446) | 17.96  (3.37,33.79) | 2443  (521,4491) | 14.9  (3.25,27.37) | -0.82  (-0.93,-0.71) |
| New Zealand | 15  (3,28) | 0.73  (0.16,1.34) | 22  (5,41) | 0.51  (0.12,0.92) | -1.13  (-1.3,-0.95) | 407  (92,743) | 20.75  (4.7,37.82) | 538  (128,959) | 13.57  (3.25,24.14) | -1.37  (-1.54,-1.2) |
| Nicaragua | 1  (0,3) | 0.16  (0.03,0.32) | 10  (3,20) | 0.37  (0.1,0.7) | 3.02  (2.69,3.35) | 47  (10,97) | 4.86  (1.1,10.02) | 333  (90,634) | 11.25  (3.07,21.36) | 2.95  (2.63,3.26) |
| Niger | 1  (0,1) | 0.04  (0.01,0.09) | 4  (1,8) | 0.08  (0.01,0.17) | 1.72  (1.66,1.78) | 23  (3,49) | 1.41  (0.17,2.99) | 122  (21,275) | 2.34  (0.4,5.35) | 1.6  (1.54,1.67) |
| Nigeria | 16  (2,32) | 0.07  (0.01,0.15) | 133  (27,260) | 0.26  (0.05,0.5) | 4.08  (4,4.15) | 447  (48,919) | 2.03  (0.23,4.17) | 4137  (829,8093) | 6.96  (1.42,13.67) | 4.02  (3.94,4.11) |
| Niue | 0  (0,0) | 0.22  (0.05,0.43) | 0  (0,0) | 0.5  (0.14,0.97) | 2.53  (2.45,2.61) | 0  (0,0) | 7.3  (1.69,14.31) | 0  (0,0) | 16.55  (4.73,31.87) | 2.41  (2.33,2.5) |
| North Macedonia | 6  (1,11) | 0.58  (0.13,1.14) | 16  (4,30) | 0.93  (0.23,1.74) | 1.5  (1.26,1.73) | 176  (40,340) | 16.79  (3.82,32.74) | 430  (110,819) | 25.07  (6.38,47.79) | 1.28  (1.05,1.5) |
| Northern Mariana Islands | 0  (0,0) | 0.24  (0.05,0.5) | 0  (0,0) | 0.62  (0.16,1.07) | 3.77  (3.37,4.18) | 1  (0,2) | 7.79  (1.8,16.89) | 6  (2,10) | 19.71  (5.46,33.67) | 3.66  (3.27,4.05) |
| Norway | 21  (4,40) | 0.62  (0.11,1.17) | 29  (6,54) | 0.55  (0.12,1.04) | -0.52  (-0.72,-0.32) | 522  (97,985) | 17.3  (3.31,32.75) | 634  (139,1193) | 13.71  (3.03,25.57) | -0.85  (-1.09,-0.6) |
| Oman | 0  (0,1) | 0.11  (0.02,0.24) | 3  (1,5) | 0.29  (0.08,0.51) | 3.19  (2.87,3.51) | 12  (2,26) | 3.48  (0.57,7.61) | 93  (27,164) | 8.44  (2.43,15.01) | 2.99  (2.65,3.32) |
| Pakistan | 24  (-2,57) | 0.08  (-0.01,0.2) | 301  (59,618) | 0.45  (0.09,0.94) | 6.09  (5.68,6.5) | 850  (-31,1934) | 2.8  (-0.17,6.49) | 10502  (2031,21439) | 13.97  (2.75,28.75) | 5.88  (5.49,6.26) |
| Palau | 0  (0,0) | 0.07  (0.01,0.13) | 0  (0,0) | 0.11  (0.03,0.2) | 1.74  (1.67,1.81) | 0  (0,0) | 2.02  (0.46,3.86) | 0  (0,1) | 3.24  (0.9,6.05) | 1.51  (1.44,1.58) |
| Palestine | 2  (0,3) | 0.32  (0.06,0.69) | 9  (2,16) | 0.64  (0.18,1.18) | 2.52  (2.43,2.61) | 48  (10,107) | 9.25  (1.84,20.48) | 281  (78,518) | 18.12  (5.05,33.49) | 2.36  (2.28,2.44) |
| Panama | 1  (0,2) | 0.18  (0.04,0.33) | 12  (4,21) | 0.53  (0.16,0.93) | 3.69  (3.21,4.17) | 36  (7,68) | 4.68  (1.01,8.71) | 344  (99,608) | 15.35  (4.4,27.19) | 4  (3.55,4.45) |
| Papua New Guinea | 1  (0,1) | 0.05  (0,0.12) | 3  (1,7) | 0.1  (0.02,0.23) | 2.39  (2.25,2.54) | 19  (2,47) | 1.69  (0.18,4.16) | 128  (24,284) | 3.65  (0.65,8.05) | 2.43  (2.28,2.57) |
| Paraguay | 2  (0,4) | 0.16  (0.03,0.35) | 12  (3,23) | 0.38  (0.08,0.75) | 2.79  (2.53,3.05) | 61  (12,129) | 4.91  (0.94,10.42) | 360  (82,701) | 11.25  (2.55,21.94) | 2.65  (2.38,2.92) |
| Peru | 14  (2,29) | 0.22  (0.04,0.45) | 84  (20,167) | 0.47  (0.11,0.93) | 2.39  (2.24,2.55) | 483  (87,995) | 7  (1.25,14.29) | 2687  (630,5367) | 14.78  (3.48,29.47) | 2.34  (2.19,2.49) |
| Philippines | 17  (1,35) | 0.1  (0,0.2) | 144  (24,273) | 0.3  (0.05,0.56) | 3.74  (3.58,3.91) | 650  (45,1361) | 3.32  (0.23,6.87) | 5133  (895,9931) | 10.13  (1.75,19.5) | 3.65  (3.5,3.81) |
| Poland | 214  (48,389) | 0.85  (0.19,1.55) | 438  (113,801) | 1.08  (0.28,1.98) | 0.72  (0.51,0.93) | 5966  (1278,10853) | 24.84  (5.31,45.09) | 10306  (2628,18872) | 28.45  (7.06,52.51) | 0.39  (0.16,0.61) |
| Portugal | 25  (5,48) | 0.33  (0.06,0.63) | 51  (12,94) | 0.39  (0.09,0.71) | 0.35  (0.1,0.6) | 665  (121,1295) | 9.25  (1.66,18.1) | 1136  (267,2064) | 10.34  (2.43,18.64) | 0.14  (-0.11,0.4) |
| Puerto Rico | 5  (1,9) | 0.27  (0.06,0.48) | 21  (6,37) | 0.59  (0.16,1.05) | 2.5  (1.87,3.12) | 145  (35,255) | 7.58  (1.85,13.29) | 517  (146,921) | 17.59  (5.08,31.02) | 2.68  (2.03,3.34) |
| Qatar | 0  (0,1) | 0.67  (0.15,1.36) | 4  (1,8) | 1.23  (0.38,2.27) | 2.39  (2.14,2.65) | 10  (2,21) | 18.63  (4.15,37.22) | 144  (44,282) | 31.93  (9.75,58.89) | 2.15  (1.94,2.36) |
| Republic of Korea | 3  (-3,12) | 0.02  (-0.01,0.06) | 61  (10,119) | 0.12  (0.02,0.23) | 6.22  (5.93,6.51) | 108  (-102,397) | 0.56  (-0.49,2.06) | 1596  (228,3062) | 3.37  (0.42,6.47) | 6  (5.8,6.2) |
| Republic of Moldova | 18  (4,32) | 0.66  (0.16,1.2) | 21  (5,37) | 0.63  (0.16,1.09) | 0.13  (-0.22,0.48) | 541  (130,969) | 20.27  (4.84,36.59) | 606  (157,1050) | 18.87  (4.87,32.61) | 0.01  (-0.33,0.36) |
| Romania | 73  (16,133) | 0.48  (0.1,0.87) | 158  (35,290) | 0.8  (0.18,1.48) | 1.62  (1.47,1.77) | 2271  (502,4192) | 15.18  (3.32,28.39) | 4011  (910,7381) | 22.96  (5.19,42.09) | 1.26  (1.11,1.41) |
| Russian Federation | 844  (203,1525) | 0.75  (0.18,1.35) | 1330  (362,2327) | 0.94  (0.25,1.64) | 0.69  (0.51,0.86) | 25311  (6054,45685) | 23.5  (5.58,42.28) | 36074  (9766,62744) | 27.59  (7.42,47.69) | 0.41  (0.25,0.57) |
| Rwanda | 1  (0,3) | 0.06  (-0.02,0.18) | 10  (1,22) | 0.23  (0.03,0.53) | 4.15  (3.97,4.33) | 38  (-10,109) | 1.99  (-0.56,5.7) | 323  (41,720) | 7.19  (0.88,16.41) | 3.98  (3.76,4.2) |
| Saint Kitts and Nevis | 0  (0,0) | 0.51  (0.1,1.07) | 0  (0,1) | 0.8  (0.21,1.45) | 1.78  (1.61,1.94) | 3  (1,6) | 15.96  (3.21,34.22) | 9  (3,16) | 22.27  (6.25,40.27) | 1.27  (1.14,1.41) |
| Saint Lucia | 0  (0,0) | 0.43  (0.08,0.86) | 1  (0,2) | 0.75  (0.2,1.41) | 1.56  (1.42,1.7) | 7  (2,14) | 14.94  (3.34,29.57) | 30  (8,55) | 24.42  (6.82,44.54) | 1.52  (1.4,1.63) |
| Saint Vincent and the Grenadines | 0  (0,0) | 0.28  (0.04,0.57) | 0  (0,1) | 0.59  (0.14,1.12) | 2.45  (2.28,2.62) | 3  (1,7) | 9.04  (1.44,18.37) | 13  (3,25) | 19.45  (4.68,36.54) | 2.43  (2.27,2.6) |
| Samoa | 0  (0,0) | 0.37  (0.1,0.69) | 0  (0,1) | 0.64  (0.16,1.2) | 1.73  (1.67,1.79) | 6  (2,11) | 12.06  (3.55,22.19) | 16  (4,32) | 20.35  (5.41,39.33) | 1.69  (1.63,1.75) |
| San Marino | 0  (0,0) | 0.38  (0.08,0.76) | 0  (0,0) | 0.27  (0.06,0.57) | -0.36  (-0.61,-0.1) | 2  (0,3) | 9.74  (2.08,19.11) | 2  (0,5) | 7.23  (1.46,15.14) | -0.18  (-0.45,0.1) |
| Sao Tome and Principe | 0  (0,0) | 0.21  (0.04,0.4) | 0  (0,1) | 0.49  (0.12,1.04) | 2.69  (2.62,2.76) | 2  (0,4) | 6.44  (1.23,12.69) | 10  (3,21) | 14.88  (3.61,31.55) | 2.63  (2.55,2.71) |
| Saudi Arabia | 6  (1,14) | 0.24  (0.05,0.51) | 59  (17,117) | 0.61  (0.19,1.21) | 3  (2.74,3.26) | 212  (46,459) | 7.17  (1.55,15.46) | 2158  (626,4417) | 17.92  (5.37,36.38) | 2.93  (2.68,3.18) |
| Senegal | 1  (0,3) | 0.08  (0.01,0.15) | 8  (2,17) | 0.18  (0.04,0.37) | 2.97  (2.83,3.11) | 47  (8,94) | 2.48  (0.42,4.92) | 271  (55,556) | 5.65  (1.14,11.54) | 2.8  (2.66,2.94) |
| Serbia | 38  (8,73) | 0.61  (0.13,1.18) | 93  (28,165) | 1.08  (0.33,1.92) | 1.8  (1.69,1.91) | 1128  (251,2162) | 17.63  (3.84,34.01) | 2360  (720,4176) | 30.12  (8.99,53.93) | 1.7  (1.56,1.84) |
| Seychelles | 0  (0,0) | 0.46  (0.1,0.89) | 1  (0,1) | 1.03  (0.28,1.82) | 2.87  (2.61,3.13) | 5  (1,9) | 16.17  (3.51,30.83) | 21  (6,38) | 34.3  (9.5,60.74) | 2.71  (2.48,2.94) |
| Sierra Leone | 1  (0,1) | 0.05  (0.01,0.11) | 3  (0,5) | 0.13  (0.02,0.27) | 3.11  (2.99,3.23) | 16  (2,35) | 1.56  (0.19,3.29) | 86  (15,180) | 3.91  (0.7,8.25) | 3.11  (2.97,3.25) |
| Singapore | 1  (0,2) | 0.06  (-0.03,0.17) | 11  (2,20) | 0.25  (0.06,0.46) | 4.14  (3.58,4.7) | 31  (-10,81) | 2.21  (-0.69,5.76) | 324  (75,585) | 7.64  (1.76,13.89) | 3.77  (3.19,4.35) |
| Slovakia | 31  (8,57) | 0.9  (0.22,1.71) | 51  (14,93) | 0.97  (0.27,1.76) | 0.18  (0.08,0.28) | 848  (204,1603) | 25.98  (6.12,49.55) | 1301  (367,2348) | 26.57  (7.38,47.68) | 0.03  (-0.07,0.13) |
| Slovenia | 11  (3,19) | 0.74  (0.17,1.32) | 17  (4,31) | 0.71  (0.18,1.3) | 0.04  (-0.18,0.25) | 288  (67,511) | 20.77  (4.81,36.73) | 374  (93,690) | 18.27  (4.56,33.97) | -0.22  (-0.46,0.02) |
| Solomon Islands | 0  (0,0) | 0.08  (0.01,0.19) | 0  (0,1) | 0.21  (0.04,0.47) | 3.39  (3.28,3.51) | 2  (0,6) | 2.77  (0.43,6.83) | 18  (4,41) | 7.51  (1.57,16.99) | 3.42  (3.31,3.53) |
| Somalia | 1  (0,3) | 0.0  7(0,0.18) | 7  (1,16) | 0.16  (0.01,0.35) | 2.68  (2.6,2.76) | 52  (4,127) | 2.63  (0.1,6.51) | 288  (38,629) | 5.7  (0.62,12.23) | 2.55  (2.47,2.63) |
| South Africa | 48  (12,89) | 0.39  (0.1,0.74) | 227  (63,401) | 0.83  (0.23,1.47) | 2.62  (2.51,2.74) | 1502  (387,2828) | 11.67  (3.04,21.9) | 6725  (1879,11838) | 23.6  (6.6,41.6) | 2.5  (2.37,2.63) |
| South Sudan | 0  (0,1) | 0.03  (-0.02,0.09) | 1  (0,4) | 0.06  (-0.02,0.17) | 2.33  (2.13,2.54) | 12  (-6,34) | 1  (-0.5,2.91) | 53  (-10,139) | 2.03  (-0.44,5.47) | 2.24  (2.02,2.46) |
| Spain | 143  (29,264) | 0.48  (0.1,0.89) | 284  (74,512) | 0.56  (0.14,1) | 0.21  (0.03,0.39) | 3735  (762,6927) | 13.65  (2.75,25.14) | 6519  (1691,11788) | 14.77  (3.8,26.79) | 0  (-0.17,0.16) |
| Sri Lanka | 4  (0,8) | 0.07  (0.01,0.14) | 29  (6,60) | 0.19  (0.04,0.39) | 3.81  (3.56,4.06) | 139  (12,300) | 2.14  (0.18,4.63) | 870  (187,1827) | 5.88  (1.25,12.32) | 3.57  (3.33,3.81) |
| Sudan | 5  (1,15) | 0.12  (0.02,0.33) | 28  (7,60) | 0.27  (0.07,0.58) | 2.83  (2.63,3.03) | 183  (27,512) | 3.54  (0.52,10.02) | 1014  (243,2140) | 8.27  (2.04,17.55) | 2.83  (2.63,3.03) |
| Suriname | 0  (0,1) | 0.24  (0.03,0.51) | 2  (0,3) | 0.49  (0.11,0.96) | 2.65  (2.47,2.84) | 11  (2,23) | 7.59  (1.08,15.75) | 55  (12,104) | 15.79  (3.57,30.18) | 2.56  (2.38,2.73) |
| Sweden | 54  (10,103) | 0.71  (0.14,1.36) | 59  (14,113) | 0.53  (0.12,1) | -1.06  (-1.31,-0.81) | 1310  (262,2516) | 19.65  (4.02,37.81) | 1232  (282,2337) | 13.03  (2.99,24.57) | -1.38  (-1.63,-1.12) |
| Switzerland | 19  (4,34) | 0.32  (0.06,0.59) | 37  (7,70) | 0.37  (0.08,0.69) | 0.15  (-0.28,0.59) | 441  (82,813) | 8.48  (1.53,15.87) | 745  (154,1394) | 8.54  (1.77,15.89) | -0.29  (-0.73,0.15) |
| Syrian Arab Republic | 4  (1,9) | 0.16  (0.03,0.34) | 26  (7,48) | 0.38  (0.11,0.69) | 2.57  (2.45,2.7) | 148  (30,301) | 4.97  (1.01,10.05) | 821  (236,1502) | 10.9  (3.14,19.84) | 2.41  (2.27,2.54) |
| Taiwan (Province of China) | 5  (0,11) | 0.07  (0,0.14) | 56  (11,105) | 0.26  (0.05,0.49) | 4.19  (3.84,4.54) | 153  (-7,332) | 1.89  (-0.05,4.03) | 1701  (331,3193) | 8.51  (1.65,15.85) | 4.62  (4.3,4.95) |
| Tajikistan | 2  (1,5) | 0.15  (0.03,0.31) | 7  (2,14) | 0.2  (0.05,0.4) | 0.63  (0.46,0.81) | 71  (15,144) | 4.58  (0.97,9.23) | 236  (54,484) | 6.2  (1.41,12.51) | 0.82  (0.7,0.95) |
| Thailand | 17  (1,35) | 0.08  (0,0.17) | 194  (44,380) | 0.34  (0.08,0.67) | 4.83  (4.59,5.07) | 624  (25,1297) | 2.67  (0.11,5.6) | 6269  (1492,12360) | 11.52  (2.7,22.74) | 4.72  (4.46,4.97) |
| Timor-Leste | 0  (0,0) | 0  (-0.02,0.01) | 0  (0,0) | 0.03  (-0.01,0.1) | 22.88  (19.02,26.86) | 0  (-1,1) | -0.04  (-0.49,0.54) | 7  (-1,18) | 1.39  (-0.32,3.75) | 18.09  (16.05,20.16) |
| Togo | 1  (0,1) | 0.07  (0.01,0.14) | 4  (1,9) | 0.17  (0.03,0.37) | 2.99  (2.91,3.07) | 17  (3,36) | 2.14  (0.37,4.52) | 144  (29,298) | 5.33  (1.07,10.96) | 2.91  (2.82,3) |
| Tokelau | 0  (0,0) | 0.18  (0.04,0.39) | 0  (0,0) | 0.42  (0.12,0.83) | 2.62  (2.52,2.72) | 0  (0,0) | 6.29  (1.36,13.14) | 0  (0,0) | 14.17  (4.22,27.87) | 2.53  (2.44,2.63) |
| Tonga | 0  (0,0) | 0.22  (0.05,0.39) | 0  (0,0) | 0.42  (0.12,0.78) | 2.13  (2.01,2.25) | 2  (1,4) | 7.18  (1.78,12.58) | 6  (2,11) | 13.16  (3.94,24.36) | 1.97  (1.85,2.09) |
| Trinidad and Tobago | 3  (1,5) | 0.64  (0.15,1.22) | 9  (2,16) | 0.92  (0.25,1.66) | 0.99  (0.83,1.16) | 94  (22,175) | 20.53  (4.79,38) | 278  (77,505) | 29.37  (8.2,54.33) | 1  (0.85,1.16) |
| Tunisia | 5  (1,10) | 0.19  (0.04,0.37) | 32  (9,62) | 0.45  (0.12,0.88) | 2.79  (2.75,2.83) | 156  (34,298) | 5.69  (1.23,10.86) | 953  (254,1813) | 13.27  (3.54,25.3) | 2.71  (2.68,2.74) |
| Turkey | 103  (22,215) | 0.54  (0.12,1.12) | 391  (110,704) | 0.77  (0.22,1.4) | 1.08  (0.91,1.26) | 3283  (696,6955) | 16.17  (3.46,33.96) | 11177  (3096,20120) | 22.07  (6.07,39.86) | 0.87  (0.72,1.02) |
| Turkmenistan | 2  (0,4) | 0.19  (0.04,0.36) | 10  (2,20) | 0.42  (0.09,0.82) | 4.13  (1.98,6.33) | 70  (14,131) | 5.97  (1.2,11.27) | 340  (71,661) | 13.47  (2.79,26.25) | 4.26  (2.12,6.45) |
| Tuvalu | 0  (0,0) | 0.14  (0.03,0.3) | 0  (0,0) | 0.38  (0.1,0.74) | 3.33  (3.21,3.45) | 0  (0,0) | 4.77  (0.95,10.1) | 1  (0,1) | 12.34  (3.24,24.38) | 3.17  (3.05,3.28) |
| Uganda | 3  (0,7) | 0.08  (-0.01,0.21) | 30  (4,62) | 0.31  (0.04,0.65) | 3.89  (3.69,4.09) | 104  (-7,248) | 2.71  (-0.19,6.4) | 1048  (149,2257) | 9.97  (1.34,21.02) | 3.86  (3.65,4.06) |
| Ukraine | 233  (55,414) | 0.54  (0.12,0.95) | 337  (80,658) | 0.77  (0.18,1.52) | 1.19  (0.9,1.48) | 6928  (1596,12468) | 16.91  (3.82,30.41) | 9688  (2264,19110) | 24.1  (5.51,48.03) | 1.14  (0.82,1.45) |
| United Arab Emirates | 1  (0,4) | 0.83  (0.14,2) | 25  (8,45) | 3.73  (1.05,6.55) | 6.22  (5.72,6.74) | 53  (9,132) | 24.32  (4.07,60.28) | 908  (265,1651) | 80.08  (22.77,140.04) | 4.8  (4.44,5.16) |
| United Kingdom | 454  (97,861) | 0.95  (0.21,1.81) | 553  (134,1025) | 0.8  (0.2,1.47) | -0.66  (-0.84,-0.49) | 11374  (2501,21761) | 26.74  (5.93,51.27) | 12054  (2941,22042) | 20.04  (4.89,36.73) | -1.08  (-1.25,-0.9) |
| United Republic of Tanzania | 11  (2,21) | 0.18  (0.02,0.35) | 76  (17,147) | 0.52  (0.11,1) | 3.73  (3.64,3.82) | 355  (51,694) | 5.51  (0.81,10.77) | 2523  (564,4993) | 15.58  (3.46,30.62) | 3.6  (3.51,3.69) |
| United States of America | 1495  (355,2734) | 0.86  (0.21,1.55) | 2464  (666,4274) | 0.79  (0.22,1.37) | -0.38  (-0.62,-0.13) | 38469  (9395,69645) | 24  (5.91,43.36) | 58749  (16253,101446) | 20.97  (5.83,36.19) | -0.56  (-0.78,-0.34) |
| United States Virgin Islands | 0  (0,1) | 0.71  (0.17,1.35) | 1  (0,1) | 0.81  (0.23,1.56) | 0.88  (0.53,1.24) | 11  (3,21) | 21.12  (5.28,39.81) | 19  (5,36) | 25.8  (7.26,49.39) | 1.18  (0.84,1.52) |
| Uruguay | 12  (3,24) | 0.58  (0.13,1.1) | 22  (6,40) | 0.74  (0.19,1.34) | 0.77  (0.67,0.88) | 324  (74,619) | 16.2  (3.66,30.99) | 547(137,984) | 20.97  (5.2,37.6) | 0.79  (0.69,0.89) |
| Uzbekistan | 11  (2,22) | 0.17  (0.04,0.33) | 56  (13,109) | 0.35  (0.08,0.68) | 2.08  (1.81,2.34) | 351  (73,696) | 5.31  (1.12,10.49) | 1867  (424,3657) | 11.07  (2.53,21.64) | 2.16  (1.88,2.44) |
| Vanuatu | 0  (0,0) | 0.06  (0.01,0.15) | 0  (0,0) | 0.16  (0.03,0.37) | 2.98  (2.87,3.09) | 1  (0,2) | 1.98  (0.28,5.11) | 6  (1,14) | 5.03  (0.98,12.1) | 2.9  (2.77,3.03) |
| Venezuela (Bolivarian Republic of) | 7  (2,13) | 0.13  (0.03,0.24) | 102  (28,187) | 0.62  (0.17,1.14) | 4.54  (3.37,5.72) | 229  (51,430) | 3.82  (0.86,7.11) | 3132  (890,5801) | 19.1  (5.41,35.38) | 4.62  (3.43,5.83) |
| Viet Nam | 0  (-3,3) | 0  (-0.01,0.01) | 27  (0,63) | 0.05  (0,0.11) | 15.46  (14.53,16.39) | 6  (-73,94) | 0.03  (-0.3,0.4) | 882  (21,2071) | 1.45  (0.02,3.43) | 14.69  (13.89,15.5) |
| Yemen | 1  (0,3) | 0.04  (0,0.13) | 10  (2,23) | 0.13  (0.02,0.29) | 3.8  (3.51,4.09) | 40  (4,114) | 1.38  (0.13,3.94) | 356  (64,791) | 3.88  (0.72,8.52) | 3.61  (3.34,3.88) |
| Zambia | 1  (0,3) | 0.09  (-0.01,0.22) | 18  (4,36) | 0.44  (0.1,0.89) | 5.04  (4.94,5.14) | 49  (-3,117) | 2.81  (-0.14,6.72) | 627  (131,1267) | 13.44  (2.93,26.94) | 4.98  (4.88,5.08) |
| Zimbabwe | 5  (1,9) | 0.2  (0.04,0.42) | 34  (8,67) | 0.77  (0.18,1.49) | 4.8  (4.25,5.35) | 152  (29,311) | 6.37  (1.25,13.11) | 1146  (270,2240) | 23.47  (5.52,46.01) | 4.71  (4.12,5.31) |

BMI, body mass index; DALYs, disability-adjusted life years; ASMR, age-standardized mortality rate; ASDR, age-standardized DALY rate; EAPC, estimated annual percentage change; UI, uncertainty interval;

CI, confidence interval.

**Table S7** Deaths and DALYs for uterine cancer attributable to high BMI in 1990 and 2021, with corresponding EAPC from 1990 to 2021, in 204 countries

|  | **Deaths** | | | | | **DALYs** | | | | |
| --- | --- | --- | --- | --- | --- | --- | --- | --- | --- | --- |
| **Countries** | **Number of cases (95% UI), 1990** | **ASMR per 100,000 (95% UI), 1990** | **Number of cases (95% UI), 2021** | **ASMR per 100,000 (95% UI), 2021** | **EAPC of ASMR (95% CI),1990-2021** | **Number of**  **cases (95% UI), 1990** | **ASDR per 100,000 (95% UI), 1990** | **Number of**  **cases (95% UI),**  **2021** | **ASDR per 100,000 (95% UI), 2021** | **EAPC of ASDR (95% CI),**  **1990-2021** |
| Afghanistan | 19  (8,41) | 0.53  (0.22,1.13) | 43  (19,81) | 0.75  (0.35,1.4) | 1.25  (1.09,1.4) | 599  (233,1329) | 15.89  (6.36,35.05) | 1523  (666,2873) | 22.65  (10.15,42.9) | 1.26  (1.1,1.42) |
| Albania | 8  (5,13) | 0.81  (0.48,1.25) | 19  (10,29) | 0.8  (0.44,1.23) | 0.22  (0.01,0.43) | 218  (133,330) | 20.06  (12.17,30.66) | 439  (244,675) | 19.6  (11.13,30) | 0.21  (0.01,0.41) |
| Algeria | 8  (5,13) | 0.15  (0.09,0.24) | 35  (21,53) | 0.22  (0.13,0.33) | 1.61  (1.44,1.78) | 247  (150,381) | 3.82  (2.31,5.92) | 1010  (626,1567) | 5.47  (3.34,8.41) | 1.35  (1.24,1.46) |
| American Samoa | 0  (0,0) | 1.58  (0.87,2.33) | 1  (0,1) | 2.55  (1.09,4.94) | 1.68  (1.61,1.74) | 6  (3,9) | 46.92  (25.34,68.99) | 20  (9,38) | 76.56  (33.01,144.79) | 1.71  (1.64,1.78) |
| Andorra | 0  (0,0) | 0.55  (0.29,0.93) | 0  (0,1) | 0.48  (0.28,0.79) | -0.01  (-0.17,0.15) | 4  (2,7) | 13.97  (7.59,24.06) | 9  (5,16) | 12.59  (6.94,20.85) | 0.04  (-0.12,0.2) |
| Angola | 5  (3,8) | 0.24  (0.14,0.37) | 30  (16,53) | 0.44  (0.24,0.78) | 1.87  (1.71,2.02) | 158  (93,252) | 6.86  (4.09,10.94) | 959  (498,1659) | 12.47  (6.55,21.74) | 1.8  (1.64,1.95) |
| Antigua and Barbuda | 0  (0,0) | 0.88  (0.59,1.19) | 1  (1,2) | 2.1  (1.51,2.75) | 2.64  (2.29,2.99) | 7  (5,9) | 24.93  (16.99,34.05) | 32  (23,42) | 55.15  (40.02,71.93) | 2.43  (2.11,2.76) |
| Argentina | 204  (143,281) | 1.12  (0.79,1.54) | 297  (205,396) | 0.93  (0.65,1.24) | -0.57  (-0.7,-0.44) | 5298  (3711,7157) | 29.59  (20.74,39.91) | 7140  (5005,9368) | 23.85  (16.78,31.27) | -0.66  (-0.8,-0.51) |
| Armenia | 25  (17,32) | 1.56  (1.09,2.04) | 45  (31,62) | 1.78  (1.2,2.43) | 0.52  (-0.02,1.06) | 733  (515,959) | 45.03  (31.55,59) | 1168  (800,1585) | 47.1  (32.19,63.98) | 0.28  (-0.26,0.82) |
| Australia | 62  (44,87) | 0.57  (0.4,0.79) | 203  (140,270) | 0.81  (0.57,1.08) | 1.16  (0.9,1.43) | 1522  (1057,2114) | 14.64  (10.19,20.23) | 4688  (3310,6207) | 21.08  (14.97,27.87) | 1.19  (0.94,1.44) |
| Austria | 79  (55,108) | 1.03  (0.72,1.39) | 91  (62,127) | 0.83  (0.57,1.14) | -0.64  (-0.8,-0.48) | 1739  (1219,2355) | 25.21  (17.74,34.08) | 1921  (1326,2678) | 20.53  (14.17,28.45) | -0.52  (-0.67,-0.36) |
| Azerbaijan | 30  (19,42) | 1.03  (0.65,1.43) | 56  (35,84) | 0.94  (0.59,1.42) | -0.37  (-0.47,-0.27) | 940  (601,1313) | 30.96  (19.81,43.12) | 1738  (1087,2556) | 27.28  (17.06,40.36) | -0.58  (-0.69,-0.47) |
| Bahamas | 1  (1,2) | 1.37  (0.95,1.83) | 6  (4,8) | 2.51  (1.71,3.46) | 2.17  (1.82,2.52) | 39  (27,52) | 41.29  (28.64,54.9) | 167  (113,230) | 71.02  (47.87,97.18) | 1.92  (1.59,2.24) |
| Bahrain | 1  (0,1) | 0.86  (0.52,1.37) | 4  (3,6) | 1.2  (0.76,1.75) | 0.56  (0.3,0.82) | 21  (13,33) | 23.24  (14.19,36.9) | 137  (83,211) | 31.75  (19.69,47.53) | 0.5  (0.26,0.73) |
| Bangladesh | 14  (8,25) | 0.06  (0.04,0.12) | 86  (42,213) | 0.12  (0.06,0.31) | 2.43  (2.3,2.56) | 417  (240,749) | 1.77  (1.02,3.27) | 2638  (1276,6626) | 3.6  (1.75,9.03) | 2.65  (2.48,2.82) |
| Barbados | 3  (2,4) | 1.66  (1.13,2.24) | 8  (6,12) | 2.94  (1.95,4.15) | 2.31  (1.79,2.83) | 68  (47,93) | 46.72  (32.34,63.2) | 208  (139,292) | 77.86  (51.79,109.88) | 2.09  (1.6,2.59) |
| Belarus | 86  (60,116) | 1.03  (0.72,1.39) | 181  (122,255) | 1.76  (1.19,2.48) | 0.34  (-0.38,1.06) | 2355  (1637,3186) | 29.1  (20.35,39.35) | 4834  (3237,6731) | 50.02  (33.33,69.72) | 0.34  (-0.36,1.05) |
| Belgium | 68  (46,90) | 0.74  (0.51,0.99) | 126  (84,170) | 0.91  (0.63,1.22) | 0.56  (0.42,0.7) | 1579  (1089,2107) | 19.04  (13.16,25.45) | 2641  (1833,3551) | 22.62  (15.81,30.23) | 0.52  (0.38,0.65) |
| Belize | 1  (0,1) | 1.36  (0.93,1.87) | 3  (2,4) | 2.19  (1.55,2.83) | 1.31  (0.91,1.71) | 18  (13,25) | 37.93  (26.47,52.29) | 104  (74,134) | 62.01  (44.51,79.96) | 1.34  (0.96,1.73) |
| Benin | 5  (3,8) | 0.51  (0.32,0.8) | 18  (10,27) | 0.66  (0.39,1) | 0.92  (0.82,1.02) | 150  (93,237) | 14  (8.64,22.07) | 495  (292,786) | 17.1  (10.08,26.67) | 0.7  (0.61,0.78) |
| Bermuda | 1  (0,1) | 1.48  (1.01,2.02) | 1  (1,2) | 1.55  (1.08,2.21) | -0.4  (-0.73,-0.06) | 14  (9,19) | 38.67  (26.37,52.56) | 28  (19,39) | 41  (28.42,57.93) | -0.41  (-0.75,-0.07) |
| Bhutan | 0  (0,1) | 0.23  (0.12,0.44) | 1  (0,2) | 0.28  (0.14,0.67) | 0.43  (0.32,0.55) | 9  (5,17) | 6.74  (3.33,12.66) | 24  (11,58) | 7.68  (3.71,18.68) | 0.28  (0.16,0.39) |
| Bolivia (Plurinational State of) | 22  (12,37) | 1.24  (0.68,2.12) | 79  (40,126) | 1.62  (0.84,2.58) | 0.76  (0.71,0.81) | 647  (347,1109) | 35.04  (18.68,59.98) | 2208  (1186,3608) | 43.35  (23.29,70.48) | 0.56  (0.51,0.62) |
| Bosnia and Herzegovina | 19  (12,27) | 0.81  (0.52,1.18) | 38  (23,54) | 1.07  (0.66,1.53) | 1.01  (0.87,1.16) | 535  (343,777) | 21.78  (13.94,31.45) | 930  (575,1333) | 28.19  (17.41,40.64) | 1.02  (0.84,1.21) |
| Botswana | 2  (1,4) | 0.71  (0.39,1.15) | 9  (5,14) | 1.15  (0.7,1.74) | 2.08  (1.68,2.48) | 60  (32,99) | 18.09  (9.82,29.56) | 232  (134,378) | 27.2  (16.31,43.33) | 1.83  (1.41,2.26) |
| Brazil | 387  (270,532) | 0.83  (0.58,1.14) | 1275  (886,1704) | 0.91  (0.64,1.22) | 0.05  (-0.08,0.18) | 10514  (7461,14307) | 21.27  (14.98,29.11) | 33243  (23432,43860) | 23.89  (16.84,31.52) | 0.09  (-0.07,0.24) |
| Brunei Darussalam | 0  (0,0) | 0.51  (0.3,0.82) | 2  (1,3) | 0.84  (0.51,1.25) | 2.12  (1.96,2.27) | 9  (5,15) | 15.67  (9,25.41) | 55  (34,83) | 25.42  (15.71,38.17) | 2.08  (1.94,2.23) |
| Bulgaria | 116  (76,166) | 1.77  (1.16,2.52) | 193  (127,278) | 2.39  (1.56,3.41) | 1.56  (1.21,1.92) | 3298  (2159,4692) | 50.06  (32.97,71.14) | 4749  (3128,6745) | 66.95  (43.81,94.58) | 1.55  (1.17,1.94) |
| Burkina Faso | 3  (2,5) | 0.15  (0.09,0.23) | 10  (6,16) | 0.2  (0.12,0.33) | 1.16  (1.01,1.32) | 99  (61,157) | 3.99  (2.47,6.32) | 286  (168,476) | 5.33  (3.15,8.79) | 1.09  (0.91,1.27) |
| Burundi | 3(1,5) | 0.24  (0.12,0.4) | 7  (4,11) | 0.28  (0.15,0.46) | 0.01  (-0.18,0.21) | 89  (41,149) | 6.6  (3.06,10.93) | 193  (105,314) | 7.43  (4.04,12) | -0.14  (-0.35,0.08) |
| Cabo Verde | 1  (0,1) | 0.59  (0.35,0.9) | 2  (2,4) | 0.99  (0.61,1.54) | 1.74  (1.54,1.95) | 19  (11,29) | 14.89  (9.07,22.99) | 58  (36,90) | 22.84  (14.07,35.27) | 1.44  (1.25,1.63) |
| Cambodia | 7  (3,12) | 0.25  (0.12,0.41) | 30  (16,47) | 0.38  (0.21,0.6) | 1.27  (1.18,1.37) | 234  (111,403) | 7.81  (3.71,13.22) | 949  (506,1529) | 11.8  (6.35,19.02) | 1.24  (1.15,1.34) |
| Cameroon | 18  (10,28) | 0.8  (0.47,1.25) | 64  (36,105) | 1.05  (0.58,1.71) | 0.98  (0.93,1.02) | 500  (298,785) | 20.5  (12.16,31.98) | 1758  (975,2908) | 25.75  (14.32,42.35) | 0.78  (0.74,0.83) |
| Canada | 185  (130,255) | 0.99  (0.69,1.36) | 458  (317,614) | 1.14  (0.8,1.53) | 0.68  (0.42,0.95) | 4472  (3085,6142) | 25.29  (17.43,34.54) | 10887  (7657,14650) | 30.04  (21.22,40.16) | 0.78  (0.53,1.04) |
| Central African Republic | 2  (1,3) | 0.29  (0.17,0.44) | 6  (3,11) | 0.49  (0.27,0.81) | 1.73  (1.68,1.77) | 61  (36,97) | 8.48  (4.99,13.28) | 202  (108,343) | 13.97  (7.57,23.33) | 1.65  (1.59,1.7) |
| Chad | 4  (3,7) | 0.31  (0.18,0.5) | 14  (8,21) | 0.54  (0.31,0.86) | 2.02  (1.94,2.09) | 118  (70,191) | 8  (4.7,12.97) | 380  (221,605) | 13.78  (8.05,21.72) | 1.93  (1.85,2) |
| Chile | 41  (29,56) | 0.76  (0.53,1.03) | 102  (71,135) | 0.71  (0.5,0.94) | -0.06  (-0.28,0.17) | 1072  (745,1454) | 19.14  (13.3,25.95) | 2484  (1740,3275) | 17.92  (12.55,23.62) | -0.01  (-0.23,0.22) |
| China | 1222  (765,1846) | 0.27  (0.17,0.41) | 3628  (2212,5633) | 0.33  (0.2,0.51) | 0.22  (-0.09,0.53) | 39737  (24305,59873) | 8.44  (5.18,12.68) | 110987  (66012,173578) | 10.2  (6.1,15.89) | 0.3  (0,0.6) |
| Colombia | 49  (33,67) | 0.55  (0.37,0.75) | 218  (144,308) | 0.71  (0.47,1.01) | 0.5  (0.09,0.91) | 1391  (943,1913) | 14.44  (9.73,19.9) | 5812  (3882,8213) | 19.26  (12.86,27.24) | 0.62  (0.18,1.06) |
| Comoros | 0  (0,1) | 0.4  (0.2,0.64) | 2  (1,3) | 0.65  (0.38,1.02) | 1.44  (1.35,1.52) | 12  (6,19) | 10.64  (5.33,17.39) | 49  (28,75) | 17.19  (9.89,26.57) | 1.35  (1.23,1.46) |
| Congo | 3  (2,5) | 0.5  (0.29,0.79) | 14  (8,22) | 0.99  (0.59,1.53) | 2.05  (1.86,2.23) | 99  (58,161) | 14.69  (8.57,23.72) | 447  (251,723) | 27.55  (15.84,43.25) | 1.86  (1.65,2.07) |
| Cook Islands | 0  (0,0) | 0.79  (0.49,1.23) | 0  (0,0) | 0.71  (0.46,1.02) | -0.15  (-0.29,-0.02) | 2  (1,2) | 23.4  (14.73,36.13) | 3(2,4) | 20.97  (13.61,30.5) | -0.13  (-0.28,0.02) |
| Costa Rica | 6  (4,8) | 0.65  (0.45,0.9) | 33  (23,44) | 1.1  (0.75,1.45) | 1.42  (1.04,1.81) | 162  (110,223) | 17.51  (11.92,24.11) | 901  (617,1190) | 30.16  (20.71,39.65) | 1.51  (1.11,1.91) |
| Croatia | 43  (30,59) | 1.2  (0.81,1.64) | 75  (52,104) | 1.41  (0.97,1.93) | 0.73  (0.35,1.11) | 1145  (787,1566) | 31.39  (21.51,42.89) | 1782  (1232,2425) | 38.45  (26.9,51.68) | 0.89  (0.49,1.29) |
| Cuba | 67  (47,91) | 1.3  (0.9,1.76) | 250  (172,346) | 2.41  (1.66,3.33) | 1.88  (1.57,2.18) | 1983  (1358,2681) | 38.16  (26.13,51.66) | 6416  (4414,8907) | 66.3  (45.68,91.28) | 1.58  (1.24,1.93) |
| Cyprus | 4  (2,6) | 0.92  (0.54,1.57) | 10  (6,15) | 0.95  (0.59,1.41) | 0.11  (-0.01,0.23) | 91  (56,145) | 20.78  (12.73,33.5) | 242  (148,366) | 22.76  14,34.34) | 0.36  (0.24,0.48) |
| Czechia | 171  (115,234) | 2.04  (1.38,2.78) | 194  (127,273) | 1.53  (1.01,2.17) | -0.98  (-1.08,-0.89) | 4114  (2800,5556) | 52.14  (35.48,70.3) | 4333  (2851,6138) | 39.14  (25.73,55.78) | -0.94  (-1.05,-0.82) |
| Côte d'Ivoire | 5  (3,8) | 0.29  (0.18,0.44) | 23  (13,35) | 0.44  (0.26,0.66) | 1.59  (1.46,1.73) | 165  (103,241) | 7.72  (4.94,11.26) | 682  (389,1065) | 11.37  (6.49,17.63) | 1.58  (1.45,1.72) |
| Democratic People's Republic of Korea | 15  (8,24) | 0.15  (0.09,0.24) | 56  (32,93) | 0.29  (0.17,0.48) | 2.21  (2.17,2.26) | 458  (252,730) | 4.36  (2.44,6.92) | 1508  (867,2489) | 8.02  (4.63,13.3) | 2.01  (1.96,2.07) |
| Democratic Republic of the Congo | 21  (12,33) | 0.24  (0.14,0.38) | 99  (54,169) | 0.5  (0.27,0.86) | 2.5  (2.38,2.62) | 621  (362,991) | 6.45  (3.73,10.35) | 2913  (1599,5030) | 13.25  (7.27,22.9) | 2.46  (2.34,2.59) |
| Denmark | 47  (33,63) | 1.01  (0.7,1.37) | 56  (37,75) | 0.83  (0.56,1.11) | -0.67  (-0.78,-0.55) | 1090  (755,1471) | 26.17  (18.1,35.46) | 1230  (844,1643) | 21.26  (14.74,28.61) | -0.67  (-0.76,-0.57) |
| Djibouti | 0  (0,0) | 0.22  (0.11,0.4) | 1  (1,2) | 0.35  (0.19,0.63) | 1.49  (1.43,1.55) | 5  (2,9) | 5.96  (3.1,11.08) | 33  (18,59) | 9.61  (5.19,16.75) | 1.43  (1.36,1.5) |
| Dominica | 1  (0,1) | 1.4  (0.92,2.21) | 1  (1,1) | 2.1  (1.41,3.14) | 1.38  (1.28,1.48) | 12  (8,19) | 36.61  (24.28,56.59) | 23  (16,35) | 54.61  (36.48,81.62) | 1.35  (1.26,1.43) |
| Dominican Republic | 15  (9,22) | 0.76  (0.48,1.15) | 74  (45,115) | 1.41  (0.86,2.2) | 2.33  (2.2,2.46) | 494  (308,737) | 22.92  (14.42,34.68) | 2240  (1360,3506) | 42.22  (25.61,66.22) | 2.27  (2.15,2.39) |
| Ecuador | 47  (33,64) | 1.74  (1.2,2.36) | 119  (79,166) | 1.38  (0.91,1.93) | -0.23  (-0.93,0.48) | 1423  (981,1910) | 48.97(33.49,65.95) | 3300  (2240,4602) | 37.91  (25.72,52.87) | -0.33  (-1.04,0.37) |
| Egypt | 64  (41,93) | 0.5  (0.33,0.73) | 199  (130,285) | 0.73  (0.49,1.03) | 1.57  (1.43,1.71) | 2041  (1271,2924) | 13.41  (8.56,19.49) | 6281  (4121,9283) | 18.91  (12.6,27.17) | 1.35  (1.24,1.47) |
| El Salvador | 13  (8,17) | 0.79  (0.52,1.1) | 35  (23,51) | 1  (0.66,1.43) | 0.63  (0.48,0.79) | 358  (235,495) | 21.89  (14.35,30.28) | 967  (640,1394) | 27.72  (18.31,39.97) | 0.66  (0.5,0.82) |
| Equatorial Guinea | 0  (0,1) | 0.42  (0.24,0.7) | 2  (1,4) | 0.76  (0.39,1.26) | 2.05  (1.92,2.18) | 15  (8,24) | 12.24  (6.8,19.78) | 69  (34,115) | 20.74  (10.44,34.72) | 1.82  (1.69,1.95) |
| Eritrea | 2  (1,3) | 0.24  (0.12,0.37) | 6  (4,10) | 0.39  (0.22,0.61) | 1.63  (1.56,1.7) | 51  (27,83) | 6.7  (3.48,10.78) | 193  (106,313) | 10.65  (5.95,17.06) | 1.48  (1.39,1.56) |
| Estonia | 19  (13,25) | 1.41  (0.97,1.9) | 21  (14,29) | 1.22  (0.82,1.7) | -0.64  (-0.8,-0.48) | 487  (334,660) | 38.74  (26.4,52.4) | 485  (324,668) | 33.92  (22.55,46.68) | -0.68  (-0.86,-0.5) |
| Eswatini | 2  (1,3) | 1.25  (0.74,1.93) | 6  (3,11) | 2.05  (1.08,3.34) | 1.85  (1.34,2.37) | 49  (29,76) | 30.05  (17.74,46.57) | 171  (88,294) | 49.21  (25.43,83.67) | 1.86  (1.3,2.41) |
| Ethiopia | 21  (12,33) | 0.21  (0.12,0.33) | 37  (22,62) | 0.18  (0.1,0.29) | -1.17  (-1.4,-0.94) | 659  (370,1057) | 5.99  (3.4,9.41) | 1102  (637,1881) | 4.67  (2.71,7.95) | -1.42  (-1.67,-1.17) |
| Fiji | 3  (2,4) | 1.46  (0.87,2.31) | 10  (6,14) | 2.24  (1.38,3.33) | 1.48  (1.33,1.63) | 94  (56,146) | 43.27  (26.08,67.75) | 288  (177,435) | 64.29  (39.58,97.21) | 1.4  (1.25,1.55) |
| Finland | 48  (33,65) | 1.05  (0.73,1.42) | 88  (61,121) | 1.16  (0.8,1.57) | 0.39  (0.31,0.46) | 1067  (748,1455) | 25.79  (17.85,35.04) | 1802  (1260,2447) | 28.12  (19.86,37.92) | 0.33  (0.25,0.41) |
| France | 378  (264,507) | 0.75  (0.52,1.01) | 752  (499,1029) | 0.87  (0.59,1.17) | 0.86  (0.74,0.98) | 8151  (5700,10893) | 18.13  (12.67,24.52) | 15385  (10642,20581) | 21.74  (15.29,29.13) | 0.98  (0.86,1.1) |
| Gabon | 2  (1,4) | 0.7  (0.4,1.13) | 6  (3,9) | 1.05  (0.58,1.58) | 1.16  (0.93,1.39) | 63  (36,102) | 19.37  (11.12,31.07) | 170  (93,265) | 28.11  (15.63,43.12) | 1.01  (0.77,1.25) |
| Gambia | 1  (0,1) | 0.37  (0.22,0.57) | 3  (2,5) | 0.61  (0.36,0.94) | 1.43  (1.24,1.62) | 18  (11,27) | 10.23  (5.98,15.64) | 88  (53,136) | 16.5  (9.76,25.41) | 1.36  (1.15,1.58) |
| Georgia | 84  (58,111) | 2.2  (1.51,2.92) | 83  (56,115) | 2.36  (1.59,3.27) | 0.28  (-0.17,0.73) | 2394  (1663,3175) | 64.56  (44.9,85.26) | 2130  (1440,2969) | 65.07  (44,90.82) | -0.02  (-0.46,0.42) |
| Germany | 814  (551,1102) | 0.99  (0.67,1.34) | 867  (583,1184) | 0.78  (0.53,1.06) | -0.82  (-1.09,-0.54) | 18106  (12577,24395) | 24.35  (17.02,32.97) | 18669  (12852,25476) | 19.78  (13.74,26.77) | -0.71  (-0.98,-0.44) |
| Ghana | 15  (9,25) | 0.47  (0.26,0.76) | 108  (64,173) | 1.21  (0.7,1.9) | 3.21  (3.12,3.3) | 446  (257,746) | 12.55  (7.21,20.67) | 2972  (1754,4800) | 29.91  (17.64,48.19) | 2.91  (2.81,3.01) |
| Greece | 61  (42,81) | 0.72  (0.5,0.97) | 147  (101,197) | 1.11  (0.76,1.48) | 1.5  (1.29,1.71) | 1472  (1025,1973) | 18.09  (12.65,24.34) | 3221  (2231,4261) | 29.26  (20.46,38.66) | 1.72  (1.49,1.94) |
| Greenland | 0  (0,0) | 0.63  (0.39,0.94) | 0  (0,0) | 0.42  (0.26,0.65) | -1.31  (-1.42,-1.2) | 3  (2,4) | 16.2  (9.85,23.48) | 4  (2,6) | 11.05  (6.7,17.06) | -1.24  (-1.33,-1.15) |
| Grenada | 0  (0,1) | 1.26  (0.86,1.71) | 1  (1,2) | 2.07  (1.39,2.87) | 1.95  (1.7,2.2) | 13  (9,18) | 38.23  (26.06,52.15) | 35  (24,49) | 58.59  (39.62,81.33) | 1.73  (1.49,1.97) |
| Guam | 0  (0,1) | 1.05  (0.68,1.51) | 1  (1,1) | 0.94  (0.61,1.32) | 0.18  (-0.28,0.63) | 12  (8,18) | 29.38  (19.14,42.26) | 33  (22,46) | 30.95  (20.27,42.91) | 0.63  (0.18,1.07) |
| Guatemala | 20  (14,27) | 1.18  (0.82,1.6) | 71  (50,96) | 1.2  (0.84,1.62) | -0.31  (-0.56,-0.05) | 611  (427,820) | 31.23  (21.84,42) | 2053  (1450,2790) | 33  (23.22,44.76) | -0.21  (-0.47,0.05) |
| Guinea | 6  (4,10) | 0.37  (0.23,0.59) | 15  (9,23) | 0.53  (0.33,0.85) | 1.21  (1.17,1.24) | 180  (109,286) | 10.27  (6.22,16.28) | 438  (258,713) | 14.39  (8.63,23.01) | 1.16  (1.12,1.19) |
| Guinea-Bissau | 1  (1,2) | 0.49  (0.3,0.77) | 3  (2,5) | 0.77  (0.46,1.2) | 1.64  (1.59,1.69) | 31  (18,48) | 13.49  (8.14,20.97) | 91  (55,141) | 20.56  (12.31,32.31) | 1.49  (1.44,1.53) |
| Guyana | 3  (2,4) | 1.24  (0.82,1.78) | 8  (5,12) | 2.26  (1.48,3.24) | 1.99  (1.61,2.37) | 77  (52,112) | 35.47  (23.8,51.24) | 243  (156,355) | 65.22  (42.08,94.47) | 1.98  (1.58,2.38) |
| Haiti | 12  (7,20) | 0.68  (0.38,1.09) | 48  (26,82) | 1.14  (0.61,1.97) | 1.95  (1.84,2.05) | 412  (221,650) | 20.96  (11.37,33.35) | 1628  (882,2764) | 34.15  (18.37,58.71) | 1.9  (1.79,2) |
| Honduras | 14  (8,22) | 1.33  (0.8,2.1) | 91  (48,143) | 2.69  (1.4,4.18) | 2.34  (2.12,2.57) | 422  (256,661) | 37.05  (22.46,58.18) | 2626  (1392,4098) | 72.13  (38.13,112.49) | 2.19  (1.98,2.4) |
| Hungary | 164  (111,216) | 1.86  (1.25,2.46) | 182  (122,251) | 1.57  (1.06,2.16) | -0.96  (-1.34,-0.59) | 4014  (2764,5265) | 48.04  (32.56,63.27) | 4364  (2965,5968) | 43.03  (28.98,58.88) | -0.82  (-1.17,-0.47) |
| Iceland | 1  (1,2) | 0.82  (0.57,1.13) | 3  (2,4) | 0.89  (0.61,1.22) | 0.44  (0.3,0.58) | 32  (22,43) | 21.74  (15.17,29.65) | 64  (44,88) | 22.42  (15.43,30.53) | 0.24  (0.12,0.36) |
| India | 174  (106,251) | 0.08  (0.05,0.11) | 1074  (708,1566) | 0.17  (0.12,0.25) | 2.71  (2.55,2.87) | 5426  (3300,7906) | 2.13  (1.3,3.1) | 30628  (19689,44033) | 4.74  (3.06,6.84) | 2.58  (2.41,2.76) |
| Indonesia | 113  (69,169) | 0.2  (0.12,0.3) | 667  (343,1026) | 0.48  (0.25,0.74) | 3.06  (2.88,3.25) | 3958  (2336,6012) | 6.34  (3.83,9.47) | 22648  (11385,34810) | 15.27  (7.78,23.29) | 3.04  (2.82,3.26) |
| Iran (Islamic Republic of) | 26  (15,37) | 0.2  (0.12,0.29) | 137  (66,193) | 0.35  (0.17,0.5) | 2.08  (1.7,2.47) | 830  (486,1189) | 5.81  (3.39,8.36) | 4238  (1959,6105) | 10.04  (4.74,14.43) | 2.02  (1.62,2.43) |
| Iraq | 16  (9,31) | 0.4  (0.21,0.76) | 69  (43,106) | 0.57  (0.36,0.88) | 1.31  (1.15,1.46) | 496  (259,986) | 11.49  (6.03,22.71) | 2208  (1338,3395) | 16.18  (10.02,25.09) | 1.28  (1.12,1.44) |
| Ireland | 20  (14,27) | 0.86  (0.59,1.17) | 39  (27,54) | 0.91  (0.62,1.25) | 1.12  (0.69,1.55) | 468  (324,644) | 21.83(15.34,29.94) | 933  (638,1270) | 23.38  (16.04,31.83) | 1.15  (0.76,1.53) |
| Israel | 22  (15,30) | 0.84  (0.56,1.14) | 67  (46,90) | 0.94  (0.65,1.27) | 0.68  (0.46,0.9) | 533  (356,734) | 20.37  (13.62,28.01) | 1445  (980,1964) | 22.14  (15.03,30.17) | 0.6  (0.38,0.82) |
| Italy | 147  (103,196) | 0.28  (0.2,0.37) | 651  (435,902) | 0.77  (0.53,1.05) | 3.9  (3.21,4.59) | 3643  (2553,4872) | 7.5  (5.23,10.03) | 14926  (10366,20115) | 21.43  (15.01,28.79) | 4.07  (3.35,4.79) |
| Jamaica | 10  (7,13) | 1.04  (0.72,1.42) | 44  (28,61) | 2.71  (1.75,3.77) | 3.1  (2.68,3.53) | 262  (181,354) | 29.26  (20.21,39.54) | 1180  (769,1657) | 74.99  (48.67,105.41) | 3.01  (2.55,3.46) |
| Japan | 252  (188,337) | 0.26  (0.2,0.35) | 623  (418,851) | 0.37  (0.26,0.49) | 1.21  (1.01,1.41) | 6299  (4722,8418) | 6.71  (5.03,8.94) | 14011  (10073,18868) | 10.78  (7.75,14.28) | 1.7  (1.53,1.88) |
| Jordan | 5  (3,8) | 0.78  (0.48,1.18) | 26  (16,40) | 0.78  (0.48,1.18) | -0.19  (-0.61,0.23) | 154  (93,235) | 21.22  (12.92,32.23) | 813  (491,1240) | 20.86  (12.69,31.61) | -0.26  (-0.65,0.13) |
| Kazakhstan | 123  (85,166) | 1.58  (1.09,2.12) | 132  (91,175) | 1.22  (0.85,1.62) | -1  (-1.56,-0.43) | 3460  (2396,4662) | 43.92  (30.4,58.96) | 3843  (2679,5048) | 34.64  (24.23,45.42) | -1.03  (-1.58,-0.47) |
| Kenya | 7  (4,13) | 0.16  (0.09,0.3) | 45  (26,76) | 0.36  (0.21,0.6) | 3.1  (2.92,3.28) | 209  (120,400) | 4.47  (2.57,8.53) | 1392  (783,2358) | 10  (5.74,16.91) | 3.06  (2.89,3.22) |
| Kiribati | 0  (0,0) | 1.38  (0.53,2.25) | 1  (0,2) | 2.02  (0.7,3.42) | 1.3  (1.25,1.34) | 10  (4,16) | 43.04  (16.44,69.18) | 29  (10,50) | 60.29  (20.99,102.78) | 1.17  (1.12,1.21) |
| Kuwait | 1  (1,2) | 0.59  (0.42,0.78) | 17  (12,22) | 1.3  (0.94,1.68) | 3.46  (2.85,4.08) | 41  (29,54) | 16.47  (11.63,21.59) | 618  (451,792) | 37.96  (28.09,48.64) | 3.37  (2.75,4) |
| Kyrgyzstan | 22  (15,30) | 1.22  (0.82,1.67) | 33  (21,45) | 1.16  (0.73,1.59) | -0.24  (-0.52,0.05) | 644  (441,868) | 36.32  (24.85,49.06) | 1022  (649,1409) | 33.83  (21.46,46.62) | -0.33  (-0.6,-0.05) |
| Lao People's Democratic Republic | 3  (2,6) | 0.29  (0.15,0.52) | 12  (6,19) | 0.45  (0.24,0.74) | 1.51  (1.37,1.65) | 111  (52,198) | 8.94  (4.25,15.98) | 400  (208,655) | 14.05  (7.5,22.86) | 1.57  (1.41,1.73) |
| Latvia | 34  (24,45) | 1.47  (1.05,1.97) | 53  (36,72) | 2.04  (1.41,2.79) | 1.16  (0.91,1.42) | 905  (645,1217) | 41.25  (29.59,55.43) | 1208  (842,1643) | 55.12  (37.79,75.27) | 0.97  (0.7,1.24) |
| Lebanon | 7  (4,11) | 0.66  (0.4,1.04) | 21  (13,32) | 0.64  (0.41,0.97) | -0.04  (-0.16,0.07) | 203  (116,336) | 17.34  (9.95,28.28) | 523  (336,801) | 16.47  (10.51,25.27) | -0.14  (-0.22,-0.05) |
| Lesotho | 4  (2,6) | 0.69  (0.42,1.09) | 12  (6,19) | 1.75  (0.95,2.85) | 3.98  (3.45,4.51) | 94  (57,152) | 17.15  (10.34,27.67) | 306  (162,503) | 44.03  (23.4,72.24) | 4.06  (3.5,4.62) |
| Liberia | 3  (2,5) | 0.59  (0.38,0.88) | 9  (5,14) | 0.92  (0.51,1.45) | 1.78  (1.57,1.99) | 89  (56,132) | 16.03  (10.25,23.9) | 268  (151,430) | 24.1  (13.35,38.38) | 1.65  (1.44,1.87) |
| Libya | 5  (3,8) | 0.57  (0.33,0.88) | 26  (16,40) | 1.03  (0.63,1.54) | 2.28  (2.11,2.44) | 149  (89,227) | 15.99  (9.49,24.39) | 837  (511,1296) | 28.72  (17.75,43.68) | 2.23  (2.07,2.4) |
| Lithuania | 36  (25,48) | 1.28  (0.89,1.73) | 65  (45,88) | 1.79  (1.25,2.45) | 0.95  (0.73,1.17) | 946  (658,1282) | 35.01  (24.35,47.42) | 1498  (1051,2038) | 48.34  (33.78,66.77) | 0.86  (0.63,1.09) |
| Luxembourg | 5  (3,6) | 1.41  (0.96,1.92) | 7  (5,10) | 1.2  (0.82,1.64) | 0.17  (-0.08,0.43) | 107  (73,145) | 34.79  (23.71,47.07) | 158  (108,215) | 29.17  (20.16,39.6) | 0.13  (-0.13,0.39) |
| Madagascar | 7  (3,11) | 0.27  (0.14,0.43) | 26  (14,43) | 0.45  (0.24,0.74) | 1.58  (1.39,1.77) | 195  (100,316) | 7.16  (3.63,11.57) | 776  (414,1273) | 11.76  (6.29,19.5) | 1.53  (1.34,1.73) |
| Malawi | 3  (2,4) | 0.13  (0.08,0.2) | 10  (6,16) | 0.23  (0.13,0.37) | 1.81  (1.72,1.9) | 88  (54,134) | 3.67  (2.27,5.66) | 329  (181,550) | 6.85  (3.75,11.2) | 1.86  (1.76,1.96) |
| Malaysia | 23  (15,33) | 0.48  (0.31,0.68) | 116  (74,164) | 0.8  (0.52,1.14) | 1.66  (1.57,1.76) | 727  (467,1032) | 14  (9.17,19.76) | 3415  (2239,4787) | 22.47  (14.55,31.46) | 1.55  (1.46,1.64) |
| Maldives | 0  (0,0) | 0.19  (0.09,0.32) | 0  (0,0) | 0.15  (0.09,0.23) | -1.07  (-1.22,-0.92) | 3  (1,5) | 6.21  (2.74,10.48) | 9  (5,13) | 4.79  (2.98,7.36) | -1.12  (-1.25,-0.99) |
| Mali | 6  (4,9) | 0.3  (0.18,0.44) | 16  (9,23) | 0.35  (0.21,0.55) | 0.59  (0.49,0.69) | 180  (107,274) | 8.16  (4.88,12.39) | 475  (285,714) | 9.72  (5.76,14.59) | 0.61  (0.5,0.72) |
| Malta | 2  (1,3) | 0.89  (0.6,1.22) | 7  (5,9) | 1.18  (0.82,1.64) | 0.77  (0.56,0.98) | 53  (36,73) | 22.13  (15.19,30.5) | 145  (101,201) | 30.15  (21.08,41.36) | 0.87  (0.66,1.08) |
| Marshall Islands | 0  (0,0) | 1.69  (0.9,2.72) | 1  (0,1) | 2.71  (1.11,5.85) | 1.43  (1.23,1.62) | 5  (2,7) | 50.18  (27,81.64) | 18  (7,37) | 81.57  (33.3,174.74) | 1.46  (1.25,1.67) |
| Mauritania | 4  (2,6) | 0.78  (0.45,1.21) | 11  (6,17) | 1.05  (0.58,1.63) | 0.94  (0.85,1.03) | 108  (61,168) | 20.23  (11.49,31.47) | 291  (163,451) | 25.71  (14.49,39.84) | 0.69  (0.6,0.78) |
| Mauritius | 5  (3,6) | 1.16  (0.79,1.56) | 15  (10,20) | 1.46  (1.01,1.97) | 0.03  (-0.62,0.69) | 147  (100,199) | 34.42  (23.47,46.78) | 435  (302,590) | 44.26  (31.04,59.87) | 0.05  (-0.62,0.73) |
| Mexico | 117  (81,156) | 0.55  (0.38,0.75) | 546  (369,741) | 0.79  (0.53,1.07) | 1.51  (0.94,2.08) | 3290  (2302,4369) | 14.04  (9.79,18.68) | 16243  (10976,22150) | 22.87  (15.46,31.16) | 1.96  (1.38,2.54) |
| Micronesia (Federated States of) | 0  (0,1) | 1.67  (0.9,2.67) | 1  (1,2) | 2.31  (1.19,3.79) | 1.06  (1.02,1.1) | 14  (7,22) | 51.23  (27.47,83.5) | 32  (16,53) | 70.27  (35.81,115.75) | 1.04  (0.99,1.08) |
| Monaco | 0  (0,0) | 0.43  (0.25,0.69) | 0  (0,0) | 0.57  (0.34,0.87) | 1.1  (0.95,1.26) | 4  (2,6) | 10.98  (6.33,17.39) | 7  (4,10) | 14.73  (8.84,22.12) | 1.09  (0.92,1.26) |
| Mongolia | 4  (2,6) | 0.68  (0.38,1.08) | 9  (5,14) | 0.66  (0.4,1.04) | -0.42  (-0.59,-0.25) | 121  (68,194) | 20.4  (11.55,32.95) | 292  (168,460) | 19.21  (11.24,29.69) | -0.51  (-0.69,-0.33) |
| Montenegro | 3  (2,5) | 0.97  (0.57,1.55) | 8  (5,11) | 1.4  (0.88,2.1) | 1.23  (1.07,1.4) | 93  (55,149) | 26.47  (15.71,42.33) | 189  (119,283) | 35.39  (22.16,52.87) | 1.04  (0.82,1.26) |
| Morocco | 11  (6,18) | 0.16  (0.09,0.25) | 46  (27,70) | 0.26  (0.15,0.4) | 1.81  (1.73,1.89) | 331  (191,526) | 4.37  (2.5,6.99) | 1370  (783,2111) | 7.39  (4.27,11.29) | 1.87  (1.79,1.95) |
| Mozambique | 10  (4,17) | 0.29  (0.14,0.5) | 35  (17,65) | 0.55  (0.27,1.02) | 2.37  (2.14,2.6) | 291  (127,511) | 8.21  (3.65,14.48) | 1088  (529,2020) | 15.29  (7.47,28.64) | 2.37  (2.14,2.6) |
| Myanmar | 49  (25,82) | 0.36  (0.19,0.6) | 127  (75,201) | 0.43  (0.26,0.67) | 0.23  (0.05,0.4) | 1683  (829,2884) | 11.87  (5.95,20.26) | 4186  (2353,6697) | 13.71  (7.79,21.86) | 0.12  (-0.07,0.31) |
| Namibia | 2  (1,2) | 0.44  (0.27,0.72) | 6  (3,10) | 0.78  (0.43,1.25) | 1.74  (1.49,1.98) | 43  (26,68) | 11.49  (7.04,18.29) | 166  (87,263) | 19.65  (10.33,31.26) | 1.56  (1.29,1.82) |
| Nauru | 0  (0,0) | 2.21  (0.99,4.01) | 0  (0,0) | 2.86  (1.33,4.82) | 0.87  (0.78,0.95) | 2  (1,3) | 67.29  (29.2,120.42) | 3  (1,6) | 86.51  (39.4,149.28) | 0.85  (0.76,0.94) |
| Nepal | 4  (2,7) | 0.07  (0.04,0.14) | 14  (7,32) | 0.11  (0.06,0.24) | 1.61  (1.19,2.04) | 116  (56,239) | 2.14  (1.03,4.36) | 460  (227,1028) | 3.35  (1.66,7.44) | 1.67  (1.22,2.12) |
| Netherlands | 92  (64,127) | 0.77  (0.53,1.06) | 164  (111,224) | 0.82  (0.56,1.1) | 0.29  (0.21,0.38) | 2027  (1415,2803) | 18.58  (12.95,25.71) | 3435  (2375,4668) | 19.39  (13.65,26.29) | 0.3  (0.18,0.42) |
| New Zealand | 23  (16,31) | 1.07  (0.74,1.43) | 56  (39,75) | 1.24  (0.88,1.65) | 0.98  (0.79,1.17) | 571  (396,768) | 27.79  (19.29,37.31) | 1357  (965,1800) | 32.62  (23.17,43.02) | 0.98  (0.8,1.16) |
| Nicaragua | 3  (2,5) | 0.39  (0.26,0.57) | 14  (9,21) | 0.53  (0.33,0.77) | 1.27  (1.12,1.42) | 95  (63,136) | 10.89  (7.22,15.69) | 406  (258,589) | 14.48  (9.17,21.07) | 1.22  (1.06,1.38) |
| Niger | 4  (3,7) | 0.34  (0.19,0.54) | 18  (10,29) | 0.43  (0.24,0.69) | 0.76  (0.66,0.85) | 133  (76,205) | 9.16  (5.2,14.32) | 513  (286,840) | 11.12  (6.17,18.17) | 0.59  (0.49,0.69) |
| Nigeria | 39  (23,64) | 0.19  (0.11,0.31) | 164  (98,270) | 0.36  (0.22,0.58) | 2.18  (2.12,2.24) | 985  (591,1624) | 4.6  (2.76,7.54) | 4551  (2669,7688) | 8.65  (5.13,14.37) | 2.11  (2.04,2.18) |
| Niue | 0  (0,0) | 1.33  (0.75,2.07) | 0  (0,0) | 1.97  (0.92,3.5) | 1.18  (1.12,1.25) | 0  (0,1) | 39.35  (22.54,62.03) | 1  (0,1) | 58.25  (27.16,105.63) | 1.11  (1.04,1.18) |
| North Macedonia | 15  (10,21) | 1.52  (0.98,2.19) | 33  (21,49) | 1.94  (1.24,2.88) | 0.73  (0.5,0.96) | 401  (261,589) | 39.38  (25.73,57.53) | 842  (533,1271) | 47.98  (30.53,72.44) | 0.63  (0.41,0.85) |
| Northern Mariana Islands | 0  (0,0) | 2.11  (1.17,3.96) | 1  (1,1) | 3.29  (2.14,4.68) | 1.98  (1.73,2.23) | 6  (3,11) | 63.65  (35.07,117.69) | 29  (18,42) | 97.32  (62.3,139.13) | 1.91  (1.67,2.15) |
| Norway | 35  (24,47) | 0.89  (0.61,1.2) | 42  (29,56) | 0.72  (0.5,0.96) | -0.57  (-0.76,-0.39) | 770  (531,1038) | 22.63  (15.66,30.56) | 876  (618,1178) | 17.42  (12.28,23.21) | -0.67  (-0.91,-0.42) |
| Oman | 0  (0,1) | 0.14  (0.08,0.21) | 2  (1,2) | 0.2  (0.13,0.29) | 1.68  (1.47,1.9) | 12  (7,20) | 3.91  (2.18,6.29) | 55  (35,81) | 5.68  (3.7,8.37) | 1.55  (1.31,1.79) |
| Pakistan | 106  (71,166) | 0.42  (0.28,0.67) | 624  (381,925) | 1.06  (0.64,1.58) | 3.16  (2.91,3.41) | 3142  (2113,4824) | 11.45  (7.68,17.88) | 19084  (11778,28446) | 28.65  (17.61,42.54) | 3.12  (2.87,3.38) |
| Palau | 0  (0,0) | 0.24  (0.14,0.38) | 0  (0,0) | 0.3  (0.18,0.44) | 0.76  (0.7,0.83) | 0  (0,1) | 7  (4.05,11.02) | 1  (1,1) | 8.09  (5.01,12.04) | 0.51  (0.45,0.57) |
| Palestine | 7  (4,10) | 1.44  (0.88,2.21) | 22  (14,32) | 1.75  (1.07,2.49) | 0.8  (0.7,0.91) | 198  (119,305) | 39.26  (23.91,60.32) | 676  (421,975) | 47.24  (29.25,67.12) | 0.73  (0.63,0.82) |
| Panama | 5  (4,7) | 0.73  (0.5,1.01) | 30  (21,41) | 1.33  (0.92,1.82) | 2.02  (1.76,2.28) | 135  (92,185) | 18.09  (12.39,24.88) | 788  (539,1081) | 35.03  (24,48.07) | 2.23  (1.96,2.5) |
| Papua New Guinea | 6  (3,10) | 0.58  (0.29,1.01) | 22  (10,41) | 0.82  (0.38,1.45) | 1.1  (1.06,1.14) | 189  (87,334) | 17.8  (8.3,31.6) | 776  (341,1433) | 25.34  (11.5,45.83) | 1.12  (1.08,1.16) |
| Paraguay | 12  (7,18) | 1.03  (0.63,1.55) | 40  (24,62) | 1.32  (0.79,2.04) | 0.71  (0.61,0.82) | 321  (199,480) | 27.14  (16.75,40.7) | 1045  (633,1670) | 33.69  (20.41,53.86) | 0.56  (0.46,0.67) |
| Peru | 57  (35,83) | 0.92  (0.56,1.35) | 177  (109,283) | 1.01  (0.62,1.63) | -0.12  (-0.37,0.13) | 1704  (1025,2529) | 26  (15.64,38.42) | 4992  (3046,8003) | 28.17  (17.19,45.35) | -0.2  (-0.45,0.06) |
| Philippines | 47  (30,67) | 0.28  (0.18,0.41) | 257  (164,381) | 0.55  (0.35,0.81) | 2.22  (2.04,2.4) | 1641  (1039,2357) | 8.89  (5.63,12.67) | 8474  (5243,12556) | 17.19  (10.77,25.47) | 2.17  (2,2.35) |
| Poland | 375  (267,499) | 1.44  (1.02,1.9) | 920  (638,1242) | 2.05  (1.44,2.77) | 1.09  (0.88,1.31) | 9497  (6759,12499) | 37.46  (26.63,49.3) | 19583  (13695,26432) | 48.42  (33.82,65.17) | 0.78  (0.62,0.95) |
| Portugal | 77  (54,109) | 0.96  (0.68,1.35) | 145  (100,202) | 0.97  (0.68,1.35) | 0.13  (0.01,0.25) | 1903  (1358,2657) | 24.83  (17.74,34.57) | 3101  (2169,4245) | 24.84  (17.52,33.96) | 0.13  (0.01,0.25) |
| Puerto Rico | 17  (12,22) | 0.87  (0.62,1.15) | 53  (37,72) | 1.39  (0.98,1.89) | 1.11(0.84,1.38) | 445  (314,592) | 22.99  (16.24,30.55) | 1274  (903,1718) | 39.3  (27.85,53.1) | 1.32  (1.04,1.59) |
| Qatar | 0  (0,1) | 1.07  (0.59,1.94) | 4  (2,5) | 1.39  (0.92,1.99) | 1.31  (0.83,1.78) | 12  (7,21) | 28.18  (15.73,49.82) | 128  (82,204) | 36.16  (23.77,52.19) | 1.3  (0.89,1.7) |
| Republic of Korea | 46  (24,74) | 0.26  (0.14,0.42) | 90  (51,143) | 0.17  (0.1,0.27) | -1.15  (-1.66,-0.63) | 1388  (687,2218) | 7.41  (3.77,11.84) | 2326  (1288,3696) | 4.85  (2.7,7.64) | -1.06  (-1.63,-0.48) |
| Republic of Moldova | 36  (25,47) | 1.35  (0.96,1.77) | 53  (38,68) | 1.48  (1.05,1.89) | 0.19  (-0.03,0.41) | 1041  (737,1367) | 38.44  (27.3,50.46) | 1477  (1048,1903) | 43.09  (30.53,55.59) | 0.21  (0.01,0.41) |
| Romania | 160  (106,214) | 1.03  (0.68,1.38) | 265  (183,358) | 1.26  (0.87,1.72) | 0.27  (0.03,0.5) | 4418  (2929,5971) | 28.67  (19,38.87) | 6543  (4513,8906) | 34.87  (24.04,47.54) | 0.21  (-0.02,0.43) |
| Russian Federation | 2082  (1494,2745) | 1.77  (1.27,2.34) | 3329  (2376,4318) | 2.2  (1.56,2.85) | 0.17  (-0.09,0.43) | 59541  (42502,77842) | 52.94  (37.79,69.43) | 89239  (62972,116222) | 63.38  (44.5,82.66) | 0.04  (-0.24,0.33) |
| Rwanda | 6  (3,9) | 0.35  (0.17,0.56) | 15  (8,25) | 0.41  (0.23,0.67) | -0.26  (-0.6,0.08) | 177  (81,279) | 9.9  (4.59,15.71) | 445  (242,725) | 10.91  (5.99,17.75) | -0.51  (-0.88,-0.13) |
| Saint Kitts and Nevis | 0  (0,1) | 1.81  (1.21,2.46) | 1  (1,1) | 2.48  (1.67,3.32) | 1.29  (0.97,1.61) | 10  (7,13) | 51.57  (35.17,69.65) | 24  (17,33) | 62.53  (42.64,83.82) | 0.75  (0.49,1.01) |
| Saint Lucia | 1  (0,1) | 1.12  (0.77,1.55) | 2  (1,3) | 1.6  (1.06,2.23) | 0.43  (0.16,0.69) | 16  (11,22) | 33.4  (23.03,46.03) | 56  (38,78) | 45.54  (30.6,63.19) | 0.48  (0.27,0.7) |
| Saint Vincent and the Grenadines | 0  (0,1) | 0.97  (0.66,1.39) | 1  (1,2) | 1.52  (1.03,2.18) | 1.46  (1.29,1.63) | 11  (7,15) | 27.69  (18.8,39.22) | 31  (21,44) | 43.92  (29.52,63.04) | 1.46  (1.31,1.61) |
| Samoa | 1  (0,1) | 1.5  (0.9,2.34) | 2  (1,2) | 2.12  (1.32,3.28) | 1.14  (1.07,1.21) | 20  (12,33) | 42.64  (25.31,69.56) | 47  (29,75) | 60.25  (37.39,95.72) | 1.14  (1.07,1.21) |
| San Marino | 0  (0,0) | 0.26  (0.15,0.4) | 0  (0,0) | 0.2  (0.1,0.34) | 0.18  (-0.13,0.49) | 1  (1,2) | 6.22  (3.68,9.85) | 2  (1,3) | 5.11  (2.59,8.63) | 0.42  (0.09,0.75) |
| Sao Tome and Principe | 0  (0,0) | 0.82  (0.52,1.18) | 1  (0,1) | 1.4  (0.84,2.12) | 1.59  (1.48,1.71) | 7  (5,11) | 20.94  (13.15,30.1) | 21  (13,31) | 34.56  (20.93,52.31) | 1.45  (1.31,1.59) |
| Saudi Arabia | 8  (5,13) | 0.34  (0.19,0.53) | 51  (33,75) | 0.64  (0.42,0.93) | 2.21  (2.04,2.38) | 254  (144,405) | 9.26  (5.23,14.98) | 1901  (1219,2898) | 18.24  (11.84,26.88) | 2.37  (2.19,2.54) |
| Senegal | 7  (4,11) | 0.44  (0.27,0.67) | 29  (17,45) | 0.73  (0.44,1.12) | 1.86  (1.69,2.03) | 209  (129,316) | 12.12  (7.39,18.32) | 825  (476,1273) | 19.11  (11.24,29.29) | 1.67  (1.49,1.84) |
| Serbia | 82  (48,127) | 1.38  (0.81,2.16) | 154  (96,229) | 1.65  (1.04,2.45) | 0.29  (0.15,0.43) | 2200  (1279,3405) | 34.83  (20.26,54.85) | 3681  (2293,5466) | 43.02  (26.65,64.09) | 0.46  (0.31,0.6) |
| Seychelles | 0  (0,0) | 0.88  (0.57,1.3) | 1  (0,1) | 1.05  (0.7,1.52) | 0.92  (0.79,1.05) | 8  (5,12) | 27.86  (17.79,41.03) | 20  (14,29) | 31.62  (21.49,45.17) | 0.79  (0.68,0.9) |
| Sierra Leone | 3  (2,5) | 0.31  (0.18,0.5) | 10  (6,16) | 0.57  (0.35,0.89) | 2.29  (2.16,2.42) | 81  (48,128) | 8.06  (4.83,12.84) | 286  (174,440) | 14.68  (8.84,22.79) | 2.3  (2.17,2.43) |
| Singapore | 3  (2,4) | 0.25  (0.18,0.34) | 18  (12,25) | 0.41  (0.27,0.55) | 1.92  (1.74,2.1) | 96  (69,131) | 7.34  (5.26,10.01) | 508  (334,683) | 11.64  (7.68,15.67) | 1.87  (1.68,2.06) |
| Slovakia | 85  (54,122) | 2.42  (1.53,3.48) | 115  (72,172) | 2.04  (1.28,3.07) | -0.64  (-0.7,-0.58) | 2140  (1379,3046) | 63.04  (40.41,89.99) | 2734  (1716,4217) | 52.18  (32.88,81.1) | -0.68  (-0.75,-0.62) |
| Slovenia | 21  (15,28) | 1.38  (0.98,1.86) | 36  (25,50) | 1.39  (0.96,1.9) | -0.29  (-0.56,-0.02) | 515  (365,695) | 35.69  (25.42,48.03) | 787  (543,1078) | 35.27  (24.29,48.34) | -0.31  (-0.59,-0.02) |
| Solomon Islands | 1  (0,1) | 0.88  (0.37,1.48) | 3  (1,4) | 1.37  (0.67,2.22) | 1.44  (1.28,1.6) | 21  (8,37) | 27.51  (10.62,47.61) | 95  (45,160) | 43.8  (21.28,71.85) | 1.52  (1.35,1.68) |
| Somalia | 5  (2,8) | 0.33  (0.16,0.56) | 17  (8,28) | 0.44  (0.22,0.73) | 0.9  (0.86,0.93) | 165  (74,277) | 9.93  (4.61,16.87) | 565  (284,960) | 12.89  (6.48,21.58) | 0.79  (0.75,0.83) |
| South Africa | 72  (48,110) | 0.62  (0.41,0.94) | 312  (206,416) | 1.18  (0.77,1.58) | 2.44  (2.16,2.72) | 1997  (1343,2962) | 16.19  (10.87,24.3) | 8102  (5453,10721) | 29.3  (19.53,38.81) | 2.32  (2.07,2.57) |
| South Sudan | 2  (1,4) | 0.22  (0.12,0.36) | 5  (3,9) | 0.28  (0.15,0.46) | 0.66  (0.45,0.86) | 69  (36,108) | 6.11  (3.2,9.82) | 172  (86,293) | 7.75  (3.91,12.92) | 0.63  (0.4,0.86) |
| Spain | 339  (233,464) | 1.06  (0.74,1.46) | 599  (404,811) | 1.04  (0.71,1.38) | 0.13  (0.02,0.23) | 8137  (5635,11176) | 27.29  (19.03,37.37) | 13033  (9210,17164) | 26.85  (18.97,35.46) | 0.18  (0.06,0.3) |
| Sri Lanka | 13  (8,20) | 0.24  (0.15,0.37) | 51  (28,83) | 0.33  (0.19,0.54) | 0.86  (0.66,1.06) | 420  (262,650) | 6.95  (4.3,10.68) | 1415  (788,2365) | 9.23  (5.15,15.45) | 0.71  (0.5,0.93) |
| Sudan | 12  (7,23) | 0.27  (0.15,0.52) | 41  (23,71) | 0.43  (0.24,0.76) | 1.67  (1.52,1.82) | 378  (196,724) | 7.68  (4.06,14.64) | 1398  (748,2339) | 12.58  (7.01,21.68) | 1.71  (1.56,1.87) |
| Suriname | 1  (0,1) | 0.48  (0.31,0.72) | 3  (2,4) | 0.78  (0.48,1.18) | 1.79  (1.58,1.99) | 19  (12,30) | 13.64  (8.76,20.97) | 79  (48,120) | 22.77  (13.85,34.61) | 1.77  (1.56,1.97) |
| Sweden | 74  (50,102) | 0.83  (0.56,1.13) | 110  (73,154) | 0.85  (0.57,1.2) | 0.1  (-0.04,0.25) | 1608  (1084,2182) | 20.72  (14.12,28.32) | 2188  (1474,3119) | 20.1  (13.42,28.5) | -0.02  (-0.15,0.11) |
| Switzerland | 51  (34,70) | 0.8  (0.54,1.08) | 71  (48,99) | 0.66  (0.44,0.92) | -0.21  (-0.48,0.06) | 1110  (775,1498) | 19.62  (13.77,26.43) | 1482  (1008,2030) | 16.4  (11.07,22.23) | -0.19  (-0.42,0.05) |
| Syrian Arab Republic | 12  (7,18) | 0.46  (0.28,0.71) | 38  (24,60) | 0.6  (0.38,0.92) | 0.43  (0.18,0.68) | 368  (233,547) | 13.03  (8.24,19.73) | 1198  (750,1898) | 16.57  (10.39,26.02) | 0.36  (0.1,0.62) |
| Taiwan (Province of China) | 9  (6,13) | 0.12  (0.09,0.17) | 94  (64,128) | 0.42  (0.29,0.58) | 4.23  (3.85,4.6) | 289  (196,385) | 3.57  (2.42,4.75) | 2909  (1997,4009) | 13.79  (9.48,19.02) | 4.6  (4.23,4.97) |
| Tajikistan | 15  (9,23) | 1.01  (0.61,1.48) | 30  (16,53) | 0.94  (0.54,1.58) | -0.32  (-0.48,-0.17) | 467  (277,685) | 29.82  (17.68,43.79) | 987  (523,1832) | 27.4  (14.97,48.47) | -0.39  (-0.5,-0.28) |
| Thailand | 44  (26,65) | 0.22  (0.13,0.32) | 271  (142,418) | 0.46  (0.24,0.71) | 2.1  (1.92,2.29) | 1449  (869,2136) | 6.68  (4.02,9.76) | 8184  (4243,12770) | 14.21  (7.41,22.11) | 2.13  (1.91,2.35) |
| Timor-Leste | 0  (0,0) | 0.11  (0.06,0.18) | 1  (0,1) | 0.18  (0.1,0.3) | 1.84  (1.56,2.11) | 6  (3,10) | 3.2  (1.71,5.36) | 27  (15,45) | 5.9  (3.22,9.83) | 2.02  (1.71,2.33) |
| Togo | 3  (2,4) | 0.4  (0.25,0.63) | 15  (8,23) | 0.71  (0.4,1.08) | 1.93  (1.84,2.02) | 76  (48,117) | 10.73  (6.82,16.52) | 418  (235,658) | 17.95  (10.11,27.82) | 1.78  (1.7,1.87) |
| Tokelau | 0  (0,0) | 1.37  (0.77,2.21) | 0  (0,0) | 1.79  (0.92,2.93) | 0.81  (0.77,0.85) | 0  (0,0) | 41.67  (23.26,67.69) | 0  (0,1) | 54.94  (28.2,89.59) | 0.8  (0.76,0.84) |
| Tonga | 0  (0,1) | 1.35  (0.77,2.01) | 1  (0,1) | 1.74  (0.86,2.66) | 0.82  (0.78,0.87) | 13  (7,19) | 41.63  (23.19,60.41) | 22  (11,34) | 51.62  (25.55,78.36) | 0.65  (0.61,0.7) |
| Trinidad and Tobago | 7  (5,10) | 1.66  (1.17,2.21) | 26  (17,36) | 2.5  (1.66,3.52) | 1.01  (0.74,1.28) | 212  (151,283) | 46.96  (33.48,63.14) | 707  (469,1005) | 71.09  (47.04,101.34) | 1.02  (0.73,1.31) |
| Tunisia | 6  (4,10) | 0.26  (0.16,0.4) | 26  (16,41) | 0.38  (0.23,0.59) | 1.17  (1.13,1.22) | 187  (121,277) | 7.13  (4.56,10.57) | 739  (448,1193) | 10.41  (6.32,16.75) | 1.17  (1.13,1.21) |
| Turkey | 190  (113,286) | 1.04  (0.62,1.56) | 514  (310,739) | 1.02  (0.62,1.47) | 0.01  (-0.39,0.42) | 5616  (3371,8381) | 28.74  (17.19,42.92) | 14162  (8574,20522) | 27.88  (16.9,40.34) | -0.12  (-0.49,0.24) |
| Turkmenistan | 8  (5,11) | 0.7  (0.48,0.97) | 13  (8,19) | 0.55  (0.35,0.79) | -0.93  (-1.59,-0.26) | 238  (163,332) | 20.4  (13.98,28.44) | 408  (263,589) | 16.56  (10.67,23.91) | -0.81  (-1.46,-0.16) |
| Tuvalu | 0  (0,0) | 1.35  (0.75,2.08) | 0  (0,0) | 1.92  (0.95,3.08) | 1.16  (1.12,1.2) | 2  (1,3) | 41.1  (22.8,63.83) | 3  (2,5) | 57.78  (28.52,93.08) | 1.1  (1.06,1.14) |
| Uganda | 11  (7,18) | 0.34  (0.2,0.53) | 58  (35,87) | 0.69  (0.43,1.02) | 1.96  (1.75,2.16) | 335  (195,526) | 9.35  (5.48,14.6) | 1771  (1055,2693) | 18.95  (11.41,28.57) | 1.91  (1.68,2.14) |
| Ukraine | 683  (469,898) | 1.46  (1.01,1.93) | 924  (559,1426) | 1.95  (1.16,2.97) | 0.22  (-0.76,1.21) | 18294  (12709,24139) | 40.85  (28.31,54.11) | 24465  (14555,37532) | 55.63  (32.7,85.1) | 0.25  (-0.76,1.28) |
| United Arab Emirates | 3  (1,5) | 1.87  (0.93,3.24) | 26  (17,40) | 5.35  (3.45,8.09) | 4.95  (4.43,5.47) | 96  (46,160) | 52.92  (25.36,90.82) | 893  (574,1347) | 112.77  (73.91,170.03) | 3.59  (3.21,3.97) |
| United Kingdom | 454  (322,619) | 0.84  (0.6,1.14) | 965  (666,1283) | 1.3  (0.91,1.73) | 2.39  (2.06,2.73) | 10177  (7245,13638) | 21.16  (15.1,28.36) | 20420  (14306,26970) | 31.42  (22.21,41.55) | 2.3  (1.95,2.64) |
| United Republic of Tanzania | 26  (13,42) | 0.47  (0.23,0.73) | 102  (60,158) | 0.77  (0.46,1.21) | 1.8  (1.72,1.88) | 785  (389,1205) | 12.87  (6.35,19.93) | 2977  (1778,4684) | 20.51  (12.12,31.97) | 1.67  (1.59,1.76) |
| United States of America | 2009  (1406,2722) | 1.06  (0.75,1.43) | 5242  (3707,6683) | 1.62  (1.16,2.06) | 1.47  (1.36,1.58) | 49405  (35142,66146) | 28.44  (20.36,37.69) | 136850  (99557,171971) | 46.19  (33.84,58.22) | 1.73  (1.63,1.83) |
| United States Virgin Islands | 1  (0,1) | 1.56  (1.03,2.27) | 1  (1,2) | 1.05  (0.62,1.78) | -1.11  (-1.31,-0.9) | 21  (14,30) | 42.13  (28.55,60.57) | 25  (15,43) | 29.91  (17.32,52.4) | -0.85  (-1.03,-0.67) |
| Uruguay | 22  (15,29) | 0.96  (0.67,1.3) | 28  (20,38) | 0.88  (0.61,1.19) | -0.47  (-0.56,-0.37) | 520  (361,710) | 24.59  (17.05,33.55) | 642  (447,864) | 22.76  (15.89,30.39) | -0.39  (-0.5,-0.29) |
| Uzbekistan | 53  (36,74) | 0.8  (0.54,1.13) | 115  (74,164) | 0.75  (0.49,1.08) | 0.07  (-0.2,0.35) | 1567  (1045,2198) | 23.48  (15.67,32.99) | 3768  (2467,5370) | 22.93  (15.05,32.55) | 0.27  (0.05,0.49) |
| Vanuatu | 0  (0,0) | 0.81  (0.39,1.33) | 1  (1,2) | 1.29  (0.63,2.04) | 1.42  (1.32,1.52) | 8  (4,14) | 23.9  (11.47,41.4) | 40  (20,65) | 38.42  (18.76,61.16) | 1.41  (1.29,1.54) |
| Venezuela (Bolivarian Republic of) | 64  (45,86) | 1.27  (0.89,1.72) | 199  (125,288) | 1.21  (0.76,1.75) | -0.12  (-0.48,0.25) | 1807  (1275,2397) | 33.88  (23.92,45.12) | 5424  (3413,7917) | 32.69  (20.51,47.74) | -0.12  (-0.51,0.28) |
| Viet Nam | 12  (7,18) | 0.05  (0.03,0.08) | 68  (40,111) | 0.12  (0.07,0.19) | 3.31  (3.08,3.54) | 323  (204,509) | 1.37  (0.87,2.15) | 2026  (1182,3255) | 3.4  (1.98,5.49) | 3.63  (3.35,3.91) |
| Yemen | 4  (2,8) | 0.15  (0.08,0.31) | 19  (11,35) | 0.26  (0.14,0.49) | 1.85  (1.64,2.05) | 123  (61,235) | 4.39  (2.17,8.77) | 627  (341,1114) | 7.43  (4.07,13.26) | 1.78  (1.58,1.97) |
| Zambia | 5  (3,9) | 0.39  (0.19,0.63) | 34  (16,73) | 0.91  (0.44,1.91) | 2.67  (2.47,2.87) | 171  (81,277) | 10.81  (5.27,17.67) | 1047  (465,2382) | 25.22  (11.62,55.39) | 2.65  (2.43,2.87) |
| Zimbabwe | 13  (8,20) | 0.65  (0.4,0.99) | 78  (46,117) | 1.92  (1.15,2.92) | 4.64  (3.89,5.4) | 386  (230,587) | 17.33  (10.37,26.61) | 2329  (1354,3545) | 51.55  (30.36,77.38) | 4.68  (3.86,5.52) |

BMI, body mass index; DALYs, disability-adjusted life years; ASMR, age-standardized mortality rate; ASDR, age-standardized DALY rate; EAPC, estimated annual percentage change; UI, uncertainty interval;

CI, confidence interval.
